# Brg1 chromatin remodeling ATPase balances germ layer patterning by amplifying the transcriptional burst at midblastula transition

**DOI:** 10.1371/journal.pgen.1006757

**Published:** 2017-05-12

**Authors:** Gabriele Wagner, Nishant Singhal, Dario Nicetto, Tobias Straub, Elisabeth Kremmer, Ralph A. W. Rupp

**Affiliations:** 1 Biomedizinisches Zentrum, Molekularbiologie, Ludwig Maximilians-Universität, Großhaderner Strasse 9, Planegg Martinsried, Deutschland; 2 Bioinformatics Unit, Biomedical Center, Ludwig-Maximilians University, Großhaderner Strasse 9, Planegg Martinsried, Deutschland; 3 Core Unit Monoclonal Antibodies, Helmholtz Zentrum München, Marchioninistrasse 25, München, Deutschland; University of Illinois at Urbana-Champaign, UNITED STATES

## Abstract

Zygotic gene expression programs control cell differentiation in vertebrate development. In *Xenopus*, these programs are initiated by local induction of regulatory genes through maternal signaling activities in the wake of zygotic genome activation (ZGA) at the midblastula transition (MBT). These programs lay down the vertebrate body plan through gastrulation and neurulation, and are accompanied by massive changes in chromatin structure, which increasingly constrain cellular plasticity. Here we report on developmental functions for Brahma related gene 1 (Brg1), a key component of embyronic SWI/SNF chromatin remodeling complexes. Carefully controlled, global Brg1 protein depletion in *X*. *tropicalis* and *X*. *laevis* causes embryonic lethality or developmental arrest from gastrulation on. Transcriptome analysis at late blastula, before development becomes arrested, indicates predominantly a role for Brg1 in transcriptional activation of a limited set of genes involved in pattern specification processes and nervous system development. Mosaic analysis by targeted microinjection defines Brg1 as an essential amplifier of gene expression in dorsal (BCNE/Nieuwkoop Center) and ventral (BMP/Vent) signaling centers. Moreover, Brg1 is required and sufficient for initiating axial patterning in cooperation with maternal Wnt signaling. In search for a common denominator of Brg1 impact on development, we have quantitatively filtered global mRNA fluctuations at MBT. The results indicate that Brg1 is predominantly required for genes with the highest burst of transcriptional activity. Since this group contains many key developmental regulators, we propose Brg1 to be responsible for raising their expression above threshold levels in preparation for embryonic patterning.

## Author summary

Brahma-related-gene-1 (Brg1) is a catalytic subunit of mammalian SWI/SNF chromatin remodeling complexes. Loss of maternal Brg1 protein arrests development in mice at the 2-cell stage, while null homozygotes die at the blastocyst stage. These early requirements have precluded any analysis of Brg1’s embryonic functions. Here we present data from *X*. *laevis* and *X*. *tropicalis*, which for the first time describe a role for Brg1 during germ layer patterning and axis formation. Brg1-depleted embryos fail to develop past gastrulation. Genome-wide transcriptome analysis at late blastula stage, before the developmental arrest, shows that Brg1 is required predominantly for transcriptional activation of a limited set of genes involved in pattern specification processes and nervous system development shortly after midblastula transition. Mosaic analysis by targeted microinjection defines Brg1 as an essential amplifier of gene expression in dorsal (BCNE and Nieuwkoop center) and ventral (BMP/Vent) signaling centers, being required and sufficient to initiate axial patterning by cooperating with canonical Wnt signaling. Since Brg1-dependent genes share a high burst of transcriptional activation before gastrulation, we propose a systemic role for Brg1 as transcriptional amplifier, which balances the embryonic patterning process.

## Introduction

Vertebrate BAF protein complexes remodel chromatin with the mutually exclusive help of Brahma (brm) or Brahma-related gene 1 (brg1) ATPase subunits. SWI/SNF complexes are known to participate broadly in nucleosome-based aspects of DNA metabolism in normal and malignant cells [1–4], but their specific ATPase subunits designate them for different functions. *Brm*^-^/^-^ mice are viable although heavier than normal, suggesting Brm to be a negative regulator of cell proliferation [5]. In contrast, *brg1*^-/-^ mice die during early embryogenesis and brg1 heterozygotes are predisposed to exencephaly and tumor formation [6]. These results suggest unique functions for BAF complexes carrying the different ATPases. Brg1 containing BAF complexes become further subspecialized in a tissue specific manner by association with co-factors of the BAF60 protein family during cell differentiation [7, 8]. Specific functions have also been described in murine embryonic stem cells, where a specialized esBAF complex containing Brg1, Baf155 and Baf60a regulates aspects of ES self renewal, pluripotency and cell priming for differentiation [9–11].

How these findings for esBAF relate to normal mouse embryogenesis is not fully clear. Embryos lacking maternal Brg1 protein arrest at two cell stage and are compromised in zygotic genome activation [12], while embryos, lacking only zygotic Brg1 protein, die before implantation [6]. Since ATP dependent chromatin remodelers, including Brg1, are conserved among vertebrates [10, 13] deeper insight into Brg1’s developmental functions could be derived from non-mammalian vertebrate model organisms. Although Brg1 is expressed throughout development, several reports from *Xenopus* and Zebrafish have shown only relatively late requirement of Brg1 in development, i.e. during differentiation of heart, neural plate and brain [13–15]. We had obtained precedence for specific involvement of chromatin remodelers in developmental processes as early as germ layer formation in *Xenopus*, where Mi2-beta/NuRD remodeling activity is needed to position the boundary between mesoderm and neuroectoderm [13]. These findings let us expect also earlier functions for BRG1/BAF complexes. In search for such early functions, we have investigated the transcriptional and embryonic consequences of Brg1 depletion in the closely related species *X*. *tropicalis* and *X*. *laevis*.

## Results

### Consequences of global Brg1 protein knockdown

**Fig 1:**
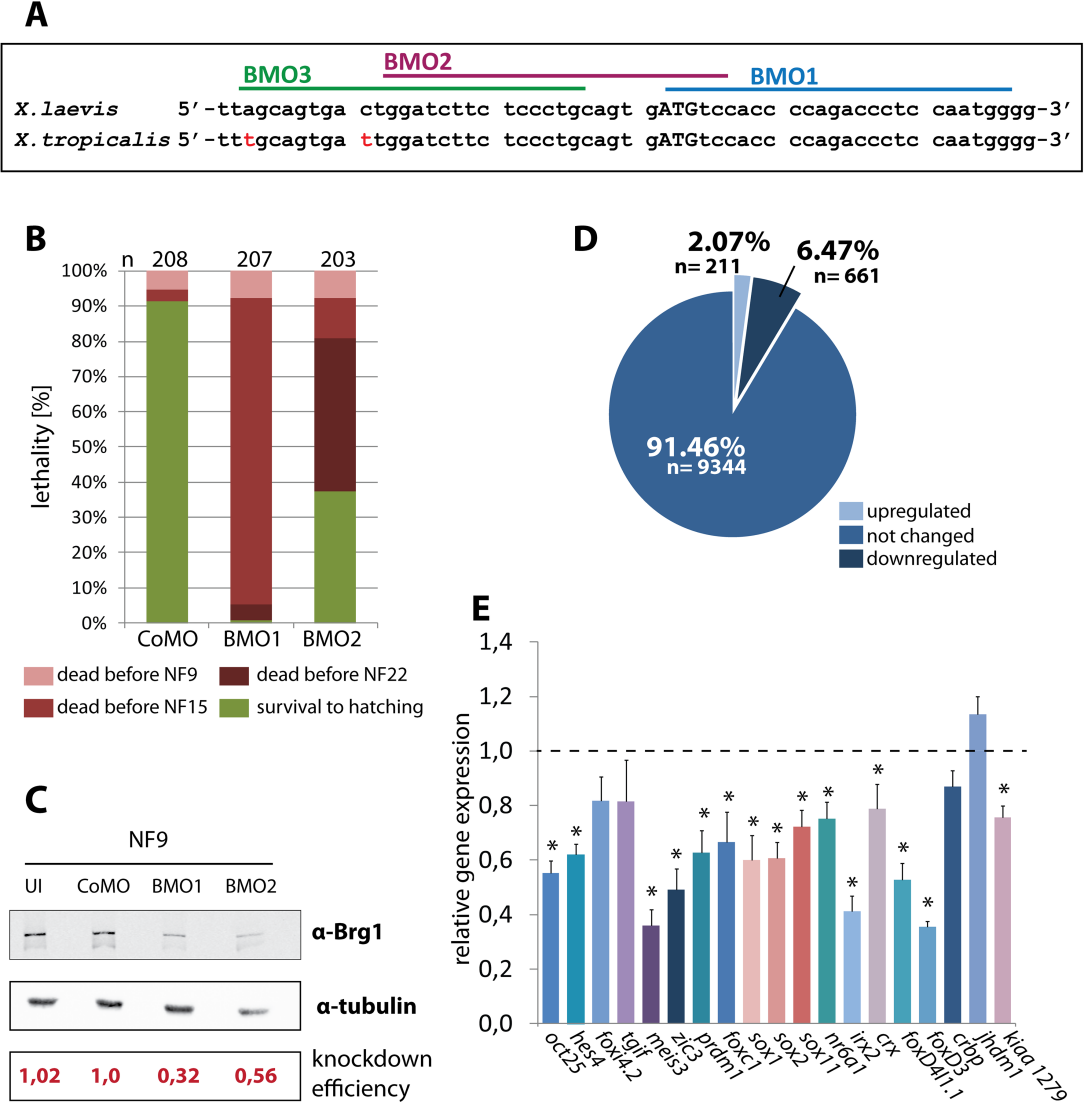
Normal Brg1 protein levels are essential for blastula gene expression and survival. **(A)** Target sites of antisense Morpholinos on *Xenopus* Brg1 mRNAs. Start codon in capital letters; sequence mismatches between *Xenopus* species are labeled in red. (**B)** Radial injections of BMO1 (30ng/embryo) and BMO2 (60ng/embryo) in *X*. *tropicalis* embryos cause embryonic death from gastrulation stages on (n = 8 biological experiments), while CoMO (60ng/embryo) injected embryos survived beyond hatching stage. Please note that the majority of BMO2 injected embryos die during neurulation. The death counts were recorded at the indicated color-coded stages. NF9: late blastula; NF15: neural fold stage; NF22: late neurula; NF36: hatching. **(C)** Western Blot analysis of unmanipulated or radially *injected X*. *tropicalis* embryos at late blastula stage (NF9). Brg1 protein levels were quantified relative to α-tubulin and normalized to CoMO-injections. BMO1 injected embryos had a mean Brg1 protein level of 0.48, while BMO2 injected embryos had a mean Brg1 protein level of 0.6 (n = 8 experiments). **(D)** Pie-diagram of the microarray analysis comparing CoMO and BMO1 morphant *X*. *tropicalis* at NF9 (averaged from four independent experiments). At this stage, 10216 mRNAs classified the active gene pool; 91.4% of these were unaffected, 6.5% were down- and 2.1% were upregulated by Brg1 protein knockdown (lfdr > 0.2). **(E)** Validation of the responders from the GO-term “nervous system development” by qRT/PCR (n = 8 independent experiments). Asterisks mark genes, which were significantly downregulated in the qRT/PCR analysis (p-value < 0.05).

To generate a Brg1 loss of function situation in Xenopus, we designed three Morpholino (MO) oligonucleotides against mRNAs of both *X*. *laevis* brg1 homoeologs (Fig 1A). We determined their relative translation blocking activities in *X*. *laevis* embryos with a recombinant *brg1/luciferase* transcript, containing ~700bp of the *brg1* cDNA sequences with the morpholino targeting regions fused in frame to the luciferase ORF. The blocking efficiencies of the Morpholinos increased about three-fold from 5’ to 3’ direction on the target mRNA. BMO1 had the strongest effect and reduced luciferase activity approximately seven-fold (S1 Fig). Based on these results, we selected BMO1 and BMO2 for further analysis.

We investigated the consequences of systemic Brg1 protein knockdown in *X*. *tropicalis* embryos, where the target region for BMO1 and BMO2 is conserved (Fig 1A). To achieve a homogenous protein knockdown, Morpholinos were injected four times into the animal pole region at the two- to four-cell stage (“radial” injection type). In titration experiments, we determined a dose of 30 ng BMO1/embryo to reduce Brg1 protein levels to one-third, while even 60ng of BMO2 reduced them only two-fold (Fig 1C). Whereas more than 90% of the control morphants survived until hatching, the majority of the BMO1 injected embryos died during gastrulation (Fig 1B). Consistent with less efficient Brg1 depletion, BMO2 morphant embryos died later than BMO1 morphants and survived better (Fig 1B). The survival rate of CoMO and BMO1 morphants was comparable until late blastula, although the Brg1 protein levels were already diminished in the latter case to about 30% of CoMO injected embryos (Fig 1C). The residual BRG1 protein is very likely of maternal origin [14] and therefore insensitive to MO knockdown. Notably, immunostaining for activated Caspase-3 showed no signs of apoptosis at early gastrula stage indicating that morphant blastulae are healthy and initiate gastrulation without visible defects or delay (S2 Fig).

Bulk zygotic transcription commences in *Xenopus* at the midblastula transition (MBT), about three hours before gastrulation starts. Because under our conditions BMO1 morphants die mostly during gastrulation or neurulation, but not before, it was possible to assess the consequences of Brg1 protein knockdown on embryonic transcription at late blastula. Using the same conditions described above, we compared CoMO and BMO1 injected *X*. *tropicalis* embryos by microarray analysis. Although these conditions were ultimately lethal, at the investigated late blastula stage more than 90% of the mRNAs were expressed at normal levels. A total of 872 transcripts responded to the Brg1 protein knockdown, with 211 of them being upregulated, and 661 being downregulated, relative to control morphants (Fig 1D, S1 and S2 Tables). Gene Ontology analysis revealed an enrichment for the terms “chromatin assembly”, “cellular complex assembly”and “macromolecular complex assembly” in the upregulated cohort (S3 Fig, panel A). In contrast, the downregulated responders were strongly enriched in several GO terms related to various developmental and pattern specification processes (S3 Fig, panel B). Here the most enriched term was “nervous system development”, consistent with a known requirement for Brg1 during vertebrate neural differentiation [14–17]. Nineteen of the 61 genes from this GO category were reduced in our genome-wide data set already at the blastula stage, i.e. before neural plate formation. From these 19 genes, fifteen were found reduced by independent qRT/PCR analysis (Fig 1E). In addition we reproduced the microarray results for a variety of important developmental regulatory genes by qRT/PCR (S3 Fig, panels C and D). Furthermore, we investigated by whole-mount RNA in situ hybridization (WMISH) the expression patterns of genes involved in neural induction at late blastula, confirming the microarray results for downregulated *foxD4l1* and *noggin* expression, and unaffected *zic2* expression in BMO1 morphants (S3 Fig, panels E-K’). These independent analyses confirmed the microarray data in a robust manner. In summary, our results indicate an essential function for Brg1 protein before the onset of gastrulation, detailing primarily an enhancement of gene transcription.

### Local Brg1 protein depletion strongly impairs dorso-anterior differentiation in *X*. *laevis*

We repeated the morphological analysis in *X*. *laevis* by injecting radially the BMO1 morpholino at 60 ng, 40 ng and 20ng per embryo, together with a fluorescent lineage tracer (S4 Fig). At the two higher doses, BMO1 injections caused again embryonic death in the majority of the embryos before late neural tube stage (NF22; S4 Fig panel C). All remaining embryos were arrested in gastrulation (S4 Fig, panel A), occasionally surviving in this state until the heartbeat stage (NF34; S4 Fig, panel B). At a dose of 20 ng, more than half of the embryos survived until NF22; while about 80% of the survivors did not finish gastrulation, 20% became arrested at the open neural plate stage and remained in this condition until NF34 (S4 Fig, panels B and C). In summary, embryonic survival is correlated with the BMO1 dose. Moreover, the formation of dorso-anterior structures is still completely blocked under conditions (20ng BMO1/radial), in which the majority of the embryos survives beyond neurulation. Therefore, we wondered, whether local ablation of Brg1 protein by a reduced Morpholino dose might improve overall embryonic differentiation and thus provide information on developmental pathways requiring Brg1 activity. We tested this hypothesis in *X*. *laevis* embryos by targeted microinjections.

**Fig 2:**
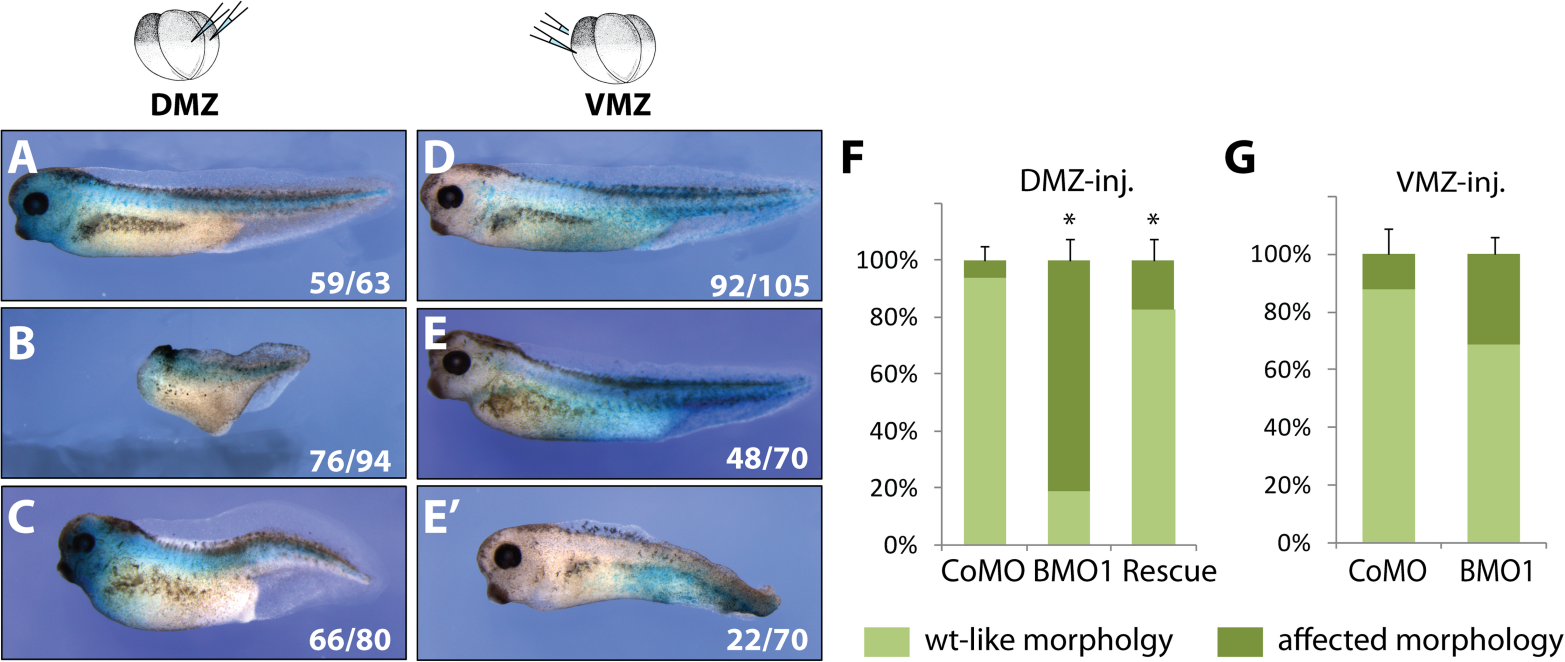
Targeted knockdown of Brg1 protein leads to morphological defects. Dorsal marginal zone (DMZ) injections in *X*. *laevis*: **(A)** CoMO (10ng) injections have no effect on development. **(B)** BMO1 (10ng) injections cause stunted axes with severely reduced dorso-anterior tissues. **(C)** Coinjection of BMO1 and hBrg1 mRNA (1ng) restores AP-axes and anterior tissues like eyes, (n = 3 experiments). Ventral marginal zone (VMZ) injections: **(D)** CoMO (10ng) injection with no phenotype. **(E)** The majority of BMO1 (10ng) injected embryos appear wildtype-like. **(E’)** A smaller fraction of BMO1 morphants (10ng) show enlarged heads and eyes paired with deficits in posterior and ventral tissues. All embryos were coinjected with *nlacZ* mRNA as lineage tracer. **(F)** and **(G)** Phenotypic penetrance of DMZ/VMZ injected embryos (n = 3–5 experiments; *, p-value ≤ 0.03).

When CoMO or BMO1 oligonucleotides (10ng/embryo) were injected at the 4-cell stage into the marginal zone of either the two dorsal (DMZ), or the two ventral (VMZ) blastomeres, most of these embryos survived until tadpole stage. β-Galactosidase staining for coinjected *nlacZ* mRNA confirmed the correct targeting of the injections and demonstrated viability of the injected cell progeny. DMZ injected control morphants grew up into phenotypically wildtype tadpoles (Fig 2A). In contrast, 80% of DMZ injected BMO1 morphants displayed stunted antero-posterior body axes with severely truncated heads carrying the *nlacZ* stain (Fig 2B and 2F). The remaining 20% of BMO1 morphants showed no morphological abnormalities. The major phenotype was a specific consequence of Brg1 protein depletion, since dorso-anterior structures were largely rescued by coinjection of wildtype human *brg1* mRNA that contains four mismatches in the BMO1 target region. The heads of these rescued embryos contained well-developed eyes with lenses and recurrent retinal pigment (Fig 2C). Interestingly, this rescue of the BMO1 morphant phenotype was neither achieved with human *brm* nor *Xenopus iswi* mRNAs (S3 Table). When we inspected VMZ injected embryos, their overall morphology was much less affected. Although we have not investigated any internal organs, these embryos were at least capable to develop a well-structured body axis including heads (Fig 2D, 2E and 2G). A smaller fraction (30%) of the BMO1 morphants was weakly anteriorized, displaying enlarged heads and eyes, but concomitantly deficient in posterior tissues such as the fin (Fig 2E’ and 2G).

Several conclusions can be drawn from these results. First, the presence of β-Gal positive cells in tadpoles demonstrates that BMO1 injections are not cell-lethal per se. Second the inability of Brm and Iswi to rescue the BMO1 phenotype argues for distinct remodeling events that specifically require Brg1 protein. Finally, the observed morphological phenotypes suggest that Brg1 is involved in axis formation. Particularly on the dorsal side of the embryo, Brg1 protein seems required to unfold the dorsalizing gene expression program (DGEP) during germ layer patterning.

This assumption was investigated by several experiments. First, we coinjected *Xenopus brg1* and *nlacZ* mRNAs into one ventral blastomere at the 4-cell stage of wildtype embryos. At the tadpole stage, these embryos had formed with high penetrance a secondary axis rudiment, which contained somites with differentiated muscle tissue (S5 Fig). Moreover, the chordin gene is one of the early developmental regulators, downregulated in radial *X*. *tropicalis* BMO1 morphants (S3 Fig, panel C, S2 Table). This gene was ectopically induced by ventral injections of human *brg1* mRNA (S6 Fig). Notably, hBrg1 also efficiently restore dorso-anterior development in DMZ injected BMO1 morphants (Fig 2C and 2F). These two observations indicate that Brg1 protein overexpression can initiate *de novo* formation of axial structures, apparently through activation of DGEP genes like *chordin*.

**Fig 3:**
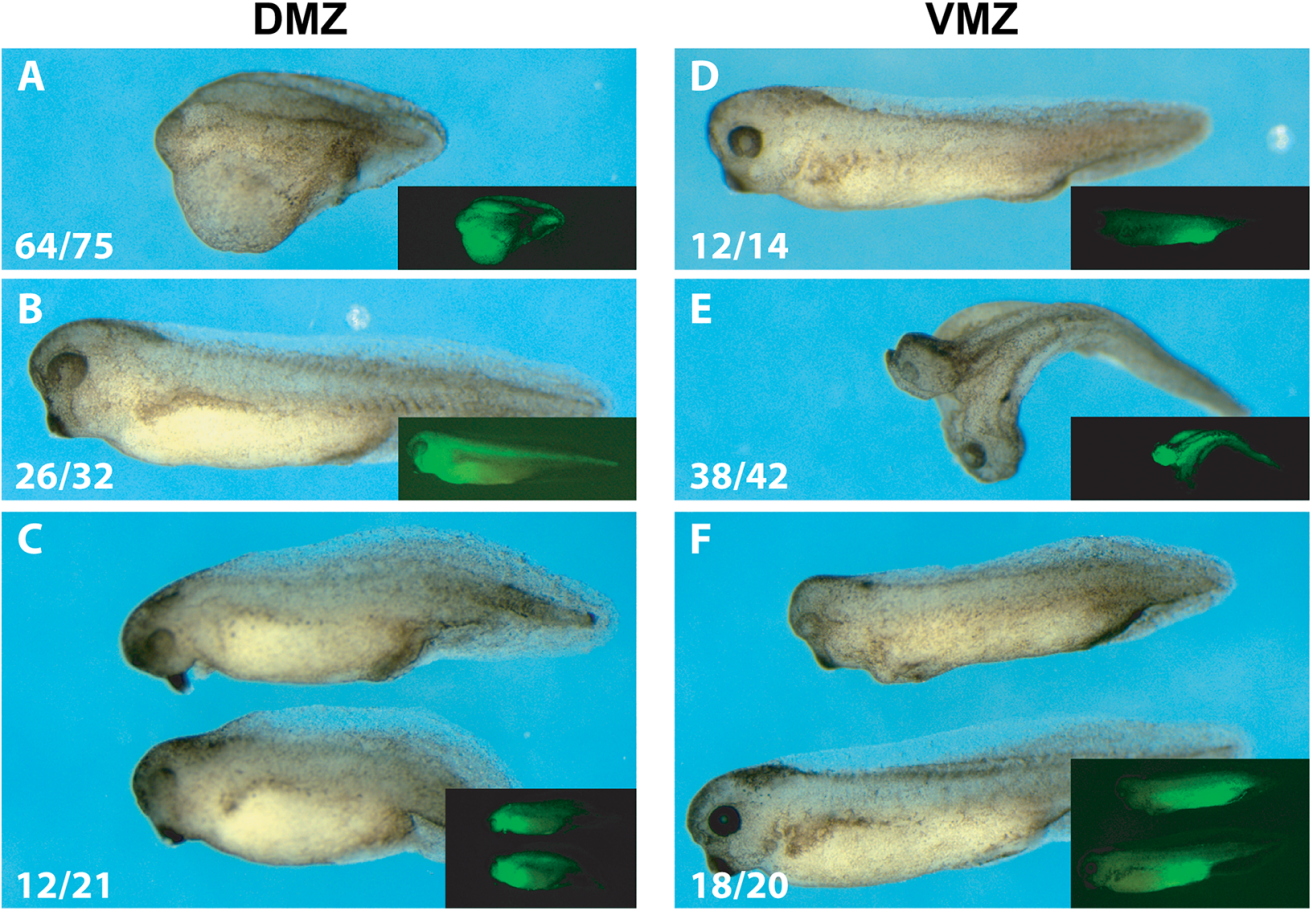
Brg1 cooperates with maternal Wnt signaling for axis formation in *X*. *laevis*. Panels **(A-C)** axial rescue of DMZ-injected BMO1 morphants (10ng) by coinjected *ß-catenin* mRNA (250pg). **(A)** DMZ-injected BMO1 morphant with severly impaired antero-posterior axis (n = 64/75 “headless”); **(B)** overexpression of *ß-catenin* in DMZ does not cause gross abnormalities (n = 26/32 wt-like); **(C)** coinjection of BMO1 and *ß-catenin* rescues the truncated 1° axis (n = 12/21 heads with eyes and cement gland). Panels **(E-G)** show that *de novo* induction of a second embryonic axis by *ß-Catenin* (250pg) depends on endogenous Brg1 activity. **(E)** VMZ-injected BMO1 morphants (10ng) display wt-like morphology (n = 12/14; see also Fig 2D); **(F)** VMZ-injection of 250pg β*-catenin* mRNA induce a second body axis (n = 38/42 complete secondary heads with eyes and cement gland); **(G)** Coinjection of BMO1 with β*-catenin* mRNA blocked almost completely the induction of secondary axes (n = 18/20 single axis status; 1 double axis remaining). Correct targeting of injections was verified by fluorescence from coinjected GFP mRNA (100pg; see inserted images).

Normally, formation of dorsal structures is initiated by maternal Wnt/β-Catenin signaling on the prospective dorsal side of the embryo [18–20]. Coinjection of β*-catenin* mRNA at a dose, which alone was insufficient to cause morphological consequences, reestablished quite efficiently head structures, including eyes, as well as longer body axes and tails in DMZ-injected BMO1 morphants (Fig 3A–3D). When injected ventrally, β-Catenin frequently induced a secondary embryonic anlage with complete heads, which was reduced to single-axis status by coinjection of BMO1 (Fig 3E–3H). These last experiments demonstrate a cooperation between canonical Wnt signaling and Brg1 in early embryonic patterning, which had not been observed before.

### Brg1 is required for gene expression in the BCNE

**Fig 4:**
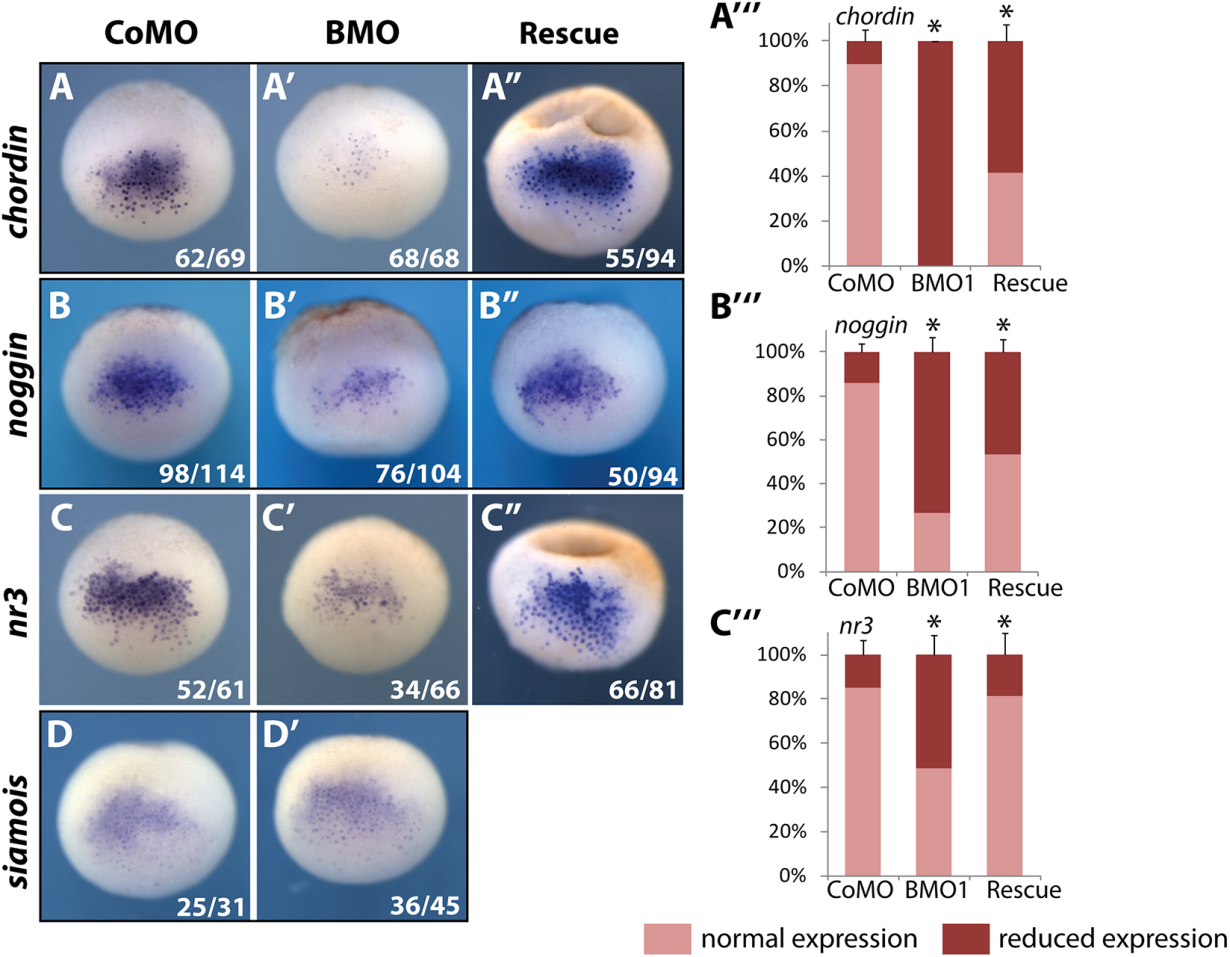
Brg1 protein knockdown diminishes gene expression in the BCNE center. Pictures show dorsal views of whole mount RNA in situ hybridization (WMISH) at late blastulae (NF9, *X*. *laevis*) with gene expression patterns representative for the experimental condition. Injection conditions are indicated on top: CoMO (60ng/embryo), BMO1 (60ng/embryo), Rescue (BMO1 plus hBRG1 mRNA [1ng/embryo]). Reagents were radially injected into the animal hemisphere. The mRNA expression patterns of *chordin*, *noggin* and *nodal-related 3 (nr3)* in control morphants (panels **A**-**C**) were reduced in BMO1 morphants (panels **A’**–**C’**), but restored by coinjection of human *brg1* mRNA (panels **A”** –**C”**). The graphs (**A”‘**–**C”‘**) depict phenotypic penetrance (n = 3–4 experiments/condition). The expression of *siamois* was not affected by BMO1 (panels **D** + **D’**, n = 2 experiments/gene).

At blastula stage, DGEP is initiated by two newly induced, local signaling centers–the dorso-animal Blastula-Chordin-Noggin-Expressing (BCNE) Center and the overlapping, but more vegetally located Nieuwkoop Center (NC). Both regions contribute to head-formation and are induced by maternal WNT-signaling [21]. The BCNE signature genes, including *nodal3*.*1/nr3*, *chordin* and *noggin*, encode secreted BMP inhibitors. All these genes, in particular *chordin*, were downregulated by radial Brg1 protein knockdown in *X*. *laevis* (Fig 4, panels A-C). Expression of the BCNE genes was largely restored by coinjection of human *Brg1* mRNA (Fig 4A”–4C”), consistent with the previously described morphological rescue of dorso-anterior tissues (see Fig 2). The downregulation of *chordin* and *noggin* mRNAs matches our results in *X*. *tropicalis* (S3 Fig, panels C, and G to H’). We note that the expression of *nr3* was upregulated in *X*. *tropicalis* blastulae (S3C Fig), while it is downregulated in the *X*. *laevis* BCNE. Possible mechanisms for this species-specific difference are discussed later.

The NC marker gene *sia1* is a direct Wnt-target, a key regulator of axial patterning, and is needed for proper gene expression in BCNE and Spemann’s organizer [22–24]. Notably, expression of *sia* mRNA in radial BMO1 morphants was not downregulated compared to control morphants (Fig 4D and 4D’), consistent with the results for sia1 and sia2 from the microarray analysis in *X*. *tropicalis* (S3C Fig). The insensitivity of *sia1*/*sia2* genes excludes a role of Brg1 protein as general coactivator of Wnt/β-Catenin signaling in *Xenopus*, which had been suggested by earlier studies [25]. Notably, Brg1 is involved in both direct (*nr3*; [26]) and indirect [*chd*, *nog*; ref.[27]] Wnt-mediated gene activation events in the BCNE, which is consistent with the morphological phenotypes observed in DMZ-injected BMO1 morphants.

### Brg1 protein knockdown impacts the organizer and dorso-ventral patterning

The process of germ layer patterning, which defines the future body plan, occurs during gastrulation and requires Spemann’s organizer, which overlaps with and succeeds the BCNE and NC territories. Cells of the organizer are the first ones to involute during gastrulation. They secrete a panoply of proteins, which inhibit BMP as well as Wnt and Nodal signaling pathways [28–30]. These organizer properties generate gradients of signaling activities that dynamically establish gene expression domains of appropriate size within the morphogenetic field of the forming germ layers [31, 32]. Since we had discovered that BCNE gene expression depends on normal BRG1 protein levels, we sought to extend the analysis to gene expression domains of organizer and non-organizer mesoderm.

**Fig 5:**
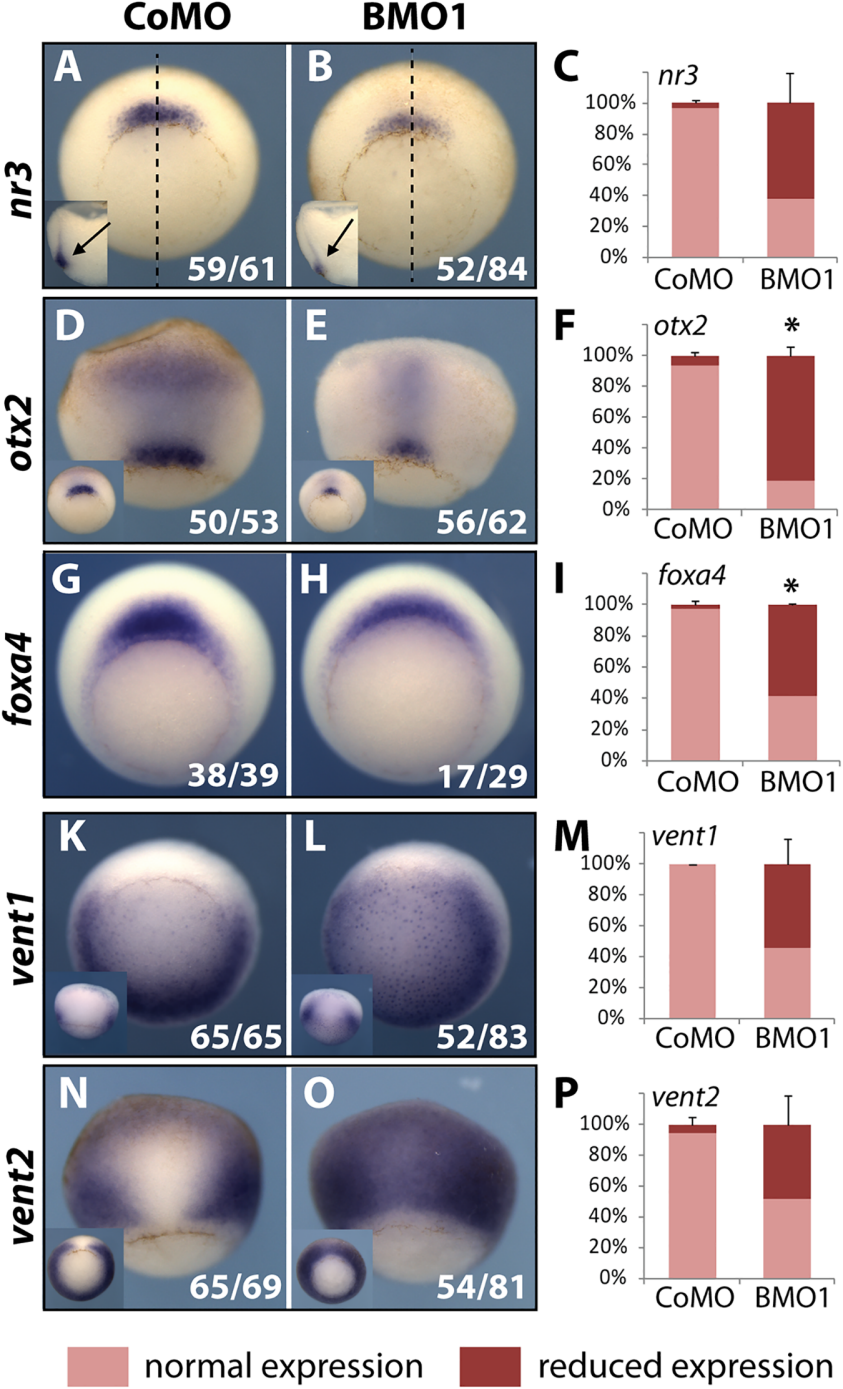
Reduced Brg1 protein levels impair organizer function. *X*. *laevis* embryos were radially injected with 40ng of indicated MOs and fixed at early. Representative images of the predominant morphant expression patterns are shown by WMISH in vegetal (**A**, **B**, **G**-**L**) or dorsal (**D**, **E**, **N**, **O**) side views. The Organizer region is represented by *nr3* (**A-C**), *otx2* (**D-F**) and *foxA4* (**G-I**). Inserts depict deep staining in bisected embryos for *nr3* or blastopore lip staining for *otx2*. Non-organizer mesoderm is represented by the BMP target genes *vent1* (**K M**) and *vent2* (**N-P**). Inserts show side views for *vent1* and vegetal views for *vent2* (n = 2-4 independent experiments/gene).

We evaluated the expression of critical regulatory factors by WMISH in early to mid gastrula stage (NF10.5 to NF11), i.e. before development becomes typically arrested in *X*. *laevis* BMO1 morphants (S4 Fig). Among the organizer genes was the BMP inhibitor *nr3* [33], which at blastula stage was downregulated in the BCNE. The *nr3* mRNA levels were reduced in most gastrulae within its normal domain (Fig 5A–5C). The *otx2* gene is first transcribed in the organizer and specifies at later stages anterior tissues in all three germ layers [34]. Upon Brg1 knockdown, the intensity of *otx2* staining and the size of its domain were reduced. This was true both for preinvoluted *otx2* expression at the blastopore lip as well as for the involuted part, where *otx2* mRNA is confined to a narrow stripe in BMO1 morphants (Fig 5D–5F). Finally, *foxA4* mRNA was frequently downregulated in its proper domain at the lip (Fig 5G–5I). Some non-organizer genes in the neighboring dorso-lateral mesoderm were also misregulated. This included the homeobox genes *vent1* and *vent2*, which mediate the ventro-posteriorizing activity of BMP ligands [35]. In BMO1 morphants, transcripts from both genes invaded the organizer territory (Fig 5K–5P). This dorsal expansion of *vent* gene expression indicates a severe functional impairment of Spemann’s organizer [36]. In addition, the muscle regulatory genes *myoD* and *myf5* were reduced (S7 Fig, panels A-F), while *gsc*, *t/bra* and *xpo* were unaffected (S7 Fig, panels G-S). Also chordin transcription was unimpaired in the organizer, despite the fact that it is downregulated in the BCNE region at blastula stage (compare S7K–S7M Fig with Fig 4A). While *chordin* is activated by maternal Wnt signaling in the BCNE, it is controlled in the organizer by additional regulators including nodal signaling and the mesodermal transcription factors *gsc* and *not* [37, 38]. These findings suggest a context-dependent role for Brg1 in target gene regulation. In summary, a systemic depletion of BRG1 protein leads to a misbalance in dorso-ventral patterning and an unusual coexistence of non-organizer (*vent1/vent2*) and organizer transcripts within the dorsal blastopore lip.

### Brg1 depletion from BCNE causes autonomous defects in brain differentiation

**Fig 6:**
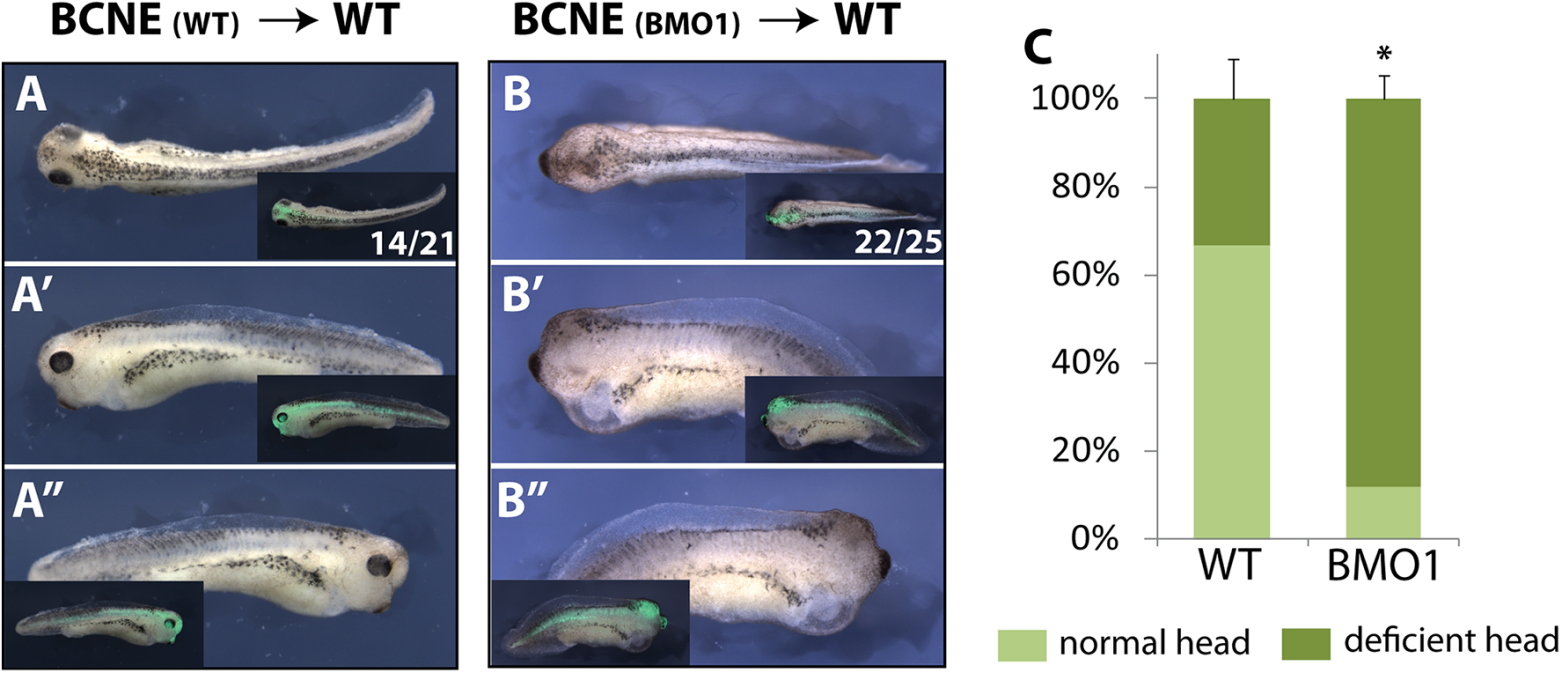
Brg1 is required in the neuroectoderm for proper eye and brain formation. **(A-C)** Orthotopic transplantations in *X*. *laevis* embryos. For experimental scheme see S8 Fig, panel A. Shown are dorsal, left and right views of single transplanted embryos at tadpole stage. **(A-A”)** control transplanted embryo. **(B-B”)** BMO1 morphant BCNE transplanted embryo. In **(C)** quantification of the transplantation results (n = 5 experiments).

Our morphological and molecular analyses defined the earliest defects in BRG1-depleted embryos to the late blastula stage, when the BCNE center is established. Kuroda and colleagues have demonstrated by tissue transplantation that the BCNE contributes to brain formation [21]. Therefore, we decided to address by orthotopic transplantation, whether the absence of eyes and forebrains in BMO1 morphants arises autonomously from the BCNE region, or results from a defective crosstalk between germ layers. For this purpose we transplanted wildtype or morphant BCNE grafts into wildtype host embryos (Fig 6). Grafts were marked by fluorescent dextran to distinguish them from host tissues (experimental workflow see S8A Fig). At the tadpole stage, two-thirds of the embryos transplanted with a WT-BCNE had generated tadpoles with heads containing well-developed eyes with lenses (n = 14/21; Fig 6A–6A”). In contrast, almost 90% of the embryos transplanted with a morphant BCNE lacked eyes completely or had only remnants of retinal pigmentation without lenses (n = 22/25; Fig 6B–6B”). The morphological differences between the two conditions were significant (Fig 6C). To visualize the major brain domains we stained the transplanted tadpoles for *otx2* mRNA (S8 Fig, panels B-E). Half of the morphant transplants showed a strong reduction in *otx2* expression, in which forebrain, midbrain and hindbrain areas were collapsed to an amorphous tissue mass. This result was particularly obvious in specimen, in which the lineage tracer of the transplanted BCNE populated only part of the brain. In these cases, *otx2* staining was structured comparatively normal in the host-derived parts of the brain, while it was severely reduced in the transplanted area (S8 Fig, compare panels C, D with C’ and D’). The clearest results were observed for *otx2* expression in the retina, which was present in 90% of the WT transplants, but only in 25% of the morphant transplants. In summary the results from this experimental series indicate an autonomous defect within the Brg1-depleted BCNE region for neuronal differentiation and brain patterning, which cannot be compensated by secreted factors from the wildtype host environment, including mesodermal Chordin (S7K–S7M Fig).

### Genes outside the BCNE contribute to the BMO1 morphant phenotype

**Fig 7:**
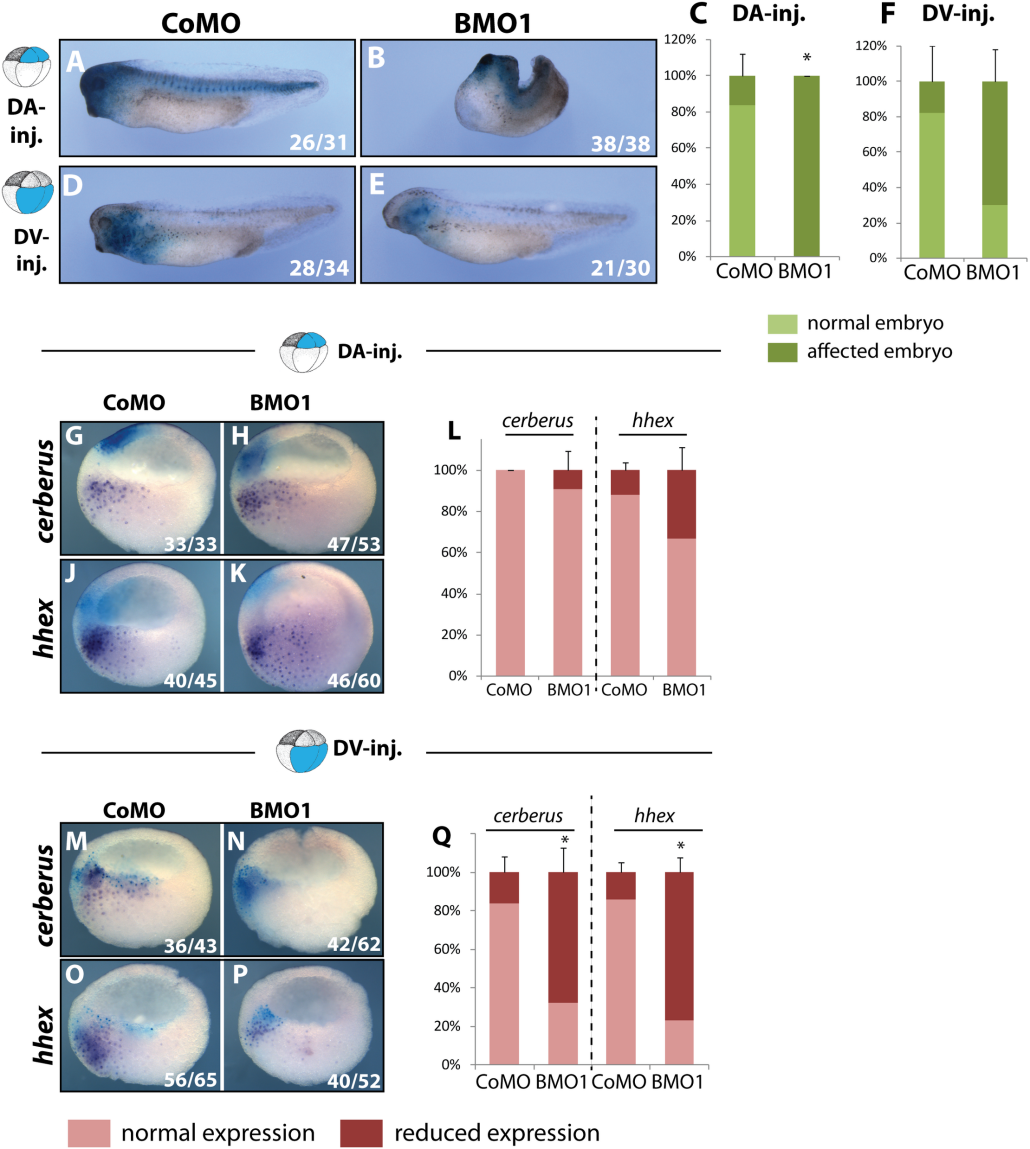
Downregulation of Nieuwkoop Center genes contributes to the dorso-anterior phenotype in Brg1 morphants. At eight cell stage *X*. *laevis* embryos were injected either in the two dorso-animal blastomeres (DA) or the two dorso-vegetal blastomeres (DV) with 10 ng of the indicated MO-oligo. *Nuclear LacZ* mRNA was coinjected as lineage tracer. Embryos were cultivated until hatching stage **(A-F)** for morphological assessment or until late blastula/early gastrula stage **(G-Q)** for WMISH analysis. In sagittally bisected embryos the two Nieuwkoop marker genes *cerberus* **(G, H, M, N)** and *hhex* **(J, K, O, P)** were analysed after indicated injections at late blastula and early gastrula. As indicated by *nlacZ* staining, the expression domains and the injection domains do not overlap in DA-injected embryos. Quantification of DA-injections **(L)** and DV-injections **(Q)** (n = 5–7 biological replicates). *, p-value ≤ 0.05.

We have demonstrated that several BCNE genes, in particular *chordin*, are specifically downregulated in BMO1 morphants and are responsible for defective head formation. However, the global transcriptome analysis had revealed a much larger number of genes responding to BRG1 knockdown, suggesting that other regions of the embryo also contribute to the BMO1 phenotype. In a new series of experiments, we compared side by side the consequences of dorso-animal (”DA”/BCNE center) with dorso-vegetal (“DV”/Nieuwkoop Center) blastomere injections at the eight-cell stage. The majority of control morphants developed completely normal in both types of injections (Fig 7, panels A, D, C and F). As expected, DA-injections of BMO1 resulted in embryos with shorter, tailless axes, and strongly reduced heads (Fig 7, panels B and C). Targeting of the BMO1 to DV-blastomeres maintained the length of the main body axis much better than DA-injections, but still reduced head and eye formation in a large fraction of the embryos (Fig 7, panels E and F).

RNA *in situ* hybridisation and ß-Galactosidase staining at the late blastula stage confirmed that both DA and DV blastomeres contribute to the BCNE expression zone, as it has been described before [39, 40]. Consequently, *chordin* and *noggin* mRNAs were downregulated with both injections within the overlap (S9 Fig, panels A-J). The genes *hhex* and *cer1* are expressed in the dorso-vegetal region and are known to promote anterior development and head formation, respectively [41, 42]. DA-injections of BMO1, which do not overlap with the *hhex* and *cer1* expression domains, had no effect on these mRNAs (Fig 7, panels G-L). In contrast, DV-injections strongly downregulated both genes in a statistically significant manner (Fig 7, panels M-Q). Most importantly, the results from targeted 8-cell injections demonstrate an additional role for Brg1 in the Nieuwkoop Center, the prospective anterior endoderm region of the embryo, where it is required for *hhex* and *cer1* transcription.

### Brg1 amplifies the transcriptional burst at the midblastula transition

The morphological and molecular analysis of its protein knockdown phenotype demonstrated Brg1 to be essential for embryonic vitality and germ layer patterning. Targeted injections of BMO1 oligo to different regions of the embryo support this conclusion in a consistent manner and revealed functional connections of Brg1 to several signaling pathways (Wnt/Bmp) and embryonic regions (BCNE/anterior mesendoderm). In search for a common denominator of Brg1 function, which could explain both the diverse impact on embryonic gene expression at late blastula stage (>800 altered transcripts) and the developmental functions in dorsal and ventral signaling centers, we investigated the gene response in Brg1 morphants in relation to the zygotic genome activation (ZGA) at MBT. Precedence for this assumption comes from work in mice, where absence of maternal BRG1 protein has been reported to cause developmental arrest at 2-cell stage and to impair transcription during ZGA [12].

**Fig 8:**
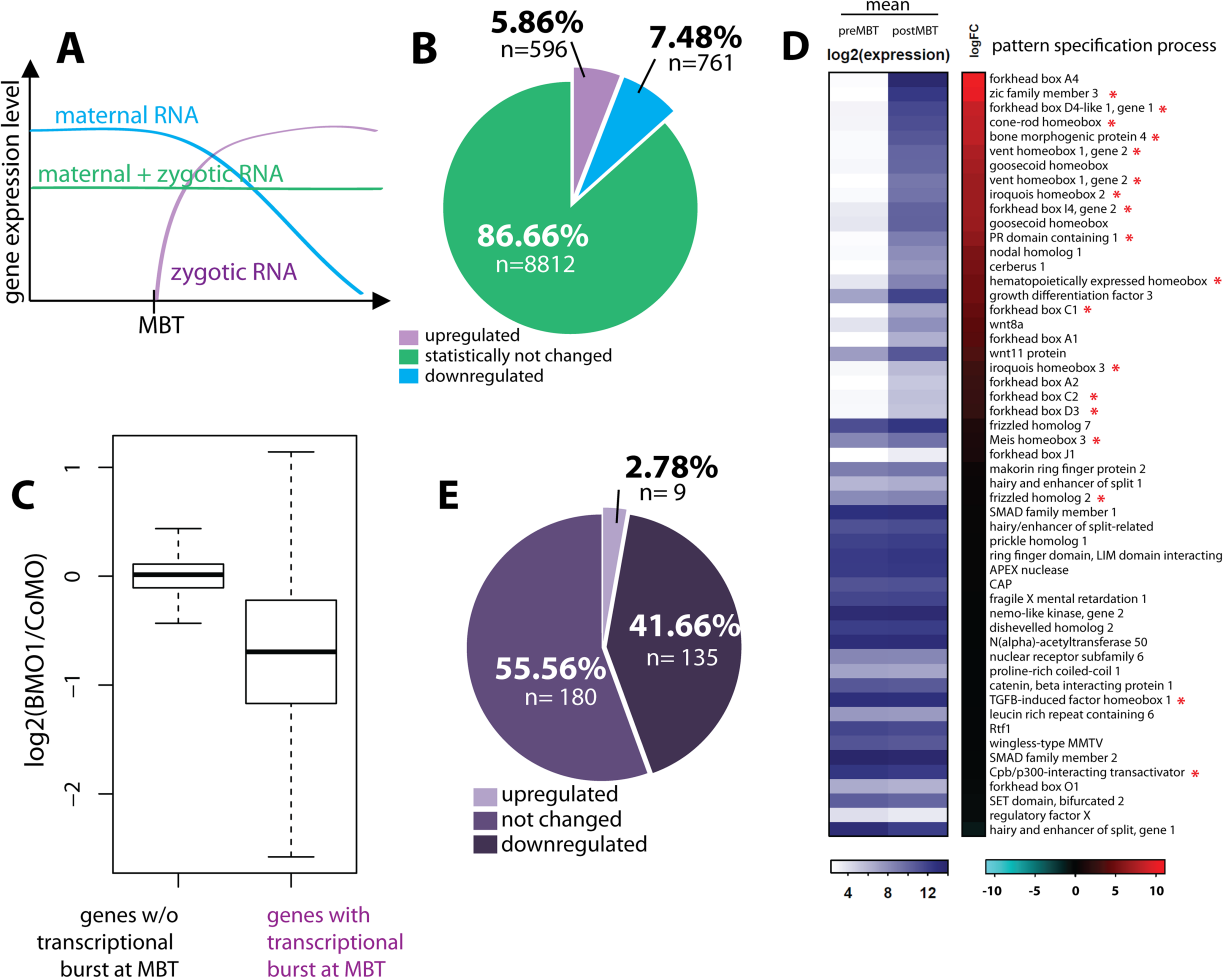
Brg1 amplifies gene transcriptional activation at the MBT. **(A)** Prototypic changes in mRNA pools during zygotic genome activation. (**B**) Global transcriptome analysis at pre-MBT and post-MBT timepoints from *X*. *tropicalis* embryos. (**C)** Box plot illustrating the response of RNA pools in BMO1 morphants versus control morphants. Note that overall zygotically activated genes are reduced upon Brg1 knockdown. (**D**) Heatmap providing mean expression levels at pre- and postMBT timepoints for genes of the GO-term “pattern specification process”, ranked by magnitude of activation (log2-fold change). Red asterisk mark genes that were more than 1.5-fold downregulated in BMO1 morphant embryos compared to control morphant siblings. (**E**) Pie Diagram for the “highly” upregulated at MBT” gene class (mRNA increases more than 5.65 fold; n = 324 genes). In response to Brg1 protein knockdown, nearly 42% of them (n = 135) were not activated to their full amplitude.

While originally identified as global onset of zygotic transcription, MBT is now recognized as a continuous reorganization of the embryonic mRNA pool from early to late blastula stages, consisting of a major turnover of maternal mRNAs, coupled to broad, but not genome-wide initiation of transcription [43–45]. As shown in Fig 8A, three prototypic transcript classes can be operationally defined—i) maternal mRNAs, whose abundance declines; ii) transcripts with relatively constant abundance, and iii) mRNAs with increasing abundance through *de novo* transcription. We decided to characterize the three mRNA classes through global transcriptome analysis at immediate pre-MBT (NF8) and late Blastula stages (NF9) in *X*. *tropicalis* embryos (see S10 Fig, panels A and B for details). We then compared these data with the transcriptional changes observed in the BMO1 morphants at late blastula.

About 10^4^ genes are expressed at the two time points (Fig 8B). After setting a threshold for transcript levels with an adjusted p-value ≤0.05, the abundance of 1357 transcripts changes between pre-MBT and late blastula. Decreasing levels characterize 761 transcripts as maternal mRNAs, whereas 596 transcripts classify as zygotic mRNA due to their increasing abundance (Fig 8B and S4 Table). By plotting the difference in mRNA levels between Brg1 morphants versus control morphants, we found that transcripts from the 596 genes activated at the MBT responded much stronger to the Brg1 knockdown than other transcripts (Fig 8C). A Gene Ontology search associated these zygotic transcripts with “Regulation of Transcription”, “RNA Metabolic Process”, and “Pattern Specification Process” (S10 Fig, panel C). When genes of the GO-term “Pattern specification process” were ranked in a heat map according to their magnitude of transcriptional activation, many of them were significantly downregulated by Brg1 protein knockdown (red asterisks, Fig 8D). A similar correlation between Brg1 dependence and transcriptional activation at MBT was found for the GO-term “Nervous System Development” (S10 Fig, panel D). In both cases, Brg1-dependent genes were enriched in the upper half of the heat maps, where genes with the highest fold-activation are located. The correlation was even more striking, when only the top-activated zygotic RNAs (≥5.65-fold increase after MBT; n = 324 genes) were considered—over 40% of these genes were Brg1 dependent (Fig 8E). Based on this analysis Brg1 protein is needed to amplify a transcriptional burst at MBT, which is necessary to initiate embryonic patterning.

## Discussion

To investigate potential developmental functions for Brg1 between MBT and neurula stages, we have carefully optimized the conditions for Brg1 protein depletion using three overlapping Morpholino oligonucleotides. These MOs differed significantly in their ability to block Brg1 protein synthesis, with the most upstream located targeting site of BMO3 having the least effect. The binding site of the Morpholino oligo used in earlier studies [46] starts 15 nucleotides upstream of BMO3 and does not overlap with BMO1/-2 target regions, presumably resulting in suboptimal targeting of *brg1* mRNAs. We consider this the most likely explanation for the much earlier and more severe effects we report in this study. The demonstrated reduction of endogenous Brg1 protein levels in combination with the robust rescue of both morphological and molecular aspects of BMO1 morphants by human *brg1* mRNA identifies the reported phenotype as a specific consequence of the Brg1 protein knockdown. Furthermore, both Brm and Iswi fail to compensate the Brg1 deficiency, suggesting specific remodeling events for Brg1-containing SWI/SNF complexes as the underlying molecular cause. These findings are in agreement with other reports [46–48].

Our results extend in a significant manner the current knowledge about the role of Brg1 during vertebrate embryogenesis. Transcriptome analysis from *X*. *tropicalis* late blastula stage (NF9) indicates that nearly 9% of transcripts (Fig 1D) were sensitive to Brg1 depletion. By comparison of the pre- and post-MBT transcriptomes we classified almost 600 transcripts to be *de novo* expressed at MBT, a number well in agreement with current estimates of *X*. *tropicalis* zygotic genome activation [47, 48]. The majority of these newly activated genes is Brg1-sensitive, detailing a significant impact for Brg1 on the first wave of zygotic gene expression of the embryo. In general we found that the gene response to Brg1 depletion is conserved between the two frog species. One clear exception is *nr3/nodal3*.*1*, which in the absence of Brg1 is downregulated in *X*. *laevis*, but upregulated in *X*. *tropicalis* (Figs 4, 5 and S2). Such a differential response could involve a species-specific modulation of the functional outcome of Brg1 activity on *nr3* gene transcription, and/or reflect differences in the transcriptional regulation of these orthologs. Indeed, there is evidence for chromosomal rearrangements and gene amplifications at the nr3/nodal3.1 gene locus [49] which may have changed the regulation of *nr3* transcription in the *X*. *laevis* L genome.

The short time span between MBT and late blastula (~3 hrs) implies that many, if not all misregulated genes are direct targets of Brg1-SWI/SNF. We expect this assumption to be valid for up- and downregulated gene cohorts alike, based on abundant evidence implicating Brg1 complexes with both gene activation and repression. Generally, the outcome of its action on a target gene is dictated by Brg1’s protein partners within SWI/SNF multiprotein complexes and by the gene-specific context. The many protein interactors of Brg1 and the versatile effector functions of SWI/SNF complexes pose a remarkably difficult challenge to investigate Brg1 acitivity on the mechanistic level in the embryo. Notably, Brg1-SWI/SNF complexes are found associated with both active and repressed chromatin elements [50] through interactions with DNA, histone modifications, and a large number of specific transcription factors (for detailed information on Brg1-SWI/SNF see reviews in [2, 4, 17]. In our study, we have not investigated genes that become upregulated/derepressed in BMO1 morphants. However, recent studies indicate that in human and mouse ESCs, BRG1-SWI/SNF complexes repress enhancer elements of lineage specification factors by modulating H3K27 acetylation or methylation levels [51, 52]. Based on this evidence, some of the genes, which are repressed by Brg1 in *Xenopus* before gastrulation, may also be important for embryonic development.

Highly informative for us were the GO-terms enriched in the downregulated gene cohort. They guided our analysis to establish Brg1 protein–and by inference corresponding SWI/SNF complexes–as regulator of early embryonic patterning. In evaluating the role of Brg1, one needs to distinguish carefully early and late phenotypes. The aberrant patterns of gene expression in BCNE, Nieuwkoop center and Spemann’s organizer are immediate consequences of the lack of Brg1 protein by temporal linkage with ZGA. Some phenotypes assessed late, like the axial defects characterizing the DMZ-injected BMO1 morphants, can still be attributed to direct effects, since the state of the underlying regulatory gene network is fixed at gastrulation. This has been demonstrated by classical experiments, in which the normal body plan of a tadpole is severely and irreversibly altered through treatments between the late one-cell to 32-cell stage, which modulate maternal Wnt-signaling activity [18, 53] The situation is different for late phenotypes that arise from defective cell differentiation over time, such as the malformed brains in embryos transplanted with a morphant BCNE (Fig 6) or injected dorso-animally with BMO1 (Fig 7). Since Brg1 is continuously expressed in the neuroectoderm and neural crest, these phenotypes may indicate a later requirement for Brg1 in brain and retina development [50].

What are the main developmental functions of Brg1 and how are they implemented in the process of embryogenesis? While previous studies implicated Brg1 in neuroectoderm differentiation [15–17, 54], our results demonstrate that this ATPase is required for neural plate formation. Programming the neural ground state involves a conserved gene regulatory network [24, 54]. Essential members include FoxD4I1, Zic1, Zic3 and Iroquois2, which are all significantly downregulated in Brg1 depleted embryos at late blastula (Figs 1 and S2, S2 Table). The installation of this network requires inhibition of Bmp signals in the prospective neural plate through secreted antagonists, three of which (*chordin*, *noggin*, *nr3*,) are expressed first in the BCNE region and subsequently in the organizer under the control of maternal Wnt/β-Catenin signaling [31, 55]. For these genes, our data define Brg1 as a coactivator of Wnt signaling, which helps install DGEP as first step to the formation of dorso-anterior tissues. Whether Brg1 could be even sufficient to induce DGEP alone, as suggested by secondary axis formation and ectopic chordin induction after ventral overexpression of *Xenopus* and human Brg1 proteins, is not clear at the moment. The ventral side of the embryo contains residual amounts of maternal Wnt11 protein, with which Brg1 might cooperate [20, 56].

In dorso-vegetal cells, the combination of maternal Wnt signaling plus high Nodal signaling establishes the Nieuwkoop Center [57]. This mode of regulation separates it from the BCNE, even though it overlaps with the BCNE region by cell lineage [39, 40]. The multifunctional BMP inhibitor *cerberus*, expressed in the Nieuwkoop Center is also BRG1-sensitive, as shown by dorso-vegetal BMO1 injections. Taken together, these results have identified three different embryonic territories to require Brg1 activity, and prevent a simple assignment of Brg1 function to either ventralizing or dorsalizing gene expression programs.

Interestingly not all Wnt targets depend on coactivation by Brg1-SWI/SNF, for instance the transcription factor gene *sia1* (Fig 4D). This helps to explain the peculiar observation that in BMO1 morphants *chordin* mRNA is selectively reduced in the BCNE, but is unaffected in the organizer (compare Figs 4 and S7). According to current models, maternal Wnt signaling induces transiently *sia1* expression (Brg1-independent; Fig 4D), which in turn activates *chordin* transcription (Brg1-sensitive; Figs 1, 4, S6 and 7) at the blastula stage (see Carnac et al., Development 1996); subsequently, *chordin* transcription is maintained through Nodal signaling (Brg1-independent; see Figs 5 and S7) in the gastrula organizer [27]. This model would predict that *sia1* maintains *chordin* in a Nodal-responsive state without transcriptional activation. A poised state is also a common theme for Wnt target genes in *Xenopus*, which are frequently bound by β-Catenin without eliciting a transcriptional response [58]. Transcriptional activation of Wnt targets has been proposed to occur through context-specific mechanisms, downstream of β-Catenin binding to chromatin. One such context could be recruitment of a Brg1-SWI/SNF chromatin remodeler. It should be highly informative to identify mechanisms, by which Brg1-SWI/SNF recognizes its targets at the blastula stage.

Gene expression domains in the gastrula organizer are generally less affected in BMO1 morphants than in the BCNE. Nevertheless, our data indicates a third function for Brg1 in activating transcription of the core BMP synexpression group (*bmp4*, *vent1 and vent2;* Fig 5, S2 Table), which specifies ventro-posterior tissues [59]. Interestingly, while their mRNA levels are globally reduced in radial BMO1 morphants at late blastula (S2 Table), the overlapping *vent1*/*vent2* expression domains are expanding into the organizer field. Here, they become coexpressed with DGEP genes like *gsc* and *otx2* (which specify anterior position) and the bmp antagonists *chordin* and *nr3* (Figs 5 and S7). This highly unusual pattern of genes in the organizer had also been generated by simultaneous knockdown of three BMP antagonists (*chordin*, *noggin*, *follistatin*) [36]. It indicates a significant weakening of the organizer function, which is reflected in our data by reduced expression of *nr3* and *otx2* within the organizer, and of the myogenic bHLH transcription factors *myoD* and *myf5* in non-organizer mesoderm (Figs 5 and S7). That the reduced bmp4/vent1/vent2 expression indeed contributes to the aberrant body plan, is apparent in radial morphants injected with 20ng BMO1 (S4 Fig). Although their blastomeres received the same amount of BMO1 oligo as embryos, which were injected only in DMZ or VMZ (i.e. 5ng/blastomere), the radial morphants were arrested at gastrulation and thus morphologically much stronger affected than DMZ or VMZ morphants (compare S4B Fig with Fig 2). It is plausible to assume that these perturbations in the organizer and non-organizer activities, together with reduced cerberus transcription in the Nieuwkoop Center are responsible for the massive loss of dorso-anterior structures seen in DMZ-injected BMO1 morphants (Figs 2 and 3) and for the posteriorized character of Brg1 depleted embryos, arrested permanently at the gastrula stage (S4 Fig). The invasion of vent1/vent2 gene expression into the organizer field suggests a basal failure in the cell determination process, which normally prevents activation of non-compatible gene expression programs in cells [60]. Indeed, non-compatible gene expression could mount a substantial problem given that almost 900 genes are misregulated in Brg1 morphants.

In summary, we have identified at least two independent functions for Brg1-SWI/SNF, which are essential for *Xenopus* embryonic patterning, namely being i) selective coactivator of maternal Wnt signaling on the prospective dorsal side of the embryo and ii) coactivator of the core *bmp* synexpression group on the prospective ventral side. However, more than 800 genes are misregulated in Brg1 depleted embryos at the blastula/gastrula transition. Is there a common denominator to explain Brg1’s impact on development? By quantitative filtering of global mRNA fluctuations at MBT, we have shown that Brg1 is predominantly required for genes with the highest burst of transcriptional activity. Mechanistically, Brg1-SWI/SNF could be involved to catalyze transitions from transcriptional silent to active chromatin states at promoters or facilitate long range interaction between distal enhancers and promoters [61], which in general become engaged at the blastula/gastrula transition [62]. Since many of the bursting genes are key developmental regulators, this may put BRG1 in a key position to raise their expression above threshold levels in preparation for the embryonic patterning process. Notably, both mathematical models and experimental evidence have detailed an enormous self-regulatory capacity within embryonic fields [63–65]. Why BRG1-depleted embryos cannot compensate a quantitatively insufficient ZGA and fail to restore axis formation through self-regulation, constitutes a key question for the future.

## Conclusion

We have shown that BRG1 protein is essential for early *Xenopus* development. BRG1 is involved in *de novo* activation of transcription at the Midblastula transition and is needed to achieve the transcriptional amplitude of genes with the highest-fold activation. Among these bursting genes are many regulators of embryonic axis formation. Targeted depletion of BRG1 protein levels results in the specific downregulation of key genes of the BCNE Center (*chordin*, *noggin*), the Nieuwkoop Center (*hhex*, *cer*) and the bmp-controlled ventral signaling territory (*vent1*, *vent2*). We propose that Brg1 fulfills a systemic function for late blastula stage transcription in preparation of embryonic pattern formation.

## Methods

### Ethics statement

Animal work has been conducted in accordance with Deutsches Tierschutzgesetz; experimental use of *Xenopus* embryos has been licensed by the Government of Oberbayern (AZ: 55.2.1.54–2532.6-7-12).

### Expression constructs and *in vitro* transcription

The ORF of *X*. *laevis* brg1 cDNA was generated by PCR from overlapping ESTs (Genbank acc. Nrs. AW766934, BG234591, BQ7288178) and subcloned into pCS2+. The full-length cDNA was verified by sequencing and deposited into GenBank (AY762376). For testing morpholino targeting efficiencies, the cDNA region from -77 to +617 of *X*. *laevis* brg1 (“BISH”) was fused in frame to the luciferase ORF in a gateway-compatible pCS2+ vector. All primer sequences are provided in the supplemental data section, S5 Table, part a. Open reading frames of human *brg1*, *brm* and *X*. *laevis iswi* (kind gift from Anthony Imbalzano and Paul Wade) were sub-cloned into pCS2+ for *in vitro* transcription. Capped mRNA for microinjection was synthesized as described [66].

### Morpholino oligonucleotides

Three antisense morpholino oligonucleotides against the translational start site of Brg1 mRNAs were purchased from GeneTools: All three are fully complimentary to transcripts from both X. laevis homeologs (NM_001086740.1 and BG554361); BMO1 and BMO2 also match perfectly the mRNA sequence of the S. *tropicalis* homolog (BG554361). BMO1: 5’- CCATTGGAGGGTCTGGGGTGGACAT-3’; BMO2: 5’-CAGGGAGAAGATCCAGTCACTGCTA-‘3; BMO3: 5’-GACATCACTGCAGGGAGAAGATCCA-‘3. The unrelated standard control Morpholino served as control for specificity.

### Luciferase-assay

Morpholino targeting efficiencies was determined *in vivo* with a *Brg1-luciferase* fusion mRNA. *Xenopus laevis* embryos were radially injected at the 2 cell stage with individual morpholino oligonucleotides (60ng/embryo). At the 8 cell stage, the four animal blastomeres were superinjected with synthetic *BISH-luciferase* mRNA (25pg/embryo). The embryos were cultivated until gastrulation (NF11) and luciferase activity was measured with Dual-Luciferase® Reporter Assay System (Promega). Samples consisted of cleared protein lysates from 5 pooled embryos per condition.

### Embryo handling

*X*. *laevis* and *X*. *tropicalis* eggs were collected, *in vitro* fertilized, microinjected and cultivated following standard procedures. Embryos were staged according to Nieuwkoop and Faber (1967). Radial injections were performed at the 2–4 cell stage, targeted injections were performed either at the 4 cell, 8 cell or 16 cell stage. For lineage tracing they were either injected with Alexa Fluor-488 Dextran, Alexa Fluor-594 Dextran (Invitrogen) or with 25-100 pg/blastomere of either nuclear *lacZ* or *eGFP* mRNA. Tissue transplantations were carried out in 0.8x MBS + Gentamycin in agarose-coated dishes. The transplant was kept in place with a cover slip for one hour, after which the transplanted embryo was transferred to a new dish in 0.1x MBS + gentamycin.

### RNA *in situ* hybridization and immunocytochemistry

Whole-mount RNA *in situ* hybridizations were performed as described [67]. Embryos were photographed with a Leica M205FA stereomicroscope. For immunocytochemistry anti-active Caspase3 antibody (1:20000, Promega) and anti-rabbit alkaline phosphatase-conjugated (1:1000, Chemicon) secondary antibody was used.

### Western blot analysis

For the production of xBrg1 specific monoclonal antibodies, N-terminal domain (amino acid 202–282) was cloned into pGEX4T3 expression vector (Amersham), expressed in *E*. *Coli* and purified to immunize rats. For quantitative measurement of Brg1 knockdown 15 embryos or eggs of each condition were collected and lysed in 75 μl NOP buffer [68] and centrifuged for 20 min at 14000 rpm to remove yolk plates. The supernatant was mixed with Roti®-Load 1 (Roth) and loaded onto an 8% SDS-PAGE. The separated proteins were blotted on nitrocellulose membrane and blocked for minimum 1 h at RT in 5% milk in PBSw. The membrane was incubated over night at 4°C with anti-Brg1 mab 3F1 (1:3) and as loading control anti α-tubulin (1:8000, Sigma). As secondary antibody the LiCor α-rat 800 and α-mouse 700 (1:10000, respectively) was used. The membrane was developed using the LiCOR system and the intensities were measured and quantified against the loading control.

### RNA extraction and qPCR sample preparation

Total RNA of 10 embryos was extracted using Trizol (Ambion) and phenol/chloroform. The RNA was precipitated with 70% Isopropanol and cleaned using the RNeasy Cleanup Kit (Qiagen) including DNAseI-on-column digestion. For qPCR analysis 1 μg of total RNA was transcribed with the DyNAmo CDNA Sythesis Kit (Bioenzym). For qPCR 5–20 ng cDNA was mixed with the Fast SYBR Green Master mix (Applied Biosystems) and amplified with a Lightcycler (Roche). Primer sequences are given in S5 Table, part b.

### Microarray analysis

For comparative MicroArray analysis *X*. *tropicalis* embryos were injected radially at the 2–4 cell stage with 30ng BMO1 or 60ng CoMO and cultivated until late Blastula stage. Per condition, 10 embryos were collected and RNA was extracted as described above. For the pre/postMBT Microarray, wildtype *X*. *tropicalis* embryos were collected. Ten embryos were pooled per sample. After fertilization we collected one sample in the 4cell stage as negative *GS17* control. For the preMBT time-point we collected embryos from around the ~1000 cell stage and then every 20–25 minutes for approximately 60-100min. We choose 20–25 min breaks depending on how long the embryos need in average for the first cell divisions. 120–150 minutes after collecting the last preMBT sample we started again to collect every 20–25 min samples until we observed dorsal lip formation. For all samples we extracted RNA the way it was described before. In order to find the preMBT sample closest to the MBT we performed qPCR analysis with MBT-marker *gs17*. For the preMBT time-point we took the last sample without *gs17* expression. As postMBT we choose the sample ~40min before dorsal lip formation, in accordance to the developmental age, at which the comparative Microarray analysis was performed.

The quality of the extracted RNA was controlled for both experimental setups with the Bioanalyzer and handed to the “Facility of Functional Genomics” at the Gene Center, Munich for microarray performance on an Affymetrix *Xenopus tropicalis* genome Array.

Microarray preprocessing was conducted separately for the two experimental sets (Brg1 knockdown and MBT) using R/Bioconductor (www.bioconductor.org). If not indicated otherwise, we used standard parameters in all functions calls. Expression values were calculated using ‘gcrma’. Probe sets were kept for differential expression analysis if there were more ‘present’ calls (calculated using ‘mas5calls’) in one of the treatment groups than non-‘present’ calls, if their expression level variance was higher than zero across all arrays and if the probe set had an Entrez identifier annotation according to the Entrez database with a date stamp of 2011-Mar16. One gene to many probe set relationships were resolved by retaining only the probe set with the highest interquartile range across all arrays. Differential expression statistics were obtained using a linear model (library ‘limma’). A significant response was defined if the adjusted p-value was smaller than 0.05.

### Statistical analysis

For all embryonic quantitative analysis (morphological phenotype, WMISH, qRT/PCR) SEM are displayed and the statistical analysis was performed using two-tailed, Paired Student’s *t*-test. For transcriptome analysis see microarray section.

## Supporting information

S1 Fig Three *brg1* specific antisense Morpholinos display different translation blocking efficiencies. In each condition, 60ng of either CoMO or BMO1, BMO2 or BMO3 were radially injected at the 2–4 cell stage into the animal pole of *X*. *laevis* embryos. At 8-cell stage, the Luciferase signals from BMO injected embryos were normalized to CoMO signal intensity (n = 3 independent biological experiments).
(TIF)

S2 Fig Apoptosis is not upregulated in BMO1 morphants. *X*. *laevis* embryos were radially injected with CoMo or BMO1 as in Fig 1 and immunostained for activated Caspase-3 protein at early gastrula stage (NF10.5). No apoptotic cells were detected. As positive control, the embryo in panel A was injected with an expression plasmid for the proapoptotic factor FADD (60pg/embryo) into the DMZ. Dashed white line delineates the field, where FADD induced apoptosis; white asterisks indicate background staining on the blastocoel walls; inserts: dorso-vegetal views. Black dashed line marks the extent of the blastopore lip.
(TIF)

S3 Fig Functional annotation and validation of BMO1 responding genes. Panels **(A, B)** show the top five GO-terms for up- or downregulated genes in *X*. *tropicalis* BMO1 morphants. Panels (**C, D**)—qRT/PCR based verification of microarray data in radially injected *X*. *tropicalis* embryos (n = 5–8 independent experiments/gene). *Noggin* was included based on its function as neural inducer, even though the microarray contained no probeset for this gene. Asterisks mark genes, which were significantly downregulated in the qRT/PCR analysis. Panel **(C)** Blastula signaling centers. *Chordin*, *noggin*, *follistatin* and *bmp4* mRNAs were downregulated. Panel **(D)** shows germ layers markers: *zic1*, *sox21* and *gata4* were downregulated, while the upregulation of *egr1* was not statistically verified. RNA *in situ* analysis of radially injected *X*. *tropicalis* morphant blastulae: *foxd4l1* **(E, F)**, *noggin* **(G, H)** and *zic2* **(I, K)**. **(E’-K’)** show corresponding sagittal sections. Whereas *foxd4l1* and *noggin* are downregulated in BMO1 morphants, *zic2* gene expression is not changed (n = two biological replicates).
(TIF)

S4 Fig Dose-dependent consequences of systemic BMO1 depletion in *X*. *laevis*. BMO1 morpholinos were injected four times into the animal pole region at the two- to four-cell stage (“radial” injection type). Panel **(A)**: Typical morphology of embryos injected with increasing dose of BMO1. UI–uninjected siblings. BRG1 depleted embryos get arrested in gastrulation. Top rows: brightfield image; bottom row: green fluorescence from coinjected Alexa488 dextran. Panel **(B)** shows representative images of embryos that have survived until heartbeat stage (NF34). While most embryos are still arrested in gastrulation, some embryos injected with the lowest BMO1 dose (20 ng) have completed gastrulation but are arrested at open neural plate stage. Axial structures and closed neural plates were never observed. Panel (**C)** gives quantification of the BMO1 titration (n = 3–5 biological replicates/condition).
(TIF)

S5 Fig Ventral overexpression of Brg1 induces incomplete secondary body axes. *X*. *laevis* embryos were injected in one ventral blastomere at the four cell stage with the following reagents: **(A)** 100pg *nlacZ* mRNA. The control embryo develops a normal shape. **(B)** 1ng b*rg1* mRNA results in a truncated secondary axis. (**C**) Frequency of 2° axes induction. * p-value ≤ 0.05. Panels (**D-I)** WMISH for the muscle actin gene *actc1*. **(D-F’)** Ventrally injected embryo with 100pg *nlacZ* mRNA in lateral **(D, E**) and in dorsal view (**F**). **(F’)** is a close-up of the area marked in F, showing nlacZ stained nuclei in myocytes of the second axis. Panels **(G-I’**) Ventrally injected Brg1 overexpressing embryo from lateral view **(G, H**) and dorsal view **(I**). **(I’)** shows a close-up of the area marked in **(I)**; the arrow points to the bifurcation of primary and secondary axes, marked by nlacZ staining.
(TIF)

S6 Fig Human *brg1* mRNA induces ectopic *chordin* expression in prospective ventral ectoderm. **(A)** *X*. *laevis* embryos were injected at the 4 cell stage in one ventral blastomere with either 500pg or 1ng human *brg1* mRNA. At late Blastula stage (NF9) the embryos were fixed and stained for *chordin* mRNA. At this stage, *chordin* is normally expressed in the dorsal BCNE signaling center in prospective neuroectoderm. The ventral overexpression of human *brg1* mRNA induces a second chordin expression zone on the ventral side in prospective epidermis. *nlacZ* mRNA was coinjected as lineage tracer. Panel **(B)** gives quantification (n = 2 biological replicates/condition).
(TIF)

S7 Fig Mesodermal marker genes in *X*. *laevis* BMO1 morphant gastrulae. Radially injected *X*. *laevis* embryos with CoMO or BMO1 (40ng/embryo) were stained for mRNAs indicated on the left (vegetal views, dorsal on top). Each marker was analyzed in 2–4 independent experiments, and classified into normal or reduced expression. Numbers in panels tell the number of embryos with the shown expression pattern; the graphs on the right translate this information in % penetrance.
(TIF)

S8 Fig Orthotopic BCNE center transplantation reveals autonomous requirement for Brg1 in head formation. **(A)** The experimental scheme of BCNE center transplantation (*X*. *laevis*). Note that the BCNE region is labeled with different colors in both the donor and host embryos to facilitate orthotopic grafting. **(B-D)** images represent dorsal views of BCNE transplanted tadpoles as merged brightfield/Alexagreen-fluorescent views. **C** and **D** show embrryos with transplanted Brg1 morphant BCNE. After recording, these embryos were singly subjected to WMISH against *otx2* mRNA. **(B’-D’)** Rows detail *otx2* mRNA staining seen from left, right side and dorsal view. In wt embryos, *otx2* is expressed in forebrain (including retina and olgactory epithelium [fb]), midbrain (mb) and hindbrain (hb) areas. Note the symmetric expression in the wildtype transplant, and the amorphous structure of the *otx2*-positiv BMO1 morphant tissue. **(E)** Quanitification of *otx2* mRNA pattern in WT (n = 17) and BMO1 morphant (n = 24) transplants. Differences for the retina stain were significant with *, p ≤ 0,007.
(TIF)

S9 Fig Dorso-animal and dorso-vegetal control injections. **(A-D)** *X*. *laevis* embryos injected at the 8 cell stage dorso-vegetally with either CoMO or BMO1 analysed for mRNA staining of BCNE genes *chordin* **(A, B)** and *noggin* **(C, D)**. (**E**) Quantification of the two markers. (**F-I**) display the mRNA pattern of *cerberus* **(F, G)** and *hhex* **(H, I)** in dorso-animally injected embryos with either CoMO or BMO1. Note the partial overlap of *chordin* and *noggin* expression domain with the DV-injected area. (**K**) Quantification of *chordin* and *noggin* mRNA expression. *, p-value ≤ 0.05.
(TIF)

S10 Fig Brg1 is required for the transcriptional burst at the MBT. Panel **(A)** displays experimental scheme of sample collection for genome-wide comparison of preMBT versus postMBT transcriptomes (*X*. *tropicalis*). **(B)** The preMBT sample closest to the MBT (see sample #3 in red) was identified by qRT/PCR analysis for the marker gene *gs17*. *Gs17* mRNA levels of the “early” samples 1–5 were normalized to the value of four cell stage embryos (n = 3 biological replicates). The postMBT sample (here #2 in red) was chosen as the one being harvested 40 min before the appearance of the blastoporus pigmentation lip in the sibling cohorts. This time point correlates with the late blastula stage used for the BMO1 microarray analysis of Fig 1. **(C**) The top 4 enriched GO-terms in the class of highly upregulated genes at MBT. **(D)** Heatmap providing mean expression levels at the pre- and postMBT timepoints for genes of the GO-term “nervous system development”, ranked by amplitude of mRNA increase (log2 fold-change). Genes that were downregulated in the BMO1-morphant transcriptome, compared to the control morphant state are marked with an asterisk. Like in the term “pattern specification process” only genes that show a marked upregulation of mRNA after MBT were affected by the Brg1 protein knockdown.
(TIF)

S1 Table Genes ≥ 0,59 log_2_ fold (1,5 fold) upregulated upon Brg1 knockdown. (DOCX)

S2 Table Genes ≥ 0,59 log_2_ fold (1,5 fold) downregulated upon Brg1 knockdown. (DOCX)

S3 Table Morphological rescue of BMO1 morphants by *hbrg1*, *hbrm* and *xISWI*. (DOCX)

S4 Table Genes ≥ 2,5 log_2_ fold (5,6 fold) upregulated after MBT. (DOCX)

S5 Table Oligonucleotide sequences. (DOCX)
