# Supplementary figures and images for: Brg1 chromatin remodeling ATPase balances germ layer patterning by amplifying the transcriptional burst at midblastula transition
Source: PLoS Genet. 2017 May 12;13(5):e1006757. doi: 10.1371/journal.pgen.1006757 (PMC5428918; doi:10.1371/journal.pgen.1006757)

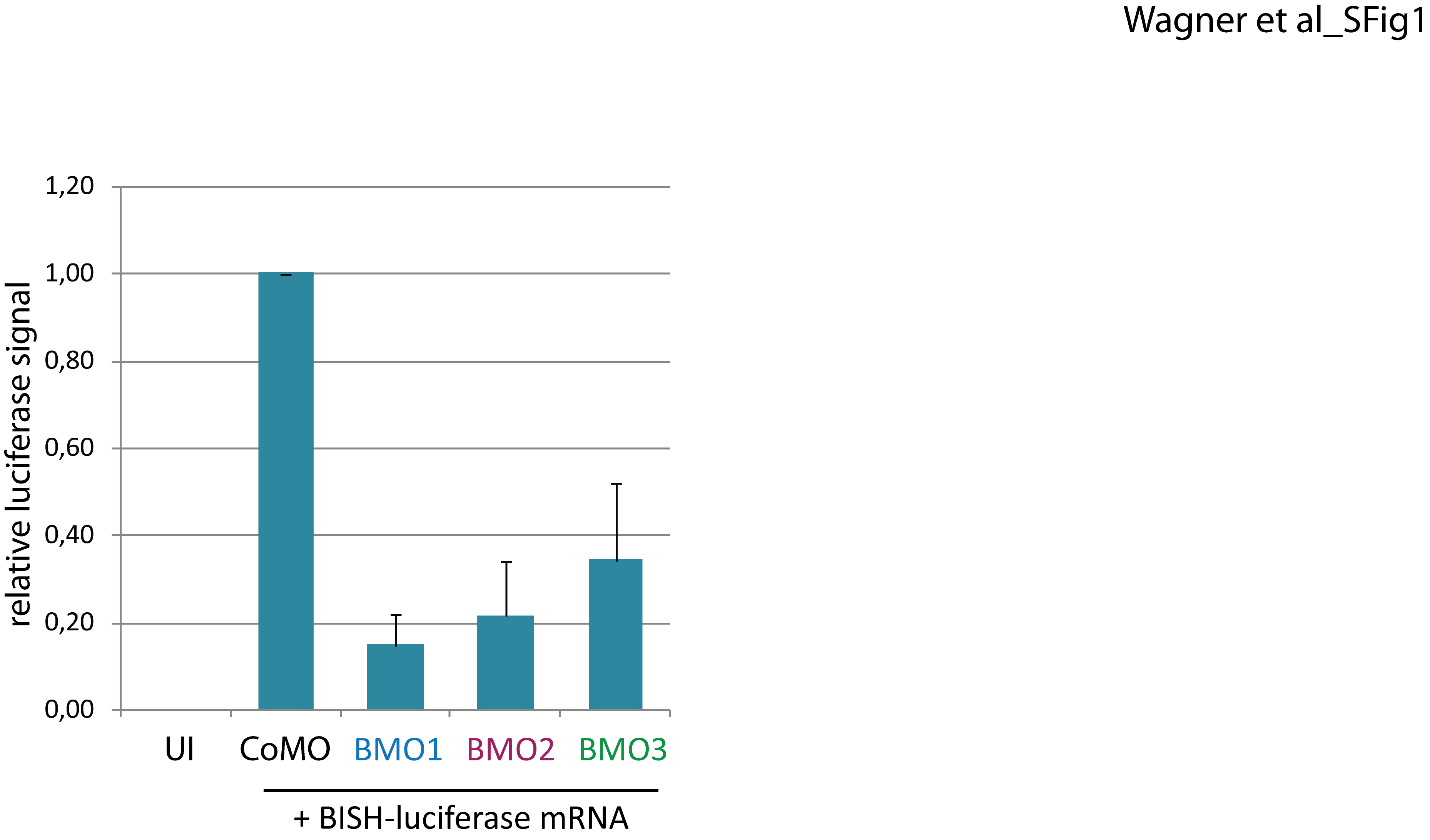

Supplement: S1 Fig — In each condition, 60ng of either CoMO or BMO1, BMO2 or BMO3 were radially injected at the 2–4 cell stage into the animal pole of X. laevis embryos. At 8-cell stage, the Luciferase signals from BMO injected embryos were normalized to CoMO signal intensity (n = 3 independent biological experiments). (TIF) [file pgen.1006757.s001.tif]

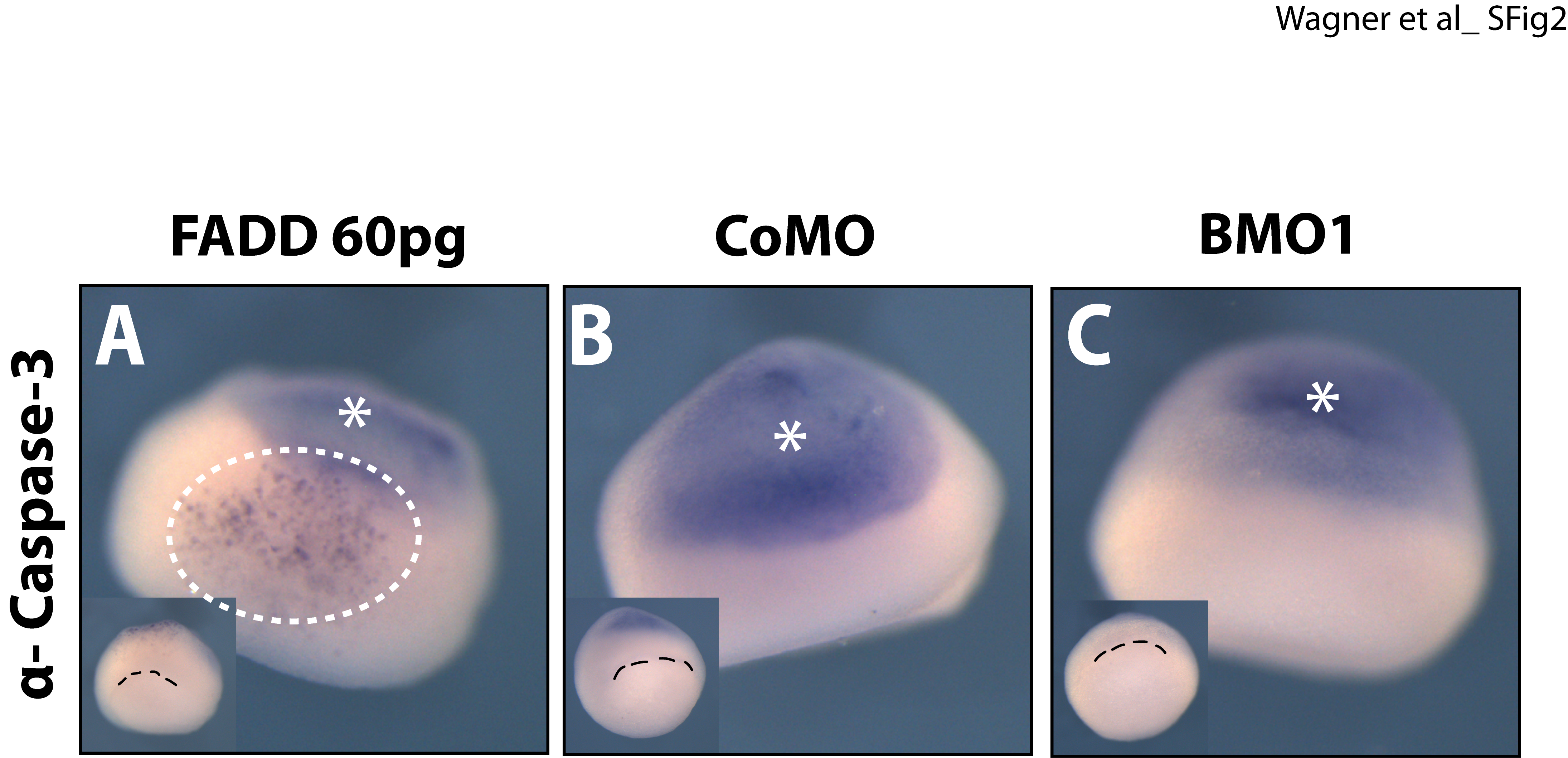

Supplement: S2 Fig — X. laevis embryos were radially injected with CoMo or BMO1 as in Fig 1 and immunostained for activated Caspase-3 protein at early gastrula stage (NF10.5). No apoptotic cells were detected. As positive control, the embryo in panel A was injected with an expression plasmid for the proapoptotic factor FADD (60pg/embryo) into the DMZ. Dashed white line delineates the field, where FADD induced apoptosis; white asterisks indicate background staining on the blastocoel walls; inserts: dorso-vegetal views. Black dashed line marks the extent of the blastopore lip. (TIF) [file pgen.1006757.s002.tif]

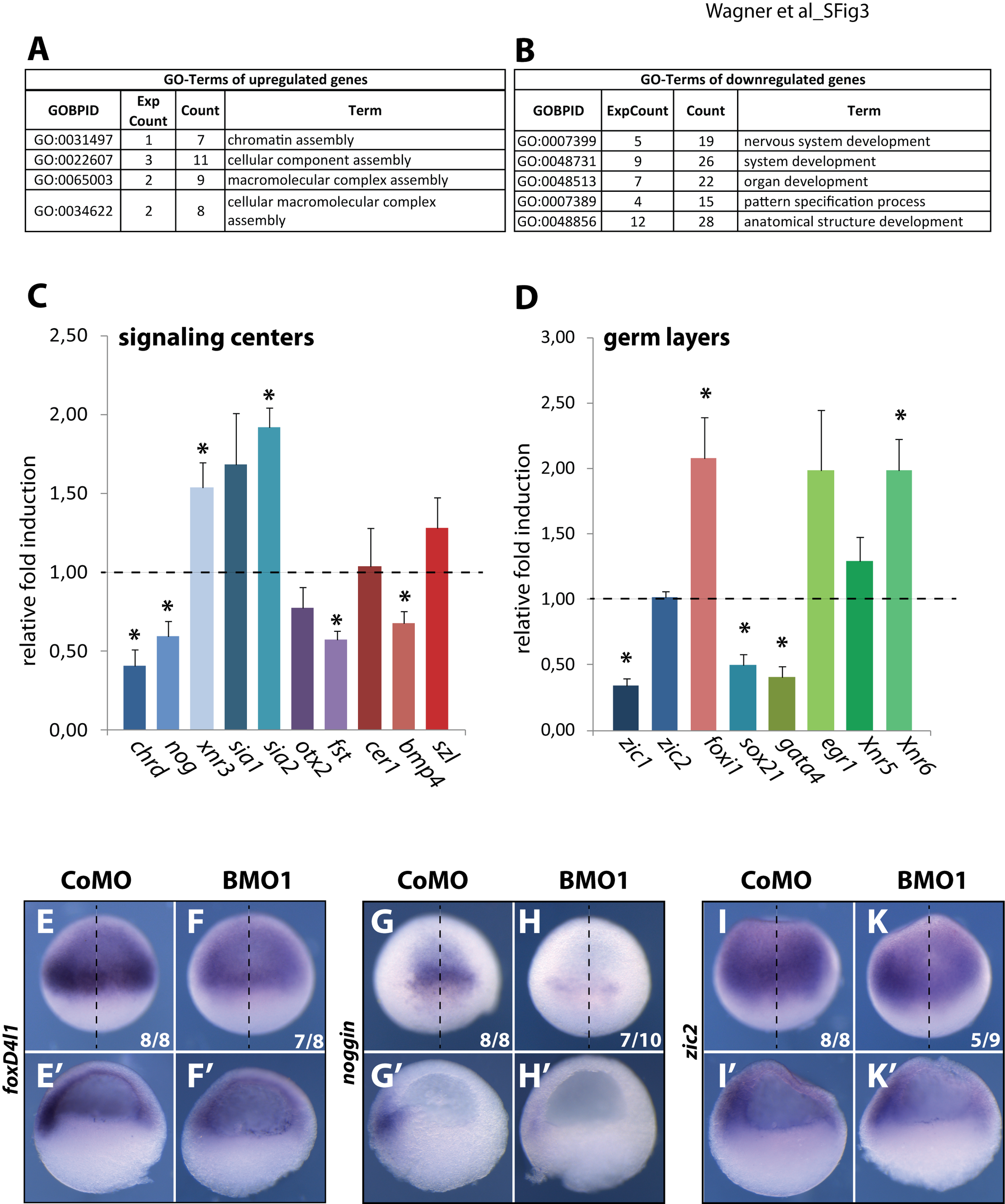

Supplement: S3 Fig — Panels (A, B) show the top five GO-terms for up- or downregulated genes in X. tropicalis BMO1 morphants. Panels (C, D)—qRT/PCR based verification of microarray data in radially injected X. tropicalis embryos (n = 5–8 independent experiments/gene). Noggin was included based on its function as neural inducer, even though the microarray contained no probeset for this gene. Asterisks mark genes, which were significantly downregulated in the qRT/PCR analysis. Panel (C) Blastula signaling centers. Chordin, noggin, follistatin and bmp4 mRNAs were downregulated. Panel (D) shows germ layers markers: zic1, sox21 and gata4 were downregulated, while the upregulation of egr1 was not statistically verified. RNA in situ analysis of radially injected X. tropicalis morphant blastulae: foxd4l1 (E, F), noggin (G, H) and zic2 (I, K). (E’-K’) show corresponding sagittal sections. Whereas foxd4l1 and noggin are downregulated in BMO1 morphants, zic2 gene expression is not changed (n = two biological replicates). (TIF) [file pgen.1006757.s003.tif]

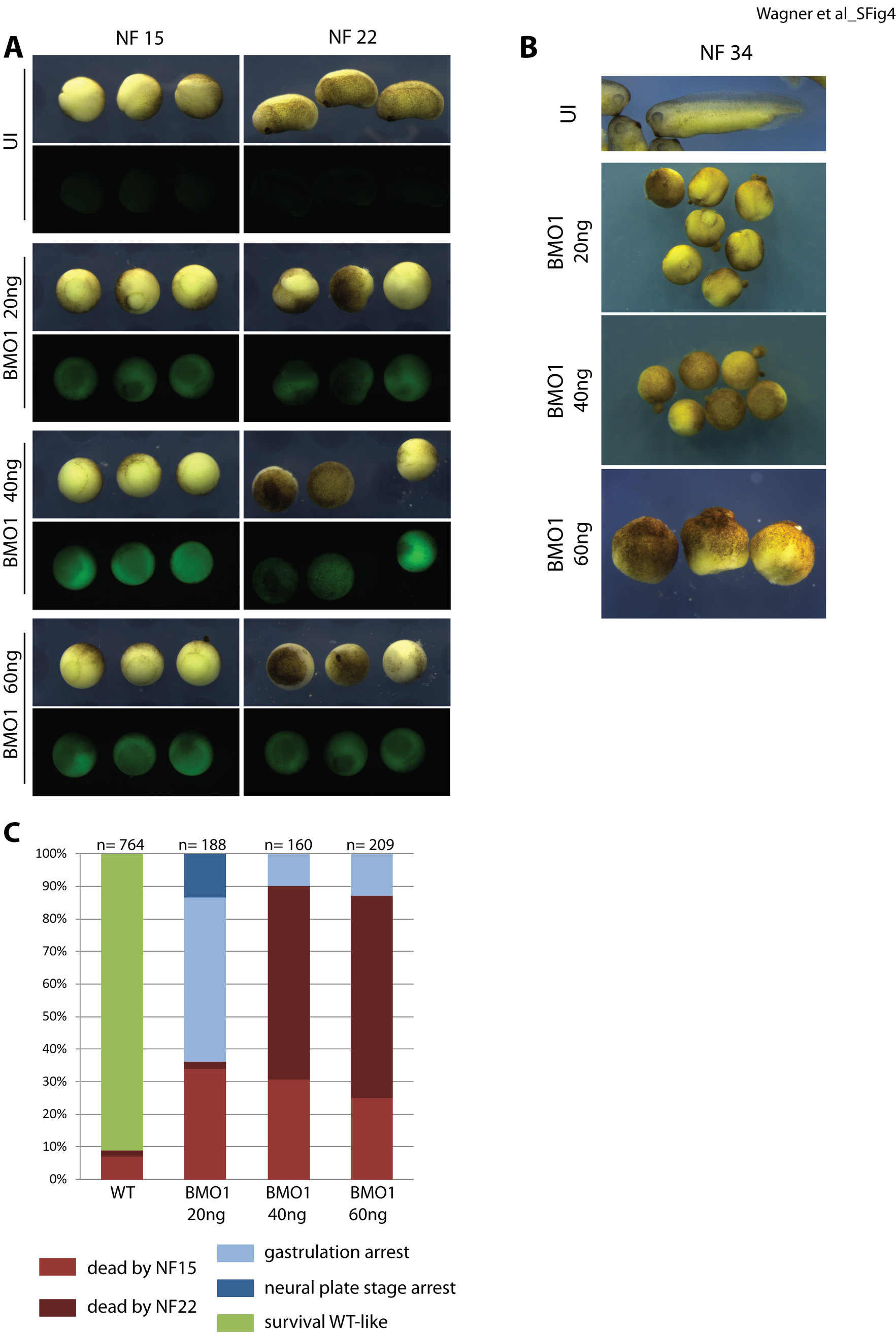

Supplement: S4 Fig — BMO1 morpholinos were injected four times into the animal pole region at the two- to four-cell stage (“radial” injection type). Panel (A): Typical morphology of embryos injected with increasing dose of BMO1. UI–uninjected siblings. BRG1 depleted embryos get arrested in gastrulation. Top rows: brightfield image; bottom row: green fluorescence from coinjected Alexa488 dextran. Panel (B) shows representative images of embryos that have survived until heartbeat stage (NF34). While most embryos are still arrested in gastrulation, some embryos injected with the lowest BMO1 dose (20 ng) have completed gastrulation but are arrested at open neural plate stage. Axial structures and closed neural plates were never observed. Panel (C) gives quantification of the BMO1 titration (n = 3–5 biological replicates/condition). (TIF) [file pgen.1006757.s004.tif]

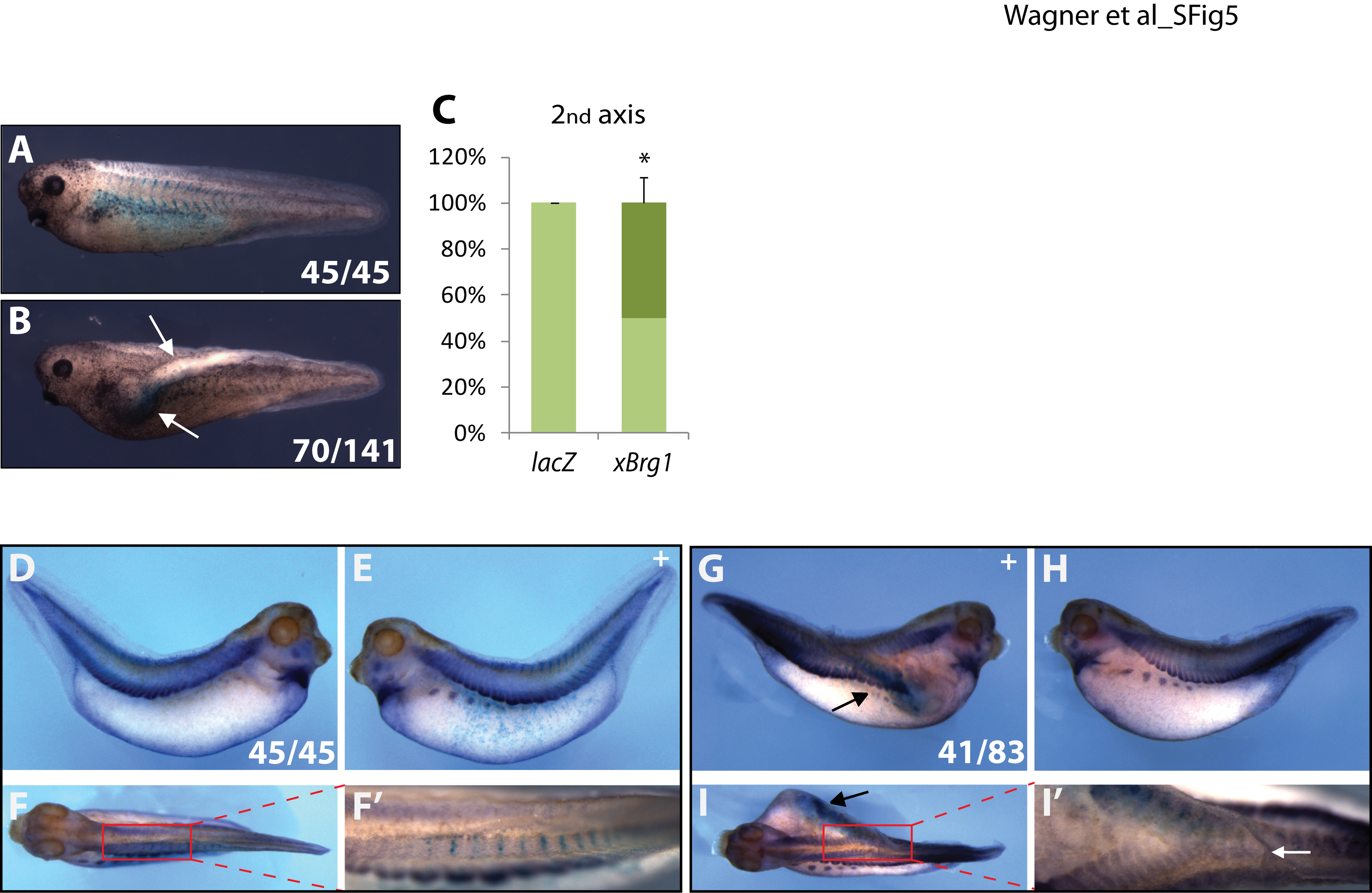

Supplement: S5 Fig — X. laevis embryos were injected in one ventral blastomere at the four cell stage with the following reagents: (A) 100pg nlacZ mRNA. The control embryo develops a normal shape. (B) 1ng brg1 mRNA results in a truncated secondary axis. (C) Frequency of 2° axes induction. * p-value ≤ 0.05. Panels (D-I) WMISH for the muscle actin gene actc1. (D-F’) Ventrally injected embryo with 100pg nlacZ mRNA in lateral (D, E) and in dorsal view (F). (F’) is a close-up of the area marked in F, showing nlacZ stained nuclei in myocytes of the second axis. Panels (G-I’) Ventrally injected Brg1 overexpressing embryo from lateral view (G, H) and dorsal view (I). (I’) shows a close-up of the area marked in (I); the arrow points to the bifurcation of primary and secondary axes, marked by nlacZ staining. (TIF) [file pgen.1006757.s005.tif]

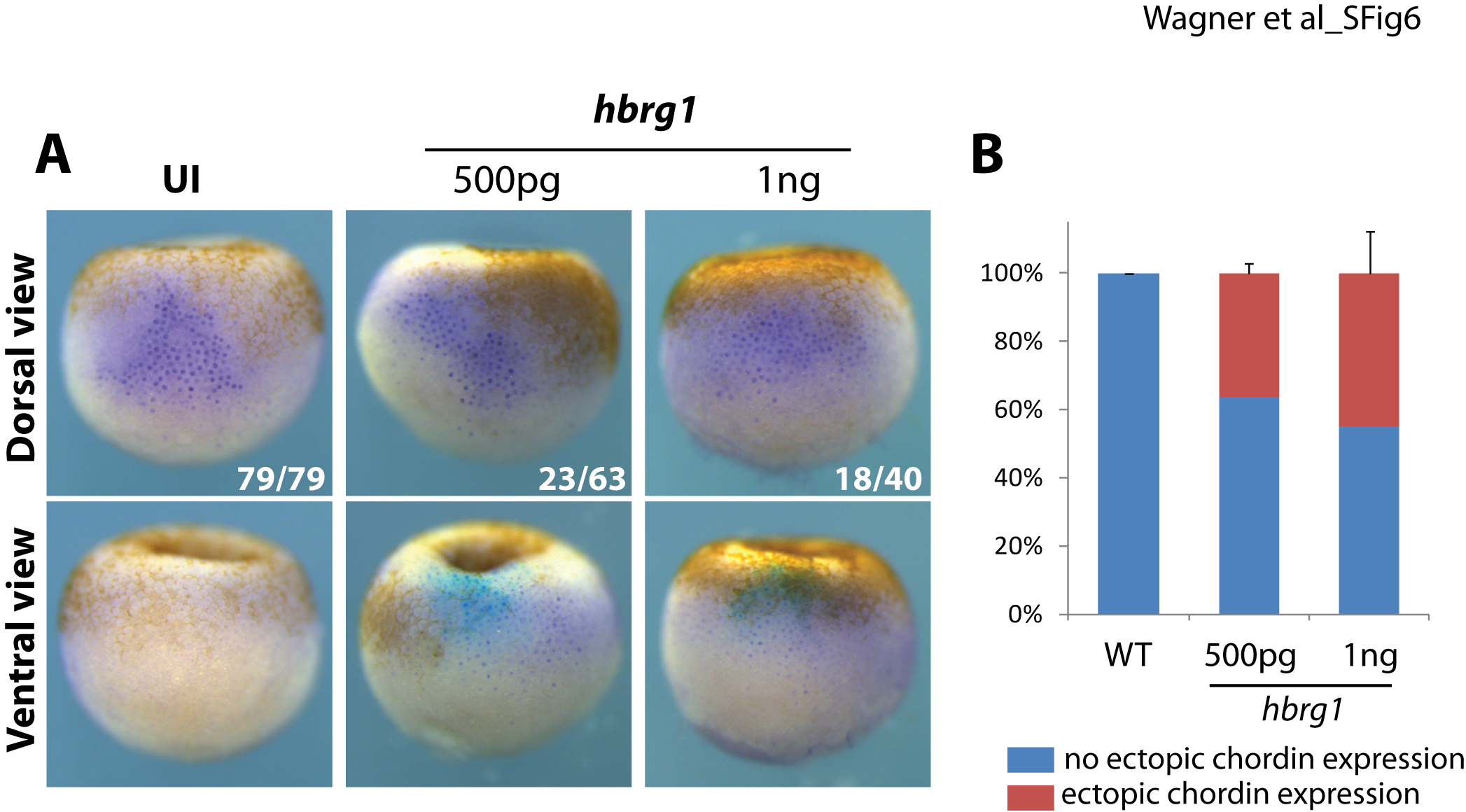

Supplement: S6 Fig — (A) X. laevis embryos were injected at the 4 cell stage in one ventral blastomere with either 500pg or 1ng human brg1 mRNA. At late Blastula stage (NF9) the embryos were fixed and stained for chordin mRNA. At this stage, chordin is normally expressed in the dorsal BCNE signaling center in prospective neuroectoderm. The ventral overexpression of human brg1 mRNA induces a second chordin expression zone on the ventral side in prospective epidermis. nlacZ mRNA was coinjected as lineage tracer. Panel (B) gives quantification (n = 2 biological replicates/condition). (TIF) [file pgen.1006757.s006.tif]

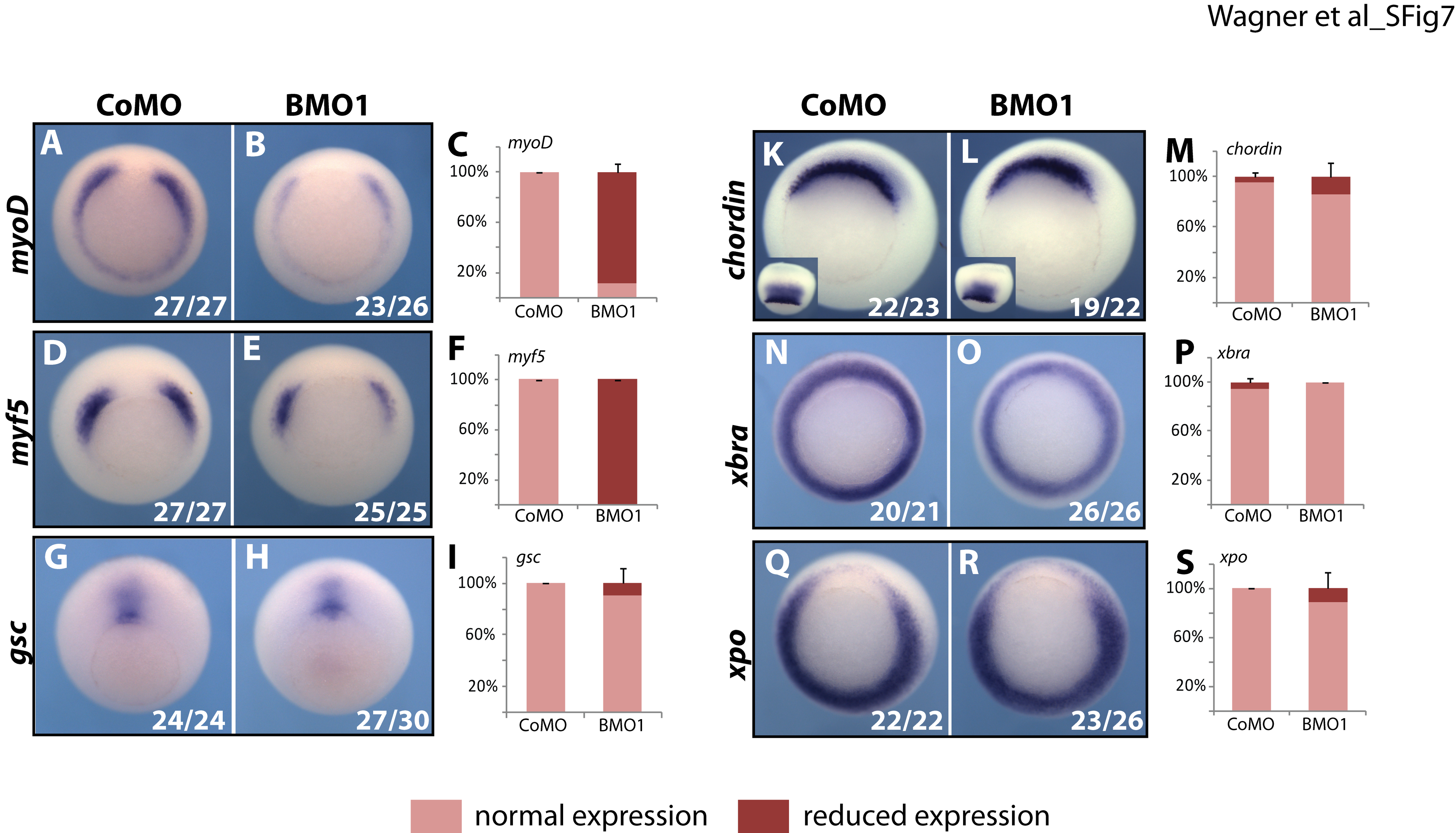

Supplement: S7 Fig — Radially injected X. laevis embryos with CoMO or BMO1 (40ng/embryo) were stained for mRNAs indicated on the left (vegetal views, dorsal on top). Each marker was analyzed in 2–4 independent experiments, and classified into normal or reduced expression. Numbers in panels tell the number of embryos with the shown expression pattern; the graphs on the right translate this information in % penetrance. (TIF) [file pgen.1006757.s007.tif]

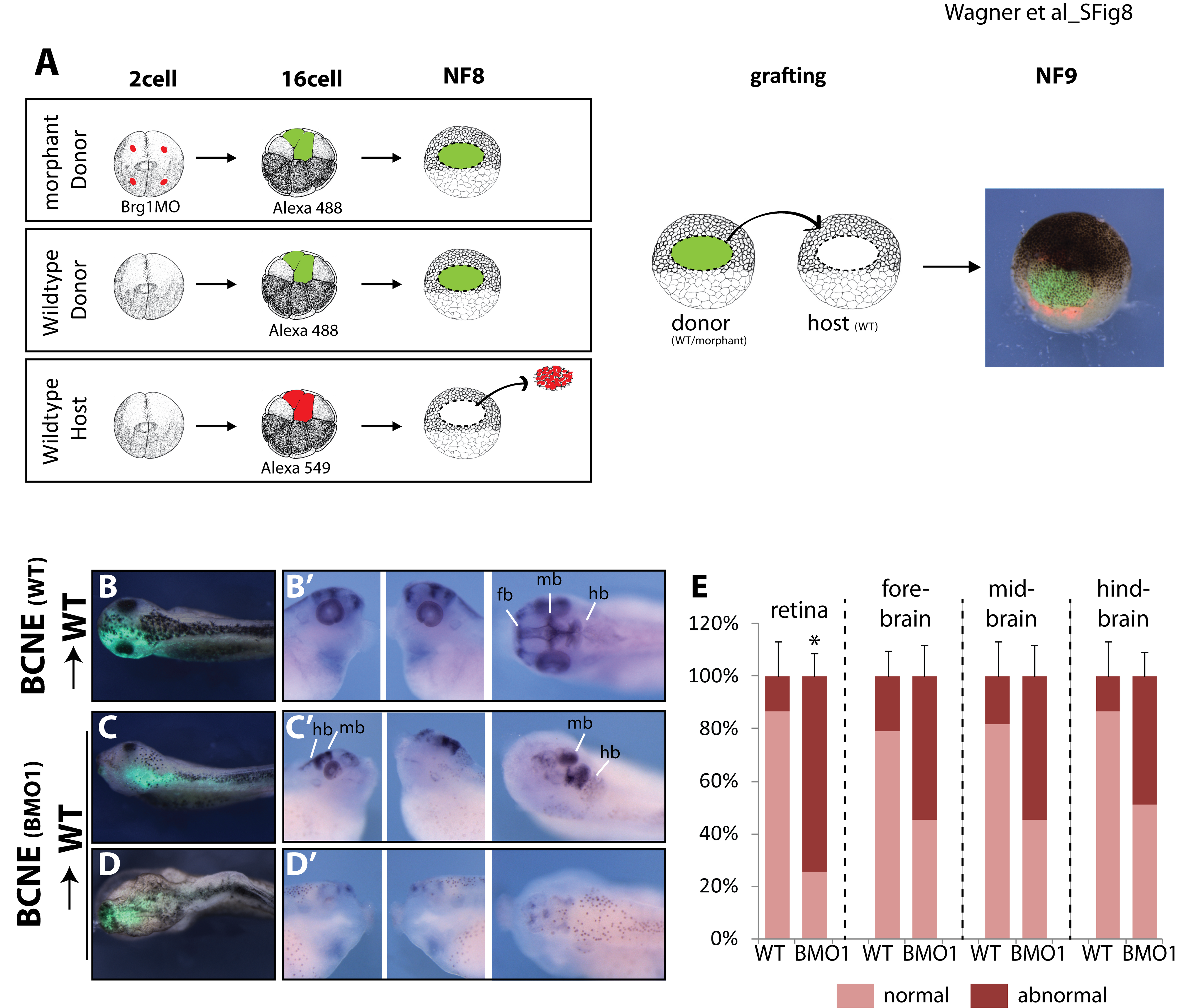

Supplement: S8 Fig — (A) The experimental scheme of BCNE center transplantation (X. laevis). Note that the BCNE region is labeled with different colors in both the donor and host embryos to facilitate orthotopic grafting. (B-D) images represent dorsal views of BCNE transplanted tadpoles as merged brightfield/Alexagreen-fluorescent views. C and D show embrryos with transplanted Brg1 morphant BCNE. After recording, these embryos were singly subjected to WMISH against otx2 mRNA. (B’-D’) Rows detail otx2 mRNA staining seen from left, right side and dorsal view. In wt embryos, otx2 is expressed in forebrain (including retina and olgactory epithelium [fb]), midbrain (mb) and hindbrain (hb) areas. Note the symmetric expression in the wildtype transplant, and the amorphous structure of the otx2-positiv BMO1 morphant tissue. (E) Quanitification of otx2 mRNA pattern in WT (n = 17) and BMO1 morphant (n = 24) transplants. Differences for the retina stain were significant with *, p ≤ 0,007. (TIF) [file pgen.1006757.s008.tif]

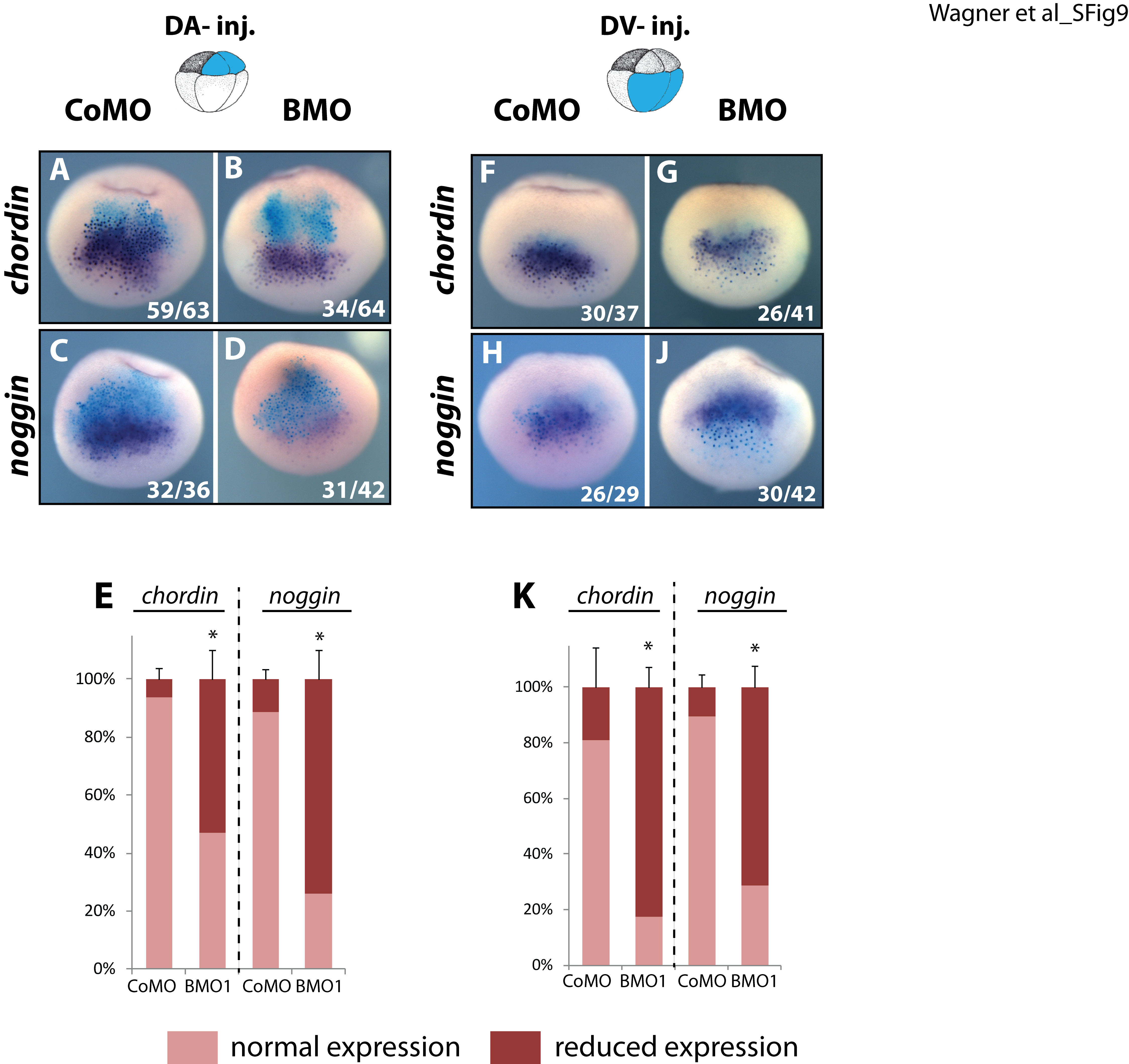

Supplement: S9 Fig — (A-D) X. laevis embryos injected at the 8 cell stage dorso-vegetally with either CoMO or BMO1 analysed for mRNA staining of BCNE genes chordin (A, B) and noggin (C, D). (E) Quantification of the two markers. (F-I) display the mRNA pattern of cerberus (F, G) and hhex (H, I) in dorso-animally injected embryos with either CoMO or BMO1. Note the partial overlap of chordin and noggin expression domain with the DV-injected area. (K) Quantification of chordin and noggin mRNA expression. *, p-value ≤ 0.05. (TIF) [file pgen.1006757.s009.tif]

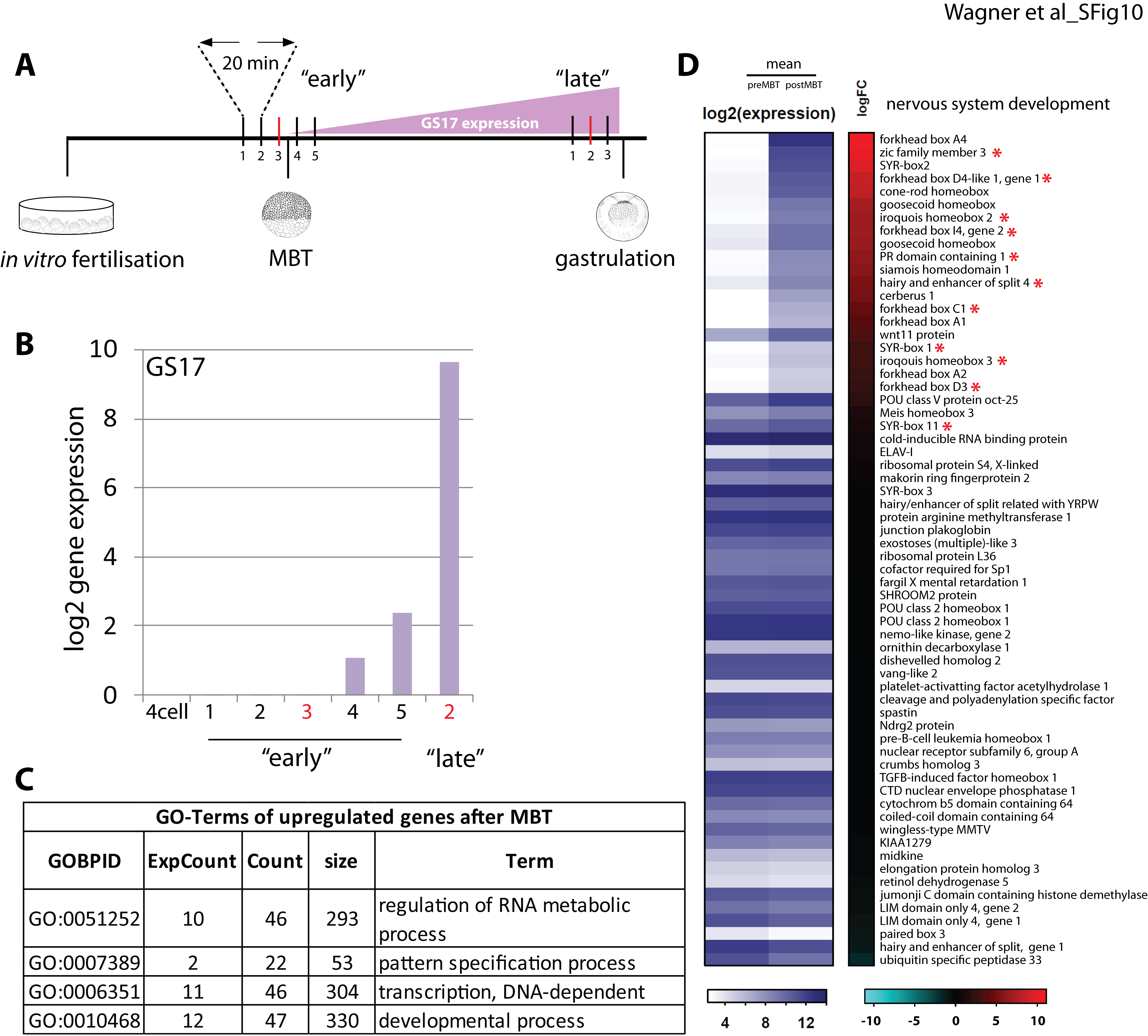

Supplement: S10 Fig — Panel (A) displays experimental scheme of sample collection for genome-wide comparison of preMBT versus postMBT transcriptomes (X. tropicalis). (B) The preMBT sample closest to the MBT (see sample #3 in red) was identified by qRT/PCR analysis for the marker gene gs17. Gs17 mRNA levels of the “early” samples 1–5 were normalized to the value of four cell stage embryos (n = 3 biological replicates). The postMBT sample (here #2 in red) was chosen as the one being harvested 40 min before the appearance of the blastoporus pigmentation lip in the sibling cohorts. This time point correlates with the late blastula stage used for the BMO1 microarray analysis of Fig 1. (C) The top 4 enriched GO-terms in the class of highly upregulated genes at MBT. (D) Heatmap providing mean expression levels at the pre- and postMBT timepoints for genes of the GO-term “nervous system development”, ranked by amplitude of mRNA increase (log2 fold-change). Genes that were downregulated in the BMO1-morphant transcriptome, compared to the control morphant state are marked with an asterisk. Like in the term “pattern specification process” only genes that show a marked upregulation of mRNA after MBT were affected by the Brg1 protein knockdown. (TIF) [file pgen.1006757.s010.tif]
