# Supplementary material for: Brg1 chromatin remodeling ATPase balances germ layer patterning by amplifying the transcriptional burst at midblastula transition
Source: PLoS Genet. 2017 May 12;13(5):e1006757. doi: 10.1371/journal.pgen.1006757 (PMC5428918; doi:10.1371/journal.pgen.1006757)
Supplement: S1 Table — (DOCX) [file pgen.1006757.s011.docx]

Table S1**: genes ≥ 0,59 log_2_ fold (1,5 fold) upregulated upon Brg1 knockdown**

| **Set-probe number** | **fold change [log2]** | **gene name** | **symbol** |
| --- | --- | --- | --- |
| StrEns.5201.1.S1_s_at | 3,719 | WNT1-inducible-signaling pathway protein 3-like | LOC100488354 |
| Str.40271.2.S1_a_at | 3,227 | Hypothetical protein LOC550005 | LOC550005 |
| Str.9570.1.S1_at | 3,080 | ribophorin II | rpn2 |
| Str.27732.1.S1_at | 2,895 | GTP binding protein 5 (putative) | gtpbp5 |
| Str.20978.1.S1_at | 2,344 | Hypothetical protein LOC100158502 | LOC100158502 |
| Str.51787.1.S1_at | 2,153 | histone cluster 1, H2ah | hist1h2ah |
| Str.51882.1.S1_at | 2,094 | chromosome 20 open reading frame 29 | c20orf29 |
| Str.40271.1.S1_at | 2,005 | Hypothetical protein LOC549623 | TGas006m08.1 |
| Str.16459.1.S1_at | 1,698 | hect (homologous to the E6-AP (UBE3A) carboxyl terminus) domain and RCC1 (CHC1)-like domain (RLD) 1 | herc1 |
| Str.3409.1.A1_at | 1,583 | GTP binding protein 2 | gtpbp2 |
| Str.24465.1.S1_at | 1,562 | mal, T-cell differentiation protein 2 | mal2 |
| Str.1273.1.S1_at | 1,499 | histone H4-like | LOC100496593 |
| StrJgi.934.1.S1_s_at | 1,452 | protein kinase (cAMP-dependent, catalytic) inhibitor gamma | pkig |
| Str.18904.1.S1_at | 1,433 | junctophilin 1 | jph1 |
| Str.27312.1.S1_at | 1,356 | histone cluster 2, H2ab | hist2h2ab |
| Str.2030.1.A1_at | 1,250 | Frizzled homolog 3 | fzd3 |
| StrEns.12239.1.S1_s_at | 1,230 | hypothetical protein hypothetical protein | LOC100491313 LOC100496030 |
| Str.5781.1.A1_at | 1,199 | Hypothetical protein MGC147532 | MGC147532 |
| Str.25548.1.A1_at | 1,150 | amyloid protein-binding protein 2-like | LOC100487715 |
| StrEns.8996.1.S1_a_at | 1,142 | hypothetical protein LOC100496030 | LOC100496030 |
| Str.41102.1.S1_at | 1,047 | CCAAT/enhancer binding protein (C/EBP), delta | cebpd |
| Str.37449.1.A1_at | 1,007 | poly (ADP-ribose) polymerase family, member 4 | parp4 |
| Str.8423.1.S1_at | 1,004 | chromosome 6 open reading frame 211 | c6orf211 |
| Str.3950.1.S2_at | 0,989 | integrin alpha-5-like | LOC100492002 |
| Str.8642.1.S1_at | 0,987 | trans-1,2-dihydrobenzene-1,2-diol dehydrogenase | dhdh |
| StrAffx.105.1.S1_s_at | 0,975 | synaptotagmin 7 | syt7 |
| Str.6540.2.A1_at | 0,967 | Vac14 homolog | vac14 |
| Str.51459.1.S1_at | 0,966 | neurobeachin | nbea |
| Str.16785.2.S1_at | 0,954 | oxidase (cytochrome c) assembly 1-like | oxa1l |
| Str.50909.1.S1_at | 0,953 | activating transcription factor 7 | atf7 |
| Str.14144.2.A1_at | 0,934 | zinc finger and BTB domain containing 5 | zbtb5 |
| Str.21484.1.A1_s_at | 0,907 | pyruvate dehyrogenase phosphatase catalytic subunit 1 | pdp1 |
| Str.11152.1.S2_at | 0,904 | cornichon homolog 4 | cnih4 |
| Str.10066.1.S1_at | 0,904 | mesendoderm nuclear factor, gene 1 | menf.1 |
| Str.2681.1.S1_at | 0,897 | SMG1 homolog, phosphatidylinositol 3-kinase-related kinase | smg1 |
| Str.36162.1.A1_at | 0,848 | hypothetical protein LOC100498210 | LOC100498210 |
| Str.31142.1.S1_at | 0,847 | transmembrane protein, adipocyte asscociated 1 | tpra1 |
| Str.14124.1.S2_at | 0,846 | sestrin 1 | sesn1 |
| StrEns.6148.1.S1_a_at | 0,842 | peptide chain release factor 1, mitochondrial-like | LOC100486280 |
| Str.101.1.S1_at | 0,833 | replication factor C (activator 1) 5, 36.5kDa | rfc5 |
| Str.564.1.S1_at | 0,824 | DNZDHHC/NEW1 zinc finger protein 11 | dnz1 |
| Str.33322.1.S1_at | 0,818 | chromosome 16 open reading frame 68 | c16orf68 |
| Str.11519.1.S2_at | 0,815 | spermine oxidase | smox |
| Str.9548.3.A1_at | 0,806 | 28S ribosomal protein S25, mitochondrial-like | LOC100494871 |
| Str.4065.1.S1_at | 0,805 | ATG4 autophagy related 4 homolog A | atg4a |
| Str.27290.1.S1_at | 0,804 | ribonuclease H2, subunit A | rnaseh2a |
| Str.33694.1.S1_at | 0,801 | round spermatid basic protein 1 | rsbn1 |
| Str.2504.2.S1_at | 0,797 | RNA binding motif protein 6 | rbm6 |
| Str.40497.1.S1_at | 0,794 | ras-related C3 botulinum toxin substrate 3 (rho family, small GTP binding protein Rac3) | rac3 |
| Str.20705.2.A1_a_at | 0,789 | uncharacterized protein C8orf41-like | LOC100487242 |
| Str.40433.1.S1_at | 0,789 | vasoactive intestinal polypeptide receptor-like | LOC100497291 |
| Str.8281.1.S1_at | 0,763 | small subunit of serine palmitoyltransferase A-B-like | LOC100495839 |
| Str.27561.1.S1_at | 0,758 | TSPY-like 2 | tspyl2 |
| Str.32322.1.S1_at | 0,755 | nucleosome assembly protein 1-like 4 | nap1l4 |
| Str.31445.1.S1_at | 0,753 | inhibitor of growth family, member 1 | ing1 |
| Str.28870.1.S1_at | 0,752 | Hypothetical protein LOC779512 | LOC779512 |
| Str.27848.1.S1_at | 0,750 | egl nine homolog 3 | egln3 |
| Str.25272.1.S1_at | 0,726 | retinoid X receptor, gamma | rxrg |
| Str.7571.2.S1_a_at | 0,726 | hypothetical LOC100491590 hypothetical LOC100491682 hypothetical LOC100493923 | LOC100491590 LOC100491682 LOC100493923 |
| Str.5120.1.S1_at | 0,719 | adaptor-related protein complex 2, sigma 1 subunit | ap2s1 |
| Str.27693.1.S1_at | 0,701 | SCY1-like 3 | scyl3 |
| Str.10400.2.S1_at | 0,696 | biogenesis of lysosomal organelles complex-1, subunit 1 | bloc1s1 |
| Str.27324.1.S2_at | 0,695 | ras-related protein ras-dva | ras-dva |
| Str.849.2.A1_at | 0,686 | Hypothetical protein MGC75957 | MGC75957 |
| Str.39705.1.S1_s_at | 0,677 | integrator complex subunit 12 | ints12 |
| Str.30835.3.S1_a_at | 0,671 | Interleukin-15 | il-15 |
| Str.2145.1.S1_at | 0,668 | laminin, gamma 1 | lamc1 |
| Str.26914.1.S1_at | 0,666 | UTP11-like, U3 small nucleolar ribonucleoprotein | utp11l |
| Str.13906.1.S1_a_at | 0,657 | low molecular weight neuronal intermediate filament | nif |
| Str.51698.1.S1_at | 0,650 | zinc finger protein 64 homolog | zfp64 |
| Str.29188.1.S1_at | 0,647 | chromosome 1 open reading frame 89 | c1orf89 |
| Str.32445.1.S1_at | 0,644 | tubulin alpha-1D chain-like MGC97820 protein | LOC100487867 MGC97820 |
| Str.3482.1.S1_at | 0,640 | dual specificity phosphatase 22 | dusp22 |
| Str.27186.1.S1_s_at | 0,637 | histone H3.2-like | LOC100496129 |
| Str.44813.1.S1_s_at | 0,632 | oligophrenin 1 | ophn1 |
| Str.27520.1.A1_at | 0,629 | dedicator of cytokinesis 3 | dock3 |
| Str.31215.1.S1_s_at | 0,625 | septin 12 | Sep 12 |
| Str.216.1.S1_at | 0,624 | Hypothetical protein MGC76116 | MGC76116 |
| Str.26671.2.A1_a_at | 0,624 | similar to candidate tumor suppressor OVCA2 | ovca2 |
| Str.18325.1.A1_at | 0,619 | leucine rich repeat neuronal 1 | lrrn1 |
| Str.42318.1.A1_at | 0,611 | GH3 domain-containing protein-like | LOC100488130 |
| Str.22588.1.S1_at | 0,610 | chromosome 11 open reading frame 2 | c11orf2 |
| Str.10971.1.S1_at | 0,609 | OTU domain-containing protein 1-like | LOC100493984 |
| Str.40156.1.S1_at | 0,608 | probable tubulin polyglutamylase TTLL1-like | LOC100495095 |
| Str.37703.1.S1_at | 0,608 | transportin 1 | tnpo1 |
| StrJgi.4435.1.S1_s_at | 0,606 | DnaJ (Hsp40) homolog, subfamily C, member 27 | dnajc27 |
| Str.16298.1.S1_at | 0,602 | nucleotide binding protein-like | nubpl |
| Str.52172.1.S1_at | 0,600 | procollagen-lysine, 2-oxoglutarate 5-dioxygenase 3 | plod3 |
| Str.8414.1.S1_at | 0,590 | nucleoporin 85kDa | nup85 |
| Str.38351.1.S1_at | 0,590 | acyl-CoA synthetase family member 3 | acsf3 |
| Str.42612.1.S1_at | 0,590 | ATPase, H+ transporting, lysosomal 34kDa, V1 subunit D | atp6v1d |
