# Supplementary material for: Brg1 chromatin remodeling ATPase balances germ layer patterning by amplifying the transcriptional burst at midblastula transition
Source: PLoS Genet. 2017 May 12;13(5):e1006757. doi: 10.1371/journal.pgen.1006757 (PMC5428918; doi:10.1371/journal.pgen.1006757)
Supplement: S2 Table — (DOCX) [file pgen.1006757.s012.docx]

Table S2**: genes ≥ 0,59 log_2_ fold (1,5 fold) downregulated upon Brg1 knockdown**

| **Set-probe number** | **fold change [log2]** | **gene name** | **symbol** |
| --- | --- | --- | --- |
| Str.1716.1.S1_x_at | -3,945 | purinergic receptor P2Y, G-protein coupled, 2 | p2ry2 |
| Str.5336.1.S1_at | -2,985 | symplekin | sympk |
| Str.31377.1.S1_at | -2,793 | microsomal triglyceride transfer protein, gene 2 | mttp.2 |
| Str.27013.1.S1_a_at | -2,699 | uroplakin 3B | upk3b |
| Str.11165.7.S1_at | -2,579 | sarcoplasmic/endoplasmic reticulum calcium ATPase 2-like | LOC100489059 |
| Str.4354.1.S1_at | -2,467 | signal transducing adaptor molecule (SH3 domain and ITAM motif) 2 | stam2 |
| StrEns.8369.1.S1_at | -2,460 | growth/differentiation factor 2-like | LOC100485289 |
| Str.13147.1.A1_at | -2,432 | zinc finger protein 219-like | LOC100495520 |
| StrEns.7683.1.S1_at | -2,365 | zona pellucida sperm-binding protein 4-like | LOC100494768 |
| Str.15007.1.S1_at | -2,343 | gastrula-specific protein 17 | gs17 |
| Str.19278.2.S1_at | -2,318 | hypothetical protein LOC100491368 | LOC100491368 |
| Str.5826.1.S1_at | -2,272 | non-metastatic cells 2, protein (NM23B) expressed in | nme2 |
| Str.6718.1.S1_at | -2,204 | hypothetical LOC100494381 | LOC100494381 |
| Str.9193.1.A1_a_at | -2,189 | UPF0632 protein C2orf89-like | LOC100491951 |
| Str.2177.1.A1_a_at | -2,181 | sphingosine-1-phosphate receptor 5 | s1pr5 |
| Str.21595.1.A1_at | -2,172 | cyclin-dependent kinase 5 | cdk5 |
| Str.5015.1.A1_at | -2,168 | neural precursor cell expressed, developmentally down-regulated 9 | nedd9 |
| Str.16322.1.S1_at | -2,131 | Sp9 transcription factor homolog | sp9 |
| Str.31367.1.A1_at | -2,128 | coronin-7-like | LOC100492934 |
| Str.21613.1.S1_a_at | -2,121 | Calponin 2 | cnn2 |
| Str.17625.1.S1_at | -2,107 | hypothetical LOC100495344 | LOC100495344 |
| Str.8191.1.S2_at | -2,082 | phospholipase A2, group XIIB | pla2g12b |
| Str.6681.1.S1_a_at | -2,071 | keratin | krt |
| Str.9958.1.S1_at | -2,051 | chromosome 7 open reading frame 11 | c7orf11 |
| Str.7434.3.S1_at | -2,050 | La ribonucleoprotein domain family, member 1 | larp1 |
| Str.10944.1.S1_at | -2,038 | Zic family member 1 (odd-paired homolog) | zic1 |
| Str.1956.1.S1_at | -2,019 | eukaryotic translation initiation factor 3, subunit H | eif3h |
| Str.11087.1.S1_at | -1,993 | distal-less homeobox 5 | dlx5 |
| Str.15072.2.A1_a_at | -1,975 | delta-like 1 | dll1 |
| Str.40060.1.S1_at | -1,923 | solute carrier family 25 (mitochondrial carrier, phosphate carrier), member 25 | slc25a25 |
| Str.24376.1.S1_at | -1,867 | heart and neural crest derivatives expressed 2 | hand2 |
| Str.1920.1.S2_a_at | -1,864 | hypothetical protein LOC549355 | LOC549355 |
| Str.6151.1.S1_at | -1,826 | ATPase, Na+/K+ transporting, beta 2 polypeptide | atp1b2 |
| Str.10072.1.S2_at | -1,802 | Solute carrier family 2 (facilitated glucose transporter), member 2 | slc2a2 |
| Str.34177.1.A1_s_at | -1,774 | tumor necrosis factor receptor superfamily, member 21 | tnfrsf21 |
| Str.15669.1.S1_at | -1,764 | nuclear receptor subfamily 2, group F, member 1 | nr2f1 |
| Str.1093.1.S1_at | -1,757 | ADP-ribosylation factor-like 4C | arl4c |
| Str.30624.1.S1_at | -1,746 | ephrin-A3 | efna3 |
| Str.20569.1.S1_at | -1,740 | Hypothetical protein LOC100127613 | LOC100127613 |
| Str.8471.1.S1_at | -1,739 | oxysterol binding protein-like 11 | osbpl11 |
| Str.16419.1.S1_at | -1,732 | DNA-damage-inducible transcript 3 | ddit3 |
| Str.643.1.S1_a_at | -1,728 | hypothetical LOC100486597 | LOC100486597 |
| Str.8459.1.S1_at | -1,726 | baculoviral IAP repeat-containing 2 | birc2 |
| Str.4773.2.S1_s_at | -1,716 | hypothetical protein LOC100488209 | LOC100488209 |
| Str.6630.1.S2_at | -1,710 | X-box binding protein 1 | xbp1 |
| Str.29344.1.S1_at | -1,706 | neurogenin 3 | neurog3 |
| Str.26164.1.S1_at | -1,705 | protocadherin 18 | pcdh18 |
| Str.7073.1.S1_at | -1,648 | iroquois homeobox 2 | irx2 |
| Str.11719.1.S1_at | -1,644 | tissue factor-like | LOC100489511 |
| Str.6889.1.A1_at | -1,631 | vav 2 oncogene | LOC733982 |
| Str.11302.1.S1_at | -1,631 | forkhead box D3 | foxd3 |
| Str.16957.1.S2_at | -1,623 | fused in sarcoma | fus |
| Str.20470.1.S2_at | -1,609 | cyclin E2 | ccne2 |
| Str.49050.1.S1_at | -1,590 | Fast skeletal myosin light chain 2 | TNeu107a14.1 |
| StrEns.3882.1.S1_at | -1,583 | forkhead box protein D5-C-like | LOC100485983 |
| Str.21868.1.S1_at | -1,582 | macrophage stimulating 1 receptor (c-met-related tyrosine kinase) | mst1r |
| Str.10727.1.S1_at | -1,579 | deoxyribonuclease gamma-like | LOC100497175 |
| StrEns.9993.1.S1_at | -1,573 | succinate dehydrogenase complex, subunit B, iron sulfur (Ip) | sdhb |
| Str.9762.1.S1_at | -1,571 | stimulated by retinoic acid gene 6 homolog | stra6 |
| Str.10076.1.S1_at | -1,571 | angiomotin like 2 | amotl2 |
| Str.954.1.S1_at | -1,555 | potassium channel tetramerisation domain containing 15 | kctd15 |
| Str.15587.1.S1_at | -1,554 | hypothetical protein LOC100487200 | LOC100487200 |
| Str.2103.1.S1_at | -1,546 | anaphase promoting complex subunit 11 | anapc11 |
| Str.6201.1.S1_at | -1,546 | SRY (sex determining region Y)-box 2 | sox2 |
| Str.10659.1.S1_at | -1,544 | transcription factor AP-2 epsilon (activating enhancer binding protein 2 epsilon) | tfap2e |
| Str.6731.1.S1_at | -1,525 | zinc finger protein 750 | znf750 |
| Str.18694.2.S1_x_at | -1,525 | hypothetical protein LOC100498107 | LOC100498107 |
| Str.3344.1.S1_at | -1,520 | novel protein similar to hatching enzymes | LOC594901 |
| Str.34138.1.A1_at | -1,518 | WD repeat domain 24 | wdr24 |
| Str.14655.1.S2_at | -1,517 | LIM homeobox 5 | lhx5 |
| Str.16688.1.S1_at | -1,513 | RNA pseudouridylate synthase domain containing 3 | rpusd3 |
| Str.12012.1.S1_at | -1,490 | ankyrin repeat domain 10 | ankrd10 |
| Str.29569.1.S1_at | -1,486 | Meningioma (disrupted in balanced translocation) 1, gene 1 | mn1.1 |
| Str.15223.4.S1_a_at | -1,463 | eukaryotic translation initiation factor 4A2 | eif4a2 |
| Str.6185.1.S1_at | -1,463 | cornifelin homolog | cnfn |
| Str.21522.1.S1_at | -1,449 | uroplakin 2 | upk2 |
| Str.49514.1.S1_at | -1,419 | T-box 3 | tbx3 |
| Str.23519.2.S1_a_at | -1,416 | Death inducer-obliterator 1 | dido1 |
| Str.7428.1.S2_at | -1,408 | centromere protein N | cenpn |
| Str.51988.1.S1_at | -1,397 | serine/arginine-rich splicing factor 11 | srsf11 |
| Str.37707.1.A1_at | -1,396 | fibronectin-like | LOC100492420 |
| Str.27213.1.S1_a_at | -1,391 | myeloid/lymphoid or mixed-lineage leukemia (trithorax homolog); translocated to, 4 | mllt4 |
| Str.5401.1.S1_at | -1,381 | retinal pigment epithelium-specific protein 65kDa | rpe65 |
| Str.1047.1.S1_at | -1,379 | prohibitin 2 | phb2 |
| Str.6900.1.S2_s_at | -1,378 | ER degradation enhancer, mannosidase alpha-like 1 /// angiopoietin-related protein 1-like | edem1 /// LOC100491105 |
| Str.39270.1.A1_at | -1,373 | bone morphogenetic protein 15-like | LOC100485893 |
| Str.10293.1.S1_at | -1,363 | SEC22 vesicle trafficking protein-like 2 (S. cerevisiae) | sec22l2 |
| Str.125.1.S1_at | -1,361 | cytochrome b-561 | cyb561 |
| Str.3124.1.S1_at | -1,358 | UbiA prenyltransferase domain containing 1 | ubiad1 |
| Str.7820.2.S1_a_at | -1,358 | immediate early response gene 2 protein-like | LOC100491464 |
| Str.16252.1.S1_at | -1,356 | SRY (sex determining region Y)-box 1 | sox1 |
| Str.44939.1.A1_s_at | -1,354 | RAN binding protein 3 | ranbp3 |
| Str.784.1.A1_at | -1,353 | rap1 GTPase-GDP dissociation stimulator 1-A-like | LOC100486041 |
| Str.21559.2.S1_at | -1,352 | Y box binding protein 2 | ybx2 |
| Str.15005.1.S1_at | -1,349 | hairy and enhancer of split 3, gene 1 | hes3.1 |
| Str.51845.1.A1_at | -1,337 | hypothetical protein MGC69473 | MGC69473 |
| Str.10716.1.S1_at | -1,315 | forkhead box D4-like 1, gene 1 | foxd4l1.1 |
| Str.37993.1.S1_at | -1,315 | uncharacterized protein KIAA0319-like | LOC100492839 |
| Str.14504.1.S1_at | -1,314 | rho GTPase-activating protein 39-like | LOC100485974 |
| Str.27681.1.S1_at | -1,313 | protease, serine 27 | prss27 |
| Str.11402.1.A1_at | -1,298 | protein phosphatase 1, regulatory (inhibitor) subunit 3C, gene 1 | ppp1r3c.1 |
| Str.40874.1.S1_at | -1,293 | ring finger protein 222 | rnf222 |
| Str.15297.1.S1_at | -1,292 | glyoxylate reductase/hydroxypyruvate reductase, gene 2 | grhpr.2 |
| Str.1890.1.S1_at | -1,288 | 39S ribosomal protein L10, mitochondrial-like | LOC100496025 |
| Str.18694.1.S1_at | -1,269 | uncharacterized protein ywlC-like | LOC100497798 |
| Str.3160.2.S1_a_at | -1,256 | serine/arginine-rich splicing factor 6 | srsf6 |
| Str.30747.1.A1_at | -1,254 | regulator of G-protein signaling 16 | rgs16 |
| Str.8142.1.A1_at | -1,245 | TSC22 domain family, member 3 | tsc22d3 |
| Str.3146.2.A1_at | -1,243 | hypothetical LOC100491246 | LOC100491246 |
| Str.11483.1.S1_at | -1,231 | leucine-rich repeat-containing protein 8C-like leucine rich repeat containing 8 family, member C | LOC100488426 lrrc8c |
| Str.18694.2.S1_a_at | -1,221 | uncharacterized protein ywlC-like | LOC100497798 LOC100498107 |
| Str.21593.1.S1_at | -1,209 | H1 histone family, member X | h1fx |
| Str.916.1.S1_at | -1,205 | lin-28 homolog A (C. elegans) | lin28a |
| Str.24820.1.S1_at | -1,202 | regulator of nonsense transcripts 1 | upf1 |
| Str.1440.1.S2_at | -1,190 | solute carrier family 25 (mitochondrial carrier; peroxisomal membrane protein, 34kDa), member 17 | slc25a17 |
| StrJgi.4201.1.S1_s_at | -1,188 | mitogen-activated protein-binding protein-interacting protein-like /// roadblock domain containing 3 | LOC100489368 /// robld3 |
| AFFX-Str-gapdh-M_at | -1,186 | glyceraldehyde-3-phosphate dehydrogenase | gapdh |
| Str.420.1.S1_at | -1,185 | ABO blood group (transferase A, alpha 1-3-N-acetylgalactosaminyltransferase; transferase B, alpha 1-3-galactosyltransferase) | abo |
| Str.38280.1.S1_at | -1,185 | leucine rich repeat containing 24 | lrrc24 |
| Str.15044.1.S1_at | -1,183 | bolA homolog 2 | bola2 |
| Str.17465.2.S1_a_at | -1,181 | hypothetical LOC100493277 | LOC100493277 |
| Str.52067.1.S1_s_at | -1,180 | zinc finger protein 568 | znf568 |
| Str.1183.1.S1_at | -1,180 | transmembrane protein 127 | tmem127 |
| Str.6117.1.S1_at | -1,174 | max interactor 1 | mxi1 |
| Str.7447.2.A1_at | -1,173 | Heterogeneous nuclear ribonucleoprotein D-like | hnrpdl |
| Str.10535.1.S1_at | -1,165 | hypothetical LOC100495377 | LOC100495377 |
| Str.13720.1.S1_at | -1,162 | GTP-binding protein 10 (putative) | gtpbp10 |
| Str.6659.1.S1_at | -1,160 | nerve growth factor receptor | ngfr |
| Str.3502.1.S1_at | -1,158 | roadblock domain containing 3 | robld3 |
| Str.10455.1.S1_at | -1,156 | hypothetical LOC100495538 | LOC100495538 |
| Str.14040.1.A1_at | -1,151 | hypothetical LOC100490704 | LOC100490704 |
| Str.52193.1.S1_at | -1,150 | High-mobility group nucleosomal binding domain 2 | TEgg003n10.1 |
| Str.24811.1.S2_at | -1,148 | myelin expression factor 2 | myef2 |
| Str.9615.1.A1_at | -1,147 | tyrosine kinase, non-receptor, 2 | tnk2 |
| Str.5386.1.S1_at | -1,143 | phosphoglucomutase 1 | pgm1 |
| Str.16319.2.S1_at | -1,138 | hypothetical protein LOC100489354 | LOC100489354 |
| Str.51828.1.S1_a_at | -1,129 | ribosomal protein L9 | rpl9 |
| Str.11288.1.S1_at | -1,128 | hypothetical LOC100486074 | LOC100486074 |
| Str.303.1.S1_at | -1,125 | G protein-coupled receptor, family C, group 5, member C | gprc5c |
| Str.7693.2.S1_at | -1,118 | VENT homeobox 1, gene 1 | ventx1.1 |
| Str.51981.1.S1_s_at | -1,114 | formin binding protein 4 | LOC734003 |
| Str.12750.1.A1_at | -1,107 | LIM homeobox 1 | lhx1 |
| Str.7116.1.S1_at | -1,104 | succinate-CoA ligase, alpha subunit | suclg1 |
| Str.4741.1.S1_at | -1,101 | peroxisomal biogenesis factor 11 beta | pex11b |
| Str.17679.1.S1_at | -1,097 | chromosome 13 open reading frame 15 | c13orf15 |
| Str.10984.1.S1_at | -1,096 | S100 calcium binding protein A11 | s100a11 |
| Str.15740.1.A1_at | -1,093 | MAP kinase interacting serine/threonine kinase 2 | mknk2 |
| Str.17253.1.S1_at | -1,093 | DnaJ (Hsp40) homolog, subfamily C, member 5 gamma | dnajc5g |
| Str.6879.1.A2_at | -1,084 | myeloid cell leukemia sequence 1 (BCL2-related) | mcl1 |
| StrJgi.784.1.S1_s_at | -1,083 | retinoblastoma-like 1 (p107) | rbl1 |
| StrJgi.5287.1.S1_s_at | -1,076 | kin of IRRE like 2 | kirrel2 |
| Str.43062.1.A1_at | -1,074 | hypothetical LOC100135121 | LOC100135121 |
| Str.24440.1.S2_at | -1,052 | transcription factor 7-like 1 (T-cell specific, HMG-box) | tcf7l1 |
| Str.27418.2.A1_at | -1,050 | DENN/MADD domain containing 2C | dennd2c |
| Str.6578.1.S2_a_at | -1,047 | serine/arginine-rich splicing factor 1 | srsf1 |
| Str.2004.1.S1_at | -1,043 | frizzled homolog 2 | fzd2 |
| Str.27441.1.S1_at | -1,041 | dyslexia susceptibility 1 candidate 1 | dyx1c1 |
| Str.8403.1.S1_at | -1,040 | ribosomal RNA processing 12 homolog | rrp12 |
| Str.10058.1.S2_at | -1,035 | syntaxin 19 | stx19 |
| Str.37908.1.S1_a_at | -1,030 | DNA-directed RNA polymerase, mitochondrial-like | LOC100488061 |
| Str.26578.1.S1_a_at | -1,029 | Cbp/p300-interacting transactivator, with Glu/Asp-rich carboxy-terminal domain, 4 | cited4 |
| Str.5673.1.S1_at | -1,028 | high density lipoprotein binding protein (vigilin) | hdlbp |
| Str.10008.1.S1_at | -1,022 | hypothetical protein LOC100487199 | LOC100487199 |
| Str.10236.1.S1_at | -1,021 | receptor-interacting serine-threonine kinase 4 | ripk4 |
| Str.10362.1.A1_at | -1,018 | hypothetical LOC100493109 | LOC100493109 |
| Str.37633.1.S1_at | -1,015 | aminopeptidase-like 1 | npepl1 |
| Str.2049.3.S1_a_at | -1,007 | solute carrier family 25 (mitochondrial carrier; phosphate carrier), member 3 | slc25a3 |
| Str.28803.2.S1_a_at | -1,005 | hypothetical LOC100486098 | LOC100486098 |
| Str.1861.3.S1_s_at | -1,005 | DEAD (Asp-Glu-Ala-Asp) box polypeptide 6 | ddx6 |
| Str.20062.1.S1_at | -0,997 | phosphoglycerate dehydrogenase | phgdh |
| Str.52227.2.A1_a_at | -0,993 | hypothetical protein | LOC100484994 LOC100486020 LOC100489931 LOC100490293 LOC100492588 LOC100498560 |
| Str.22118.1.S1_at | -0,992 | hypothetical protein LOC100038295 | LOC100038295 |
| Str.1881.1.S1_at | -0,990 | hypothetical protein LOC100497165 | LOC100497165 |
| Str.1933.1.S1_at | -0,990 | mitochondrial ribosomal protein L12 | mrpl12 |
| Str.6229.1.A1_at | -0,989 | POU class V protein oct-25 | Oct25 |
| Str.22855.1.S1_at | -0,988 | pleckstrin homology-like domain, family A, member 1 | phlda1 |
| Str.15274.1.S1_at | -0,985 | Meis homeobox 3 | meis3 |
| Str.11630.1.A1_s_at | -0,985 | XPA binding protein 2 | xab2 |
| Str.6222.1.S1_at | -0,984 | annexin A2 | anxa2 |
| Str.79.2.S1_at | -0,984 | POU domain, class 5, transcription factor 1.2-like | LOC100498076 |
| Str.20248.1.S1_at | -0,980 | sema domain, immunoglobulin domain (Ig), short basic domain, secreted, (semaphorin) 3F | sema3f |
| Str.10351.1.S1_at | -0,977 | G protein-coupled receptor 161 | gpr161 |
| Str.21546.1.S1_at | -0,976 | chromosome 14 open reading frame 179 | c14orf179 |
| Str.8453.1.S1_at | -0,975 | chromatin modifying protein 1A | chmp1a |
| Str.6874.1.S1_at | -0,974 | RAB11A, member RAS oncogene family | rab11a |
| Str.3045.1.S1_at | -0,972 | musashi homolog 1 | msi1 |
| Str.7693.2.S1_a_at | -0,967 | VENT homeobox 1, gene 1 VENT homeobox 1, gene 2 | ventx1.1 ventx1.2 |
| Str.9076.1.S1_at | -0,967 | mitochondrial ribosomal protein S2 | mrps2 |
| Str.3002.1.A1_at | -0,965 | Hypothetical protein LOC779546 | LOC779546 |
| Str.37570.1.S1_at | -0,964 | solute carrier family 7 (cationic amino acid transporter, y+ system), member 3 | slc7a3 |
| Str.51678.1.S1_at | -0,962 | ribosomal protein S17 | rps17 |
| Str.6544.1.S1_at | -0,961 | DEP domain containing 7 | depdc7 |
| Str.51323.1.S1_at | -0,960 | hairy and enhancer of split 2 | hes2 |
| Str.7709.1.S2_at | -0,957 | RCD1 required for cell differentiation1 homolog | rqcd1 |
| Str.37157.1.S1_at | -0,956 | phosphatidylinositol 4-kinase type 2 alpha | pi4k2a |
| StrEns.4639.1.S1_s_at | -0,951 | arachidonate 5-lipoxygenase-activating protein | alox5ap |
| Str.17801.1.A1_at | -0,951 | hypothetical protein LOC100488783 | LOC100488783 |
| Str.7111.1.S1_at | -0,949 | nucleophosmin/nucleoplasmin 2 | npm2 |
| Str.27139.1.S1_at | -0,945 | PR domain containing 1, with ZNF domain | prdm1 |
| Str.37891.2.S1_at | -0,939 | hypothetical protein LOC100498385 | LOC100498385 |
| Str.7002.1.S1_at | -0,935 | hypothetical LOC100497373 | LOC100497373 |
| StrJgi.754.1.S1_s_at | -0,931 | G2/M-phase specific E3 ubiquitin protein ligase | g2e3 |
| Str.19642.1.S1_at | -0,923 | ATG16 autophagy related 16-like 1 | atg16l1 |
| Str.51307.2.S1_s_at | -0,922 | hypothetical protein LOC100485101 | LOC100485101 |
| Str.5058.1.S1_at | -0,916 | rhabdoid tumor deletion region gene 1 | rtdr1 |
| Str.6021.1.S1_at | -0,915 | hypothetical protein MGC75753 | MGC75753 |
| StrJgi.6490.1.S1_s_at | -0,913 | nischarin | nisch |
| Str.27191.2.S1_at | -0,912 | putative ferric-chelate reductase 1-like | LOC100489839 |
| Str.16697.1.S1_at | -0,912 | nuclear receptor subfamily 1, group D, member 1 | nr1d1 |
| Str.10814.1.S1_a_at | -0,910 | T, brachyury homolog | t |
| Str.15841.2.S1_at | -0,905 | ras homolog gene family, member V | rhov |
| Str.10791.1.S1_at | -0,901 | F-box protein 5 | fbxo5 |
| Str.29354.1.A1_at | -0,896 | integrator complex subunit 4-like | LOC100498633 |
| Str.4240.1.S1_at | -0,896 | catechol-O-methyltransferase | comt |
| Str.37794.1.S1_at | -0,895 | lysophosphatidic acid receptor 6 | lpar6 |
| Str.8913.1.S3_at | -0,890 | C-terminal binding protein 2 | ctbp2 |
| Str.5871.1.S1_at | -0,888 | cyclin-dependent kinase inhibitor xic1 | cdknx |
| Str.11206.1.S1_a_at | -0,885 | hypothetical LOC100496939 | LOC100496939 |
| Str.42508.1.A1_at | -0,882 | v-kit Hardy-Zuckerman 4 feline sarcoma viral oncogene homolog | kit |
| Str.6167.1.S1_at | -0,880 | phosphorylase, glycogen, muscle | pygm |
| Str.41326.1.S1_s_at | -0,875 | hairy and enhancer of split 6, gene 1 | hes6.1 |
| StrEns.3083.1.S1_s_at | -0,873 | angel homolog 2 | angel2 |
| Str.27368.1.S1_at | -0,864 | HCLS1-associated protein X-1-like | LOC100485912 |
| Str.42404.1.A1_at | -0,864 | Smg-5 homolog, nonsense mediated mRNA decay factor | smg5 |
| Str.10780.1.S1_at | -0,860 | ATP synthase, H+ transporting, mitochondrial F1 complex, delta subunit | atp5d |
| Str.50368.2.S1_at | -0,858 | retinoic acid receptor gamma-A-like | LOC100485387 |
| StrEns.6535.1.S1_s_at | -0,857 | hormonally up-regulated Neu-associated kinase | hunk |
| StrAffx.22.1.S1_at | -0,855 | hypothetical protein LOC100486038 /// hypothetical protein LOC100487146 | LOC100486038 /// LOC100487146 |
| Str.6401.1.S1_a_at | -0,854 | cullin 4B | cul4b |
| Str.292.1.S1_at | -0,853 | KIAA0907 | kiaa0907 |
| Str.4669.1.S1_at | -0,851 | hypothetical protein LOC100494135 | LOC100494135 |
| Str.4856.1.S1_at | -0,846 | folate receptor 1 (adult) | folr1 |
| Str.538.2.A1_at | -0,844 | chordin | chrd |
| StrJgi.4129.1.S1_at | -0,844 | ATPase inhibitory factor 1 | atpif1 |
| Str.169.2.S1_a_at | -0,840 | sal-like 1 | sall1 |
| Str.24427.1.A1_at | -0,838 | ran-binding protein 3-like | LOC100498481 |
| Str.14235.1.S1_at | -0,835 | coenzyme Q10 homolog B | coq10b |
| Str.16683.1.A1_at | -0,834 | phosphatidylinositol 4-kinase, catalytic, alpha | pi4ka |
| Str.19092.2.A1_at | -0,830 | RAB11 family interacting protein 1 (class I) | rab11fip1 |
| Str.511.2.S1_at | -0,829 | activating transcription factor 5 | atf5 |
| Str.31799.1.S1_at | -0,829 | hypothetical protein LOC100124772 | LOC100124772 |
| Str.38847.3.S1_at | -0,827 | chromosome 19 open reading frame 66 | c19orf66 |
| Str.31289.2.S1_at | -0,825 | oocyte zinc finger protein XlCOF7.1-like | LOC100496553 |
| Str.50363.2.S1_at | -0,825 | COX10 homolog, cytochrome c oxidase assembly protein, heme A: farnesyltransferase | cox10 |
| Str.10916.1.S1_at | -0,823 | NADH dehydrogenase (ubiquinone) 1 beta subcomplex 4 | ndufb4 |
| Str.11181.1.S1_at | -0,823 | transmembrane protein 49 | tmem49 |
| Str.471.1.S1_a_at | -0,822 | ubiquitin-conjugating enzyme E2I (UBC9 homolog) | ube2i |
| Str.15327.1.S1_at | -0,821 | chromosome 3 open reading frame 32 | c3orf32 |
| Str.37502.1.S1_at | -0,820 | lipase A, lysosomal acid, cholesterol esterase | lipa |
| Str.10764.1.S1_a_at | -0,811 | transmembrane protein 183A | tmem183a |
| Str.27547.1.S3_at | -0,810 | hypothetical protein LOC550042 | LOC550042 |
| Str.24913.1.S1_at | -0,810 | CLPTM1-like | clptm1l |
| Str.488.1.S1_at | -0,802 | hypothetical protein MGC76328 | MGC76328 |
| Str.3639.2.S1_s_at | -0,797 | ankyrin repeat domain 11 | ankrd11 |
| Str.26831.1.S1_s_at | -0,791 | bone morphogenetic protein 4 | bmp4 |
| Str.4537.1.S1_at | -0,788 | alpha-ketoglutarate-dependent dioxygenase alkB homolog 3-like | LOC100495476 |
| Str.8680.1.S1_at | -0,786 | lactate dehydrogenase B | ldhb |
| Str.26963.1.S1_at | -0,782 | Hypothetical protein LOC100145127 | LOC100145127 |
| Str.3361.1.S1_at | -0,780 | MAK16 homolog | mak16 |
| Str.4965.1.S1_at | -0,777 | minichromosome maintenance complex component 6 | mcm6.2 |
| StrEns.171.1.S1_s_at | -0,776 | phosphatidic acid phosphatase type 2B | ppap2b |
| Str.6623.1.S1_at | -0,774 | hypothetical protein LOC733945 | LOC733945 |
| Str.6018.1.S1_at | -0,774 | hairy and enhancer of split 4 | hes4 |
| Str.15770.1.S1_at | -0,773 | prostaglandin-endoperoxide synthase 2 | ptgs2 |
| Str.15499.1.S1_at | -0,772 | oxysterol binding protein | osbp |
| Str.24757.1.S1_at | -0,764 | nipsnap homolog 3A | nipsnap3a |
| Str.1347.1.A1_at | -0,761 | integrin-linked kinase | ilk |
| Str.24893.1.S1_at | -0,760 | SPRY domain-containing SOCS box protein 4-like | LOC100496912 |
| Str.10257.1.S1_at | -0,756 | 28S ribosomal protein S18b, mitochondrial-like | LOC100489063 |
| Str.10270.1.S1_at | -0,755 | hydroxysteroid (17-beta) dehydrogenase 12 | hsd17b12 |
| StrEns.9037.1.S1_s_at | -0,755 | WD repeat domain 91 | wdr91 |
| Str.16160.1.S1_at | -0,755 | forkhead box I4, gene 2 | foxi4.2 |
| Str.21592.1.S1_at | -0,754 | Rho/Rac guanine nucleotide exchange factor (GEF) 2 | arhgef2 |
| Str.1370.1.S1_s_at | -0,752 | zinc finger, HIT-type containing 1 | znhit1 |
| Str.51820.1.S1_at | -0,749 | cyclin F | ccnf |
| Str.34332.1.A1_s_at | -0,744 | heme oxygenase (decycling) 2 | hmox2 |
| Str.2761.1.S1_at | -0,739 | CD81 molecule | cd81 |
| Str.49437.1.S1_at | -0,737 | Hypothetical protein LOC100144964 | LOC100144964 |
| Str.10129.1.S1_at | -0,736 | Novel solute carrier family 18 (vesicular monoamine) slca18 protein | LOC733714 |
| Str.43415.1.S1_at | -0,731 | Coiled-coil domain containing 92 | ccdc92 |
| Str.10123.1.S1_at | -0,731 | glucose-fructose oxidoreductase domain containing 1 | gfod1 |
| Str.16503.1.S1_at | -0,729 | adaptor-related protein complex 2, alpha 2 subunit | ap2a2 |
| StrEns.6699.1.S1_at | -0,729 | tRNA pseudouridine synthase-like 1-like | LOC100497905 |
| Str.46269.1.A1_s_at | -0,728 | chromosome 19 open reading frame 60 | c19orf60 |
| Str.19440.1.S1_a_at | -0,728 | B-cell translocation gene 5 | btg5 |
| Str.381.2.A1_a_at | -0,727 | ubiquilin 4 | ubqln4 |
| Str.34312.1.A1_at | -0,725 | hypothetical protein LOC100492765 | LOC100492765 |
| Str.50958.1.S1_s_at | -0,721 | THAP domain containing 4 | thap4 |
| Str.2540.1.S1_at | -0,714 | SMAD family member 6 | smad6 |
| Str.22075.1.S1_at | -0,711 | mitochondrial ribosomal protein L33 | mrpl33 |
| Str.37581.1.S1_at | -0,708 | hypothetical protein LOC100489670 | LOC100489670 |
| Str.31764.1.A1_at | -0,706 | neurofilament, light polypeptide | nefl |
| Str.5440.1.S1_at | -0,705 | ariadne homolog, ubiquitin-conjugating enzyme E2 binding protein, 1 | arih1 |
| Str.49709.1.A1_s_at | -0,704 | intraflagellar transport 52 homolog | ift52 |
| Str.6196.1.S2_at | -0,704 | hypothetical protein MGC75626 | MGC75626 |
| Str.11467.1.S1_at | -0,703 | ferredoxin reductase | fdxr |
| Str.34173.3.S1_s_at | -0,703 | chromosome 2 open reading frame 42 | c2orf42 |
| Str.27369.1.S2_at | -0,702 | homeobox B3 | hoxb3 |
| Str.6282.1.A1_x_at | -0,702 | filamin C, gamma | flnc |
| Str.18831.1.S1_at | -0,701 | zinc finger protein 567-like | LOC100495895 |
| Str.563.1.S1_at | -0,700 | inositol-3-phosphate synthase 1 | isyna1 |
| Str.10174.1.A1_at | -0,698 | single-strand-selective monofunctional uracil-DNA glycosylase 1 | smug1 |
| Str.3944.1.S1_at | -0,695 | hypothetical protein LOC733539 | LOC733539 |
| StrEns.1418.1.S1_s_at | -0,695 | Bloom syndrome, RecQ helicase-like | blm |
| Str.10015.1.S1_at | -0,694 | transmembrane protein 41B | tmem41b |
| Str.27247.1.S2_s_at | -0,692 | serine peptidase inhibitor, Kunitz type, 2 | spint2 |
| Str.29917.1.A1_at | -0,689 | HMG box-containing protein | hbcx |
| Str.7564.2.S1_a_at | -0,689 | diazepam binding inhibitor (dbi) | dbi |
| Str.9089.2.S1_a_at | -0,688 | trans-2,3-enoyl-CoA reductase | tecr |
| Str.15911.1.A1_at | -0,688 | hypothetical protein MGC145685 | MGC145685 |
| Str.6712.1.S1_s_at | -0,681 | ribosomal protein S2 | rps2 |
| Str.6262.1.A1_at | -0,681 | phosphatase and actin regulator 4 | phactr4 |
| Str.51838.1.S1_at | -0,680 | leucine-rich repeats and WD repeat domain containing 1 | lrwd1 |
| Str.20309.1.A1_at | -0,677 | cyclin-dependent kinase 6 | cdk6 |
| StrAffx.136.1.S1_s_at | -0,674 | family with sequence similarity 160, member B2 | fam160b2 |
| Str.24290.1.S1_at | -0,674 | hypothetical protein MGC147490 | MGC147490 |
| Str.32043.1.A1_at | -0,670 | hypothetical protein MGC147507 | MGC147507 |
| Str.2514.1.S1_a_at | -0,668 | NIMA (never in mitosis gene a)-related kinase 2 | nek2 |
| Str.21553.1.S1_at | -0,667 | zinc finger protein 740 | znf740 |
| Str.862.1.S1_at | -0,664 | transcription factor CP2 | tfcp2 |
| Str.1699.1.S1_at | -0,664 | malate dehydrogenase 2, NAD (mitochondrial) | mdh2 |
| StrJgi.162.1.S1_at | -0,663 | serine palmitoyltransferase, long chain base subunit 3 | sptlc3 |
| Str.15129.1.S1_at | -0,660 | alkB, alkylation repair homolog 5 | alkbh5 |
| Str.8533.1.A1_s_at | -0,655 | caveolin 2 | cav2 |
| Str.7025.2.S2_at | -0,654 | hypothetical LOC100496454 | LOC100496454 |
| Str.5456.2.A1_s_at | -0,653 | arsA arsenite transporter, ATP-binding, homolog 1 (bacterial) | asna1 |
| Str.28989.1.A1_at | -0,651 | 72 kDa inositol polyphosphate 5-phosphatase-like | LOC100486470 |
| Str.7586.3.S1_a_at | -0,650 | MAPK scaffold protein 1 | mapksp1 |
| Str.6355.1.S1_at | -0,648 | RAD51 associated protein 1 | rad51ap1 |
| Str.10998.1.S1_at | -0,644 | CNDP dipeptidase 2 (metallopeptidase M20 family) | cndp2 |
| Str.464.1.S1_at | -0,643 | radial spoke head 9 homolog | rsph9 |
| Str.11870.1.S1_at | -0,643 | collagen, type IX, alpha 3 | col9a3 |
| Str.17125.1.S1_at | -0,643 | Rho family GTPase 3 | rnd3 |
| Str.17056.1.S1_a_at | -0,643 | H3 histone, family 3B (H3.3B) | h3f3b |
| Str.28075.1.S1_s_at | -0,642 | translocator protein (18kDa) | tspo |
| Str.8911.1.S1_at | -0,642 | hippocampus abundant transcript 1 | hiat1 |
| Str.5375.2.S1_a_at | -0,640 | uridine monophosphate synthetase | umps |
| Str.3.1.S1_a_at | -0,633 | nanos homolog 1 | nanos1 |
| Str.24671.1.S1_a_at | -0,633 | tubulin tyrosine ligase-like family, member 4 | ttll4 |
| Str.11129.1.S1_at | -0,631 | ubiquitin-conjugating enzyme E2B (RAD6 homolog) | ube2b |
| Str.26863.1.S1_at | -0,631 | mitochondrial ribosomal protein L40 | mrpl40 |
| Str.8957.1.A1_at | -0,630 | ATG2 autophagy related 2 homolog A | atg2a |
| Str.21719.1.S1_at | -0,627 | mitochondrial ribosomal protein L52 | mrpl52 |
| Str.6511.1.S1_at | -0,624 | transmembrane protein 11 | tmem11 |
| Str.52134.1.S1_s_at | -0,620 | PQ loop repeat containing 3 | pqlc3 |
| Str.31657.1.S1_at | -0,619 | hypothetical protein LOC100494403 | LOC100494403 |
| Str.10064.2.S1_a_at | -0,618 | MTERF domain containing 1 | mterfd1 |
| StrJgi.4070.1.S1_s_at | -0,616 | glycoprotein-N-acetylgalactosamine 3-beta-galactosyltransferase 1-like | LOC100487794 |
| Str.15041.1.S1_at | -0,616 | regulator of chromosome condensation (RCC1) and BTB (POZ) domain containing protein 2 | rcbtb2 |
| Str.8025.1.S1_a_at | -0,615 | keratin 18 | krt18 |
| Str.16910.1.S1_at | -0,615 | activating transcription factor 4 (tax-responsive enhancer element B67) | atf4 |
| Str.1806.1.S1_at | -0,614 | Hypothetical protein MGC146711 | MGC146711 |
| Str.37447.1.S1_at | -0,613 | hypothetical protein LOC100487171 | LOC100487171 |
| Str.4820.1.S2_at | -0,610 | eukaryotic translation elongation factor 1 alpha 1, oocyte form | eef1a1o |
| Str.21822.1.S1_at | -0,610 | AFG3 ATPase family gene 3-like 2 (S. cerevisiae) | afg3l2 |
| Str.2054.1.S1_at | -0,610 | YTH domain family, member 1 | ythdf1 |
| Str.6670.1.S1_at | -0,609 | aldolase A, fructose-bisphosphate | aldoa |
| Str.11968.1.A1_at | -0,606 | RAS, dexamethasone-induced 1 | rasd1 |
| Str.6981.1.S1_at | -0,606 | growth arrest and DNA-damage-inducible, gamma | gadd45g |
| Str.7658.1.S1_at | -0,603 | maternal embryonic leucine zipper kinase | melk |
| Str.2080.1.S1_at | -0,601 | ribonuclease P 14kDa subunit | rpp14 |
| Str.27246.1.S1_at | -0,600 | SRY (sex determining region Y)-box 11 | sox11 |
| Str.1379.1.S1_at | -0,598 | cell division cycle associated 7 | cdca7 |
| StrEns.9103.1.S1_at | -0,598 | zinc finger homeobox protein 3-like | LOC100489378 |
| StrEns.8056.1.S1_at | -0,598 | solute carrier family 45 member 3-like | LOC100496642 |
| Str.52196.1.S1_at | -0,598 | enolase-phosphatase 1 | enoph1 |
| Str.10330.1.S2_at | -0,596 | sprouty homolog 1, antagonist of FGF signaling | spry1 |
| AFFX-Str-ef1a-5_at | -0,594 | eukaryotic translation elongation factor 1 alpha 1 | eef1a1 |
| Str.27580.1.S1_at | -0,594 | cat eye syndrome chromosome region, candidate 5 homolog (human) | cecr5 |
| Str.5136.1.S1_at | -0,592 | TGFB-induced factor homeobox 1 | tgif1 |
