# Supplementary material for: Brg1 chromatin remodeling ATPase balances germ layer patterning by amplifying the transcriptional burst at midblastula transition
Source: PLoS Genet. 2017 May 12;13(5):e1006757. doi: 10.1371/journal.pgen.1006757 (PMC5428918; doi:10.1371/journal.pgen.1006757)
Supplement: S3 Table — (DOCX) [file pgen.1006757.s013.docx]

Table S3: **Morphological rescue of Brg1 knockdown with hBrg1, hBrm and xISWI**

| **DMZ-injections** | **total number** | **normal/ rescued** | **head and eye defect** | **gastrulation defects** |
| --- | --- | --- | --- | --- |
| Uninjected | 24 | 24 | 0 | 0 |
| CMO 40ng/emb | 17 | 17 | 0 | 0 |
| BMO 10ng/emb | 75 | 0 | 64 | 11 |
| BMO 10ng/emb + 500 pg/emb hBrm mRNA | 24 | 0 | 22 | 2 |
| BMO 10ng/emb + 500 pg/emb xISWI mRNA | 38 | 0 | 17 | 21 (with head and eye defect) |
| BMO 10ng/emb + 500 pg hBrg1 mRNA/emb | 45 | 34 | 7 | 4 |
