# Supplementary material for: Brg1 chromatin remodeling ATPase balances germ layer patterning by amplifying the transcriptional burst at midblastula transition
Source: PLoS Genet. 2017 May 12;13(5):e1006757. doi: 10.1371/journal.pgen.1006757 (PMC5428918; doi:10.1371/journal.pgen.1006757)
Supplement: S4 Table — (DOCX) [file pgen.1006757.s014.docx]

Table S4: **genes ≥ 2,5 log_2_ fold (5,6 fold) upregulated after MBT**

| **Set-probe number** | **fold change [log2]** | **gene name** | **symbol** |
| --- | --- | --- | --- |
| Str.14985.1.S1_at | 11,53 | eomesodermin | eomes |
| Str.20300.1.S1_a_at | 11,38 | mix-like endodermal regulator | mixer |
| Str.1534.1.S1_at | 11,09 | hypothetical LOC100487344 | LOC100487344 |
| Str.6030.1.S1_at | 11,09 | forkhead box A4 | foxa4 |
| Str.15007.1.S1_at | 10,74 | gastrula-specific protein 17 | gs17 |
| Str.10688.1.S1_at | 10,28 | hypothetical protein LOC100170595 putative nuclease HARBI1-like | LOC100170595 LOC100492963 |
| Str.37794.1.S1_at | 10,24 | lysophosphatidic acid receptor 6 | lpar6 |
| Str.112.1.S1_at | 10,12 | Brachyury-inducible homeobox 1, gene 1 homeobox protein Mix.1-like | bix1.1 LOC100491743 |
| Str.10234.1.S2_at | 10,10 | Zic family member 3 (odd-paired homolog) | zic3 |
| Str.10066.1.S1_at | 10,07 | mesendoderm nuclear factor, gene 1 | menf.1 |
| Str.113.1.S1_at | 10,02 | mix1 homeobox | mix1 |
| Str.30719.1.A1_s_at | 9,97 | ras homolog gene family, member V | rhov |
| Str.11884.1.S1_at | 9,86 | anti-dorsalizing morphogenic protein | admp |
| Str.17224.1.S1_at | 9,83 | Zic family member 1 (odd-paired homolog) | zic1 |
| Str.10058.1.S1_at | 9,62 | syntaxin 19 | stx19 |
| Str.6151.1.S1_at | 9,50 | ATPase, Na+/K+ transporting, beta 2 polypeptide | atp1b2 |
| Str.447.1.S1_at | 9,48 | ATPase, H+/K+ transporting, nongastric, alpha polypeptide | atp12a |
| Str.41254.1.S1_s_at | 9,47 | SRY (sex determining region Y)-box 2 | sox2 |
| Str.6141.1.S1_a_at | 9,39 | glycine amidinotransferase (L-arginine:glycine amidinotransferase) | gatm |
| Str.7025.2.S2_at | 9,36 | hypothetical LOC100496454 | LOC100496454 |
| StrJgi.4169.1.S1_s_at | 9,32 | hypothetical protein LOC100488209 | LOC100488209 |
| Str.7482.2.S1_a_at | 9,31 | protocadherin 8, gene 2 | pcdh8.2 |
| Str.4932.1.S1_a_at | 9,17 | hypothetical protein LOC100494704 VENT homeobox 2, gene 1 | LOC100494704 ventx2.1 |
| Str.15097.1.S1_a_at | 9,15 | hypothetical protein hypothetical protein | LOC100497333 LOC100498266 |
| Str.538.2.A2_at | 9,14 | chordin | chrd |
| Str.6229.2.S1_at | 9,12 | ephrin-B2 | efnb2 |
| Str.2980.2.A1_a_at | 9,05 | transcription factor AP-2 alpha (activating enhancer binding protein 2 alpha) | tfap2a |
| Str.6718.1.S1_at | 8,97 | hypothetical LOC100494381 | LOC100494381 |
| Str.11206.1.S1_a_at | 8,93 | hypothetical LOC100496939 | LOC100496939 |
| Str.20278.2.A1_a_at | 8,90 | hypothetical protein LOC100489209 | LOC100489209 |
| StrJgi.7797.1.S1_s_at | 8,84 | hyaluronan synthase 2 | has2 |
| Str.15369.1.S1_at | 8,76 | hypothetical protein LOC100127584 | LOC100127584 |
| Str.10804.1.S2_s_at | 8,76 | keratin 5, gene 7 | krt5.7 |
| Str.79.2.S1_at | 8,75 | POU domain, class 5, transcription factor 1.2-like | LOC100498076 |
| Str.114.1.S1_at | 8,74 | SRY (sex determining region Y)-box 17 alpha | sox17a |
| Str.1955.1.S1_at | 8,73 | hypothetical protein LOC100491352 | LOC100491352 |
| Str.20147.1.S1_at | 8,73 | fibroblast growth factor 8 (androgen-induced) | fgf8 |
| Str.42150.1.S1_at | 8,64 | novel zinc finger protein | LOC733912 |
| Str.10814.1.S1_a_at | 8,56 | T, brachyury homolog | t |
| Str.7018.1.S1_at | 8,56 | frizzled-related protein | frzb |
| Str.10716.1.S1_at | 8,55 | forkhead box D4-like 1, gene 1 | foxd4l1.1 |
| Str.4900.1.S1_at | 8,53 | zinc finger protein 470 | znf470 |
| Str.51679.2.S3_a_at | 8,52 | hypothetical protein LOC100498064 | LOC100498064 |
| StrJgi.754.1.S1_s_at | 8,48 | G2/M-phase specific E3 ubiquitin protein ligase | g2e3 |
| Str.51679.2.S1_a_at | 8,41 | hypothetical protein LOC100498497 | LOC100498497 |
| Str.14655.1.S2_at | 8,39 | LIM homeobox 5 | lhx5 |
| Str.7693.2.S1_at | 8,37 | VENT homeobox 1, gene 1 | ventx1.1 |
| Str.52227.2.A1_a_at | 8,31 | hypothetical LOC100484994 hypothetical LOC100486020 hypothetical LOC100489931 hypothetical LOC100490293 hypothetical LOC100492588 hypothetical LOC100498560 | LOC100484994 LOC100486020 LOC100489931 LOC100490293 LOC100492588 LOC100498560 |
| Str.7447.2.A1_at | 8,30 | Heterogeneous nuclear ribonucleoprotein D-like | hnrpdl |
| Str.15963.1.S1_at | 8,29 | tyrosine aminotransferase | tat |
| Str.7571.1.S1_a_at | 8,29 | hypothetical LOC100491682 | LOC100491682 |
| Str.15754.1.S1_s_at | 8,26 | fibronectin leucine rich transmembrane protein 3 | flrt3 |
| Str.2944.1.S1_at | 8,26 | cone-rod homeobox | crx |
| Str.490.1.S2_at | 8,20 | bone morphogenetic protein 4 | bmp4 |
| Str.2572.2.S1_x_at | 8,20 | hypothetical protein LOC100487243 | LOC100487243 |
| Str.14828.1.S1_at | 8,18 | left-right determination factor | lefty |
| Str.7571.2.S1_a_at | 8,16 | hypothetical LOC100491590 hypothetical LOC100491682 hypothetical LOC100493923 | LOC100491590 LOC100491682 LOC100493923 |
| Str.31317.1.S1_at | 8,14 | hypothetical protein LOC100487186 | LOC100487186 |
| Str.6731.1.S1_at | 8,14 | zinc finger protein 750 | znf750 |
| Str.51845.1.A1_at | 8,00 | hypothetical protein MGC69473 | MGC69473 |
| Str.1534.1.S2_a_at | 7,96 | hypothetical protein LOC100495042 | LOC100495042 |
| Str.2572.4.A1_a_at | 7,94 | hypothetical LOC100488588 | LOC100488588 |
| Str.10455.1.S1_at | 7,86 | hypothetical LOC100495538 | LOC100495538 |
| Str.7017.1.S1_at | 7,86 | SRY (sex determining region Y)-box 17 beta, gene 2 | sox17b.2 |
| Str.15027.1.S1_at | 7,85 | wingless-type MMTV integration site family, member 8A | wnt8a |
| Str.1702.1.S1_at | 7,78 | secreted frizzled-related protein 2 | sfrp2 |
| Str.52227.1.S1_a_at | 7,76 | hypothetical protein LOC100492588 | LOC100492588 |
| Str.17465.2.S1_a_at | 7,67 | hypothetical LOC100493277 | LOC100493277 |
| Str.3072.1.S1_at | 7,65 | grainyhead-like 3 | grhl3 |
| Str.6681.1.S1_a_at | 7,60 | keratin | krt |
| Str.6282.1.A1_x_at | 7,59 | filamin C, gamma | flnc |
| Str.6185.1.S1_at | 7,58 | cornifelin homolog | cnfn |
| Str.7693.2.S1_a_at | 7,50 | VENT homeobox 1, gene 1 VENT homeobox 1, gene 2 | ventx1.1 ventx1.2 |
| Str.740.1.S1_at | 7,44 | chromosome 3 open reading frame 54 | c3orf54 |
| Str.10337.1.S1_at | 7,37 | dual specificity phosphatase 6 | dusp6 |
| Str.27989.2.A1_s_at | 7,34 | piggyBac transposable element-derived protein 4-like | LOC100494255 |
| Str.21559.2.S1_x_at | 7,25 | Y box binding protein 2 | ybx2 |
| Str.30372.1.S1_x_at | 7,15 | Nucleolar protein 5A (56kDa with KKE/D repeat) | TEgg001j23.1 |
| Str.27186.1.S1_at | 7,15 | histone cluster 2, H3c | hist2h3c |
| Str.36384.1.S1_at | 7,15 | nodal homolog 2-A-like | LOC100491883 |
| Str.6900.1.S2_s_at | 7,14 | ER degradation enhancer, mannosidase alpha-like 1 angiopoietin-related protein 1-like | edem1 LOC100491105 |
| Str.10803.1.S1_at | 7,13 | goosecoid homeobox | gsc |
| Str.15.1.S1_at | 7,13 | cell division cycle 25 homolog B | cdc25b |
| Str.49496.1.S1_s_at | 7,11 | apelin receptor | aplnr |
| Str.27467.1.S1_at | 7,11 | zinc finger protein 350 | znf350 |
| Str.10021.1.S1_at | 7,11 | orthodenticle homeobox 2 | otx2 |
| Str.11939.1.S1_at | 7,06 | l-amino-acid oxidase-like | LOC100496394 |
| Str.30175.1.S1_at | 7,04 | VENT homeobox 3, gene 2 | ventx3.2 |
| Str.6981.1.S1_at | 7,04 | growth arrest and DNA-damage-inducible, gamma | gadd45g |
| Str.8142.1.A1_at | 7,03 | TSC22 domain family, member 3 | tsc22d3 |
| Str.23268.1.A1_at | 6,99 | peptidase M20 domain containing 1 | pm20d1 |
| Str.37312.1.S1_x_at | 6,95 | hypothetical protein LOC100145106 | LOC100145106 |
| Str.10236.1.S1_at | 6,94 | receptor-interacting serine-threonine kinase 4 | ripk4 |
| Str.7735.3.S1_a_at | 6,91 | nodal homolog 3-B-like nodal homolog nodal homolog 3, gene 2 | LOC100491713 nodal nodal3.2 |
| Str.25552.1.S2_at | 6,90 | lysophosphatidic acid receptor 2 | lpar2 |
| Str.7693.1.S1_at | 6,79 | VENT homeobox 1, gene 2 | ventx1.2 |
| Str.7073.1.S1_at | 6,79 | iroquois homeobox 2 | irx2 |
| Str.16160.1.S1_at | 6,79 | forkhead box I4, gene 2 | foxi4.2 |
| Str.8390.1.S2_at | 6,75 | GATA binding protein 4 | gata4 |
| Str.16319.2.S1_at | 6,68 | hypothetical protein LOC100489354 | LOC100489354 |
| Str.11968.1.A1_at | 6,63 | RAS, dexamethasone-induced 1 | rasd1 |
| Str.10727.1.S1_at | 6,63 | deoxyribonuclease gamma-like | LOC100497175 |
| Str.10803.1.S1_s_at | 6,62 | goosecoid homeobox hypothetical LOC100135186 | gsc LOC100135186 |
| Str.12750.1.A1_at | 6,62 | LIM homeobox 1 | lhx1 |
| Str.1716.1.S1_x_at | 6,58 | purinergic receptor P2Y, G-protein coupled, 2 | p2ry2 |
| Str.27405.1.A1_at | 6,47 | WNT1 inducible signaling pathway protein 3 | wisp3 |
| Str.15223.4.S1_a_at | 6,42 | eukaryotic translation initiation factor 4A2 | eif4a2 |
| Str.8895.3.S3_at | 6,42 | GATA-binding factor 2-like | LOC100487142 |
| StrEns.9366.1.S1_a_at | 6,38 | oocyte zinc finger protein XlCOF6-like | LOC100495878 |
| Str.36919.1.S1_at | 6,37 | VENT homeobox 3, gene 1 | ventx3.1 |
| Str.1534.1.S2_at | 6,35 | hypothetical protein hypothetical protein hypothetical protein | LOC100489830 LOC100494885 LOC100495042 |
| Str.15023.1.S1_a_at | 6,33 | dickkopf 1 | dkk1 |
| Str.1273.1.S1_at | 6,28 | histone H4-like | LOC100496593 |
| Str.52067.1.S1_s_at | 6,28 | zinc finger protein 568 | znf568 |
| Str.51804.1.S1_at | 6,27 | chemokine (C-X-C motif) receptor 7 | cxcr7 |
| Str.27170.2.A1_x_at | 6,27 | hypothetical protein LOC100145554 | LOC100145554 |
| Str.488.1.S1_at | 6,26 | hypothetical protein MGC76328 | MGC76328 |
| Str.16849.2.S1_at | 6,25 | histidine ammonia-lyase, gene 1 | hal.1 |
| Str.27139.1.S1_at | 6,22 | PR domain containing 1, with ZNF domain | prdm1 |
| Str.27989.1.S1_at | 6,21 | hypothetical protein LOC100493710 | LOC100493710 |
| Str.8101.1.S1_at | 6,19 | ferritin, heavy polypeptide 1 | fth1 |
| Str.6630.1.S2_at | 6,18 | X-box binding protein 1 | xbp1 |
| Str.17625.1.S1_at | 6,14 | hypothetical LOC100495344 | LOC100495344 |
| Str.4744.1.S1_at | 6,11 | dual specificity phosphatase 5 | dusp5 |
| Str.6659.1.S1_at | 6,11 | nerve growth factor receptor | ngfr |
| Str.8646.3.S1_a_at | 6,11 | inhibitor of DNA binding 3, dominant negative helix-loop-helix protein | id3 |
| Str.21522.1.S1_at | 6,10 | uroplakin 2 | upk2 |
| Str.42133.2.A1_at | 6,09 | hypothetical LOC100486754 | LOC100486754 |
| Str.27583.3.S1_at | 6,07 | MGC89648 protein | MGC89648 |
| Str.266.1.S1_at | 6,06 | chemokine (C-X-C motif) receptor 4 | cxcr4 |
| Str.42133.1.S1_x_at | 6,02 | zinc finger protein 33A | znf33a |
| Str.51679.2.S2_at | 6,00 | hypothetical protein hypothetical protein | LOC100498064 LOC100498497 |
| Str.51828.3.S1_s_at | 5,99 | hypothetical protein LOC100489130 | LOC100489130 |
| Str.3160.2.S1_a_at | 5,97 | serine/arginine-rich splicing factor 6 | srsf6 |
| Str.26882.1.S1_at | 5,96 | siamois homeodomain 1 | sia1 |
| Str.10008.1.S1_at | 5,90 | hypothetical protein LOC100487199 | LOC100487199 |
| Str.12012.1.S1_at | 5,88 | ankyrin repeat domain 10 | ankrd10 |
| Str.27186.1.S1_s_at | 5,84 | histone H3.2-like | LOC100496129 |
| Str.52193.1.S1_at | 5,83 | High-mobility group nucleosomal binding domain 2 | TEgg003n10.1 |
| StrJgi.8558.1.S1_s_at | 5,81 | hypothetical protein hypothetical protein hypothetical protein hypothetical protein hypothetical protein | LOC100486020 LOC100488381 LOC100493631 LOC100494089 LOC100498455 |
| Str.43435.1.A1_s_at | 5,81 | heart and neural crest derivatives expressed 2 | hand2 |
| StrEns.11954.1.S1_a_at | 5,79 | hypothetical protein LOC100494960 | LOC100494960 |
| Str.9440.1.S1_at | 5,77 | activated leukocyte cell adhesion molecule | alcam |
| Str.37384.1.S1_at | 5,69 | frizzled homolog 8 | fzd8 |
| Str.33.1.S1_at | 5,67 | protein kinase domain containing, cytoplasmic homolog, gene 1 | pkdcc.1 |
| StrEns.171.1.S1_s_at | 5,65 | phosphatidic acid phosphatase type 2B | ppap2b |
| Str.50024.1.S1_at | 5,64 | serine dehydratase | sds |
| Str.49462.1.S1_at | 5,60 | T-box 2 | tbx2 |
| Str.30427.1.S1_at | 5,57 | StAR-related lipid transfer (START) domain containing 13 | stard13 |
| Str.48764.1.A1_at | 5,56 | Mdm2 p53 binding protein homolog | mdm2 |
| Str.11235.2.S1_at | 5,52 | hypothetical protein LOC100145165 | LOC100145165 |
| Str.31298.1.S1_at | 5,50 | iroquois homeobox 1 | irx1 |
| Str.10459.1.A1_at | 5,49 | Ubiquitin-conjugating enzyme E2D 3 (UBC4/5 homolog) | ube2d3 |
| Str.6149.1.S1_at | 5,47 | glutamine--fructose-6-phosphate transaminase 1 | gfpt1 |
| Str.51121.1.S1_at | 5,45 | Kruppel-like factor 17 | klf17 |
| Str.23519.2.S1_a_at | 5,44 | Death inducer-obliterator 1 | dido1 |
| Str.6018.1.S1_at | 5,43 | hairy and enhancer of split 4 | hes4 |
| Str.26907.1.S1_at | 5,43 | nodal homolog 1 | nodal1 |
| Str.6304.1.S2_at | 5,42 | calponin 1, basic, smooth muscle | cnn1 |
| Str.36162.1.A1_at | 5,41 | hypothetical protein LOC100498210 | LOC100498210 |
| Str.20470.1.S2_at | 5,38 | cyclin E2 | ccne2 |
| StrAffx.22.1.S1_at | 5,37 | hypothetical protein hypothetical protein | LOC100486038 LOC100487146 |
| Str.21783.1.S1_at | 5,36 | Sec61 alpha 1 subunit (S. cerevisiae) | sec61a1 |
| Str.41326.1.S1_s_at | 5,33 | hairy and enhancer of split 6, gene 1 | hes6.1 |
| Str.1865.1.S1_at | 5,30 | cerberus 1, cysteine knot superfamily | cer1 |
| Str.18694.2.S1_x_at | 5,29 | hypothetical protein LOC100498107 | LOC100498107 |
| Str.2177.1.A1_a_at | 5,26 | sphingosine-1-phosphate receptor 5 | s1pr5 |
| Str.20538.3.A1_at | 5,24 | hypothetical protein LOC100497342 | LOC100497342 |
| Str.17388.1.S1_at | 5,24 | hypothetical LOC100495861 | LOC100495861 |
| Str.27013.1.S1_a_at | 5,22 | uroplakin 3B | upk3b |
| Str.37201.1.S1_at | 5,18 | melanopsin-B-like | LOC100495677 |
| Str.27847.1.A1_at | 5,16 | hypothetical protein LOC100493690 | LOC100493690 |
| Str.27924.1.S1_at | 5,14 | zinc finger protein ZIC 4-like | LOC100493609 |
| Str.48741.1.S1_x_at | 5,13 | Peptidyl-prolyl isomerase G (cyclophilin G) | ppig |
| Str.16957.1.S1_at | 5,12 | fused in sarcoma | fus |
| Str.11165.7.S1_at | 5,10 | sarcoplasmic/endoplasmic reticulum calcium ATPase 2-like | LOC100489059 |
| StrJgi.6845.1.S1_x_at | 5,08 | oocyte zinc finger protein XlCOF7.1-like | LOC100495340 |
| Str.20538.1.S2_a_at | 5,07 | KH domain containing, RNA binding, signal transduction associated 1 | khdrbs1 |
| Str.420.1.S2_a_at | 5,07 | ABO blood group (transferase A, alpha 1-3-N-acetylgalactosaminyltransferase; transferase B, alpha 1-3-galactosyltransferase) | abo |
| Str.37703.1.S1_at | 5,04 | transportin 1 | tnpo1 |
| StrEns.4645.1.S1_at | 5,04 | nodal homolog 4-A-like | LOC100497218 |
| Str.24746.1.S1_at | 5,04 | solute carrier family 43, member 1 | slc43a1 |
| StrEns.8996.1.S1_a_at | 5,03 | hypothetical protein LOC100496030 | LOC100496030 |
| Str.10535.1.S1_at | 4,98 | hypothetical LOC100495377 | LOC100495377 |
| Str.8191.1.S2_at | 4,96 | phospholipase A2, group XIIB | pla2g12b |
| StrJgi.784.1.S1_s_at | 4,95 | retinoblastoma-like 1 (p107) | rbl1 |
| Str.5015.1.A1_at | 4,95 | neural precursor cell expressed, developmentally down-regulated 9 | nedd9 |
| Str.37936.1.A1_at | 4,92 | fibroblast growth factor 20 | fgf20 |
| Str.111.1.S1_at | 4,91 | hematopoietically expressed homeobox | hhex |
| Str.16175.1.S1_a_at | 4,90 | growth differentiation factor 3 | gdf3 |
| Str.9391.1.A1_at | 4,90 | Hypothetical protein LOC549444 | LOC549444 |
| Str.50563.1.A1_at | 4,90 | Hypothetical protein LOC100216216 | LOC100216216 |
| Str.6578.1.S2_a_at | 4,86 | serine/arginine-rich splicing factor 1 | srsf1 |
| Str.6723.1.S1_x_at | 4,84 | pinhead | pnhd |
| Str.154.1.S1_at | 4,84 | frizzled homolog 10 | fzd10 |
| Str.18694.1.S1_at | 4,84 | uncharacterized protein ywlC-like | LOC100497798 |
| Str.20533.6.S1_x_at | 4,83 | hypothetical LOC100486606 | LOC100486606 |
| Str.28734.1.A1_at | 4,81 | hypothetical protein LOC100489967 | LOC100489967 |
| Str.40271.2.S1_a_at | 4,80 | Hypothetical protein LOC550005 | LOC550005 |
| Str.16161.1.S1_at | 4,80 | sizzled | szl |
| Str.11217.1.S1_at | 4,78 | MGC89906 protein | MGC89906 |
| Str.10785.1.S1_at | 4,77 | hypothetical LOC100497479 | LOC100497479 |
| Str.1843.1.S1_at | 4,76 | v-ski sarcoma viral oncogene homolog | ski |
| Str.10305.2.S1_at | 4,74 | hypothetical protein LOC100216037 | LOC100216037 |
| Str.18775.1.S1_at | 4,69 | CKLF-like MARVEL transmembrane domain containing 8 | cmtm8 |
| Str.3002.1.A1_at | 4,67 | Hypothetical protein LOC779546 | LOC779546 |
| Str.43058.1.S1_at | 4,60 | zinc finger protein | LOC100337651 |
| Str.1183.1.S1_at | 4,56 | transmembrane protein 127 | tmem127 |
| Str.6139.1.S2_at | 4,55 | GATA binding protein 6 | gata6 |
| Str.7077.1.A1_at | 4,55 | hormonally up-regulated Neu-associated kinase | hunk |
| Str.7705.5.S1_at | 4,51 | odd-skipped related 2 | osr2 |
| Str.28286.1.S1_at | 4,51 | RNA binding motif protein 25 | rbm25 |
| Str.15853.1.S1_at | 4,49 | pleckstrin homology domain-containing family N member 1-like | LOC100491834 |
| Str.6156.1.S1_a_at | 4,49 | forkhead box C1 | foxc1 |
| StrJgi.7085.1.S1_s_at | 4,49 | PR domain containing 9 | prdm9 |
| Str.18694.2.S1_a_at | 4,49 | uncharacterized protein ywlC-like hypothetical protein | LOC100497798 LOC100498107 |
| Str.15587.1.S1_at | 4,48 | hypothetical protein LOC100487200 | LOC100487200 |
| Str.1567.1.S2_at | 4,47 | ST6 (alpha-N-acetyl-neuraminyl-2,3-beta-galactosyl-1,3)-N-acetylgalactosaminide alpha-2,6-sialyltransferase 2 | st6galnac2 |
| Str.28803.2.S1_a_at | 4,47 | hypothetical LOC100486098 | LOC100486098 |
| Str.37570.1.S1_at | 4,45 | solute carrier family 7 (cationic amino acid transporter, y+ system), member 3 | slc7a3 |
| Str.51825.1.S1_at | 4,44 | Hypothetical protein LOC733820 | LOC733820 |
| Str.10072.1.S2_at | 4,43 | Solute carrier family 2 (facilitated glucose transporter), member 2 | slc2a2 |
| Str.24244.1.S1_at | 4,41 | SRY (sex determining region Y)-box 21 | sox21 |
| Str.31333.1.S1_a_at | 4,40 | protein kinase domain containing, cytoplasmic homolog, gene 2 | pkdcc.2 |
| Str.11402.1.A1_at | 4,35 | protein phosphatase 1, regulatory (inhibitor) subunit 3C, gene 1 | ppp1r3c.1 |
| Str.10016.1.S1_at | 4,35 | cdc42 effector protein (Rho GTPase binding) 4 | cdc42ep4 |
| Str.10174.1.A1_at | 4,32 | single-strand-selective monofunctional uracil-DNA glycosylase 1 | smug1 |
| Str.10713.1.S1_at | 4,30 | growth arrest-specific 1 | gas1 |
| Str.14504.1.S1_at | 4,29 | rho GTPase-activating protein 39-like | LOC100485974 |
| Str.1093.1.S1_at | 4,26 | ADP-ribosylation factor-like 4C | arl4c |
| Str.9958.1.S1_at | 4,25 | chromosome 7 open reading frame 11 | c7orf11 |
| Str.954.1.S1_at | 4,24 | potassium channel tetramerisation domain containing 15 | kctd15 |
| Str.5982.1.S1_a_at | 4,22 | darmin | darmin |
| StrEns.1208.1.S1_at | 4,18 | nodal homolog 2-A-like | LOC100496994 |
| Str.21565.4.S1_at | 4,17 | RNA binding motif protein, X-linked | rbmx |
| Str.24254.1.S1_at | 4,15 | wingless-type MMTV integration site family, member 5B | wnt5b |
| Str.5804.3.S1_a_at | 4,12 | hypothetical LOC100487483 hypothetical LOC100490010 hypothetical LOC100490596 hypothetical LOC100491185 hypothetical LOC100492994 hypothetical LOC100493564 | LOC100487483 LOC100490010 LOC100490596 LOC100491185 LOC100492994 LOC100493564 |
| Str.26607.1.S1_at | 4,11 | protein unc-80 homolog | LOC100492079 |
| Str.6167.1.S1_at | 4,09 | phosphorylase, glycogen, muscle | pygm |
| Str.26626.1.S1_at | 4,06 | growth arrest and DNA-damage-inducible, alpha | gadd45a |
| Str.7519.1.S1_at | 4,01 | ras homolog gene family, member B | rhob |
| Str.916.1.S1_at | 4,00 | lin-28 homolog A (C. elegans) | lin28a |
| Str.51815.1.S1_at | 3,98 | notochord homeobox | not |
| Str.1180.1.S2_at | 3,98 | forkhead box A1 | foxa1 |
| Str.15005.1.S1_at | 3,98 | hairy and enhancer of split 3, gene 1 | hes3.1 |
| Str.27547.1.S3_at | 3,96 | hypothetical protein LOC550042 | LOC550042 |
| Str.4965.1.S1_at | 3,96 | minichromosome maintenance complex component 6 | mcm6.2 |
| Str.3344.1.S1_at | 3,94 | novel protein similar to hatching enzymes | LOC594901 |
| Str.993.1.S1_a_at | 3,91 | mex-3 homolog C | mex3c |
| Str.11106.1.S1_at | 3,90 | hypothetical protein LOC100488554 | LOC100488554 |
| Str.35184.1.A1_s_at | 3,88 | capping protein (actin filament) muscle Z-line, alpha 1 | capza1 |
| Str.643.1.S1_a_at | 3,88 | hypothetical LOC100486597 | LOC100486597 |
| Str.5849.2.A1_s_at | 3,87 | zinc finger E-box binding homeobox 2 | zeb2 |
| Str.16322.1.S1_at | 3,86 | Sp9 transcription factor homolog | sp9 |
| Str.514.1.S1_at | 3,83 | pim-1 oncogene | pim1 |
| Str.28107.1.S1_at | 3,83 | snail homolog 1 | snai1 |
| Str.51836.1.S1_at | 3,80 | angiopoietin 4 | angpt4 |
| Str.15072.2.A1_a_at | 3,77 | delta-like 1 | dll1 |
| Str.24583.1.S1_at | 3,75 | leucine-rich repeats and immunoglobulin-like domains 3 | lrig3 |
| Str.15996.4.S1_at | 3,74 | hypothetical LOC100490120 | LOC100490120 |
| Str.13766.1.S1_at | 3,73 | zinc finger protein 608 | znf608 |
| Str.27389.1.S1_at | 3,69 | transmembrane protein 150B | tmem150b |
| Str.7002.1.S1_at | 3,69 | hypothetical LOC100497373 | LOC100497373 |
| Str.21604.1.S1_at | 3,69 | 5'-nucleotidase, cytosolic III | nt5c3 |
| StrJgi.5287.1.S1_s_at | 3,68 | kin of IRRE like 2 | kirrel2 |
| StrJgi.6058.1.S1_at | 3,67 | LIM/homeobox protein Lhx3-like | LOC100496651 |
| Str.3082.1.S1_at | 3,62 | wnt11 protein | wnt11 |
| Str.5394.1.S1_at | 3,61 | carboxylesterase 2 | ces2 |
| Str.11087.1.S1_at | 3,60 | distal-less homeobox 5 | dlx5 |
| Str.20011.1.S1_at | 3,59 | receptor tyrosine kinase-like orphan receptor 2 | ror2 |
| Str.49050.1.S1_at | 3,58 | Fast skeletal myosin light chain 2 | TNeu107a14.1 |
| Str.10659.1.S1_at | 3,57 | transcription factor AP-2 epsilon (activating enhancer binding protein 2 epsilon) | tfap2e |
| Str.24871.1.S1_x_at | 3,56 | zinc finger protein 33B | znf33b |
| Str.6103.1.S1_at | 3,55 | MID1 interacting protein 1 (gastrulation specific G12 homolog) | mid1ip1 |
| Str.10076.1.S1_at | 3,52 | angiomotin like 2 | amotl2 |
| Str.42133.3.A1_x_at | 3,52 | zinc finger protein 502-like gastrula zinc finger protein XlCGF57.1-like zinc finger protein 613-like MGC146893 zinc finger protein 3 zinc finger protein 33A zinc finger protein 33B zinc finger protein 665 | LOC100485089 LOC100489449 LOC100493688 MGC146893 znf3 znf33a znf33b znf665 |
| Str.24842.3.S1_at | 3,51 | ropporin 1-like | ropn1l |
| Str.11288.1.S1_at | 3,51 | hypothetical LOC100486074 | LOC100486074 |
| Str.13147.1.A1_at | 3,50 | zinc finger protein 219-like | LOC100495520 |
| Str.21950.2.S1_a_at | 3,49 | hypothetical LOC100488570 | LOC100488570 |
| Str.16343.1.A1_at | 3,48 | Hypothetical protein LOC100145152 | LOC100145152 |
| Str.10208.1.S1_at | 3,47 | solute carrier family 12 (sodium/chloride transporters), member 3, gene 2 | slc12a3.2 |
| Str.9193.1.A1_a_at | 3,46 | UPF0632 protein C2orf89-like | LOC100491951 |
| Str.6363.1.S2_at | 3,45 | acidic (leucine-rich) nuclear phosphoprotein 32 family, member C | anp32c |
| Str.49171.3.S1_at | 3,45 | hypothetical LOC100488154 | LOC100488154 |
| Str.38997.1.A1_at | 3,42 | proprotein convertase subtilisin/kexin type 9-like | LOC100484989 |
| Str.10900.1.S1_at | 3,41 | serum/glucocorticoid regulated kinase 1 | sgk1 |
| Str.6695.1.S2_at | 3,40 | zinc finger, MIZ-type containing 2 | zmiz2 |
| Str.39816.2.S1_at | 3,28 | hypothetical LOC100488648 | LOC100488648 |
| Str.21565.6.A1_at | 3,28 | hypothetical LOC100485563 | LOC100485563 |
| Str.7820.2.S1_a_at | 3,24 | immediate early response gene 2 protein-like | LOC100491464 |
| Str.29569.1.S1_at | 3,23 | Meningioma (disrupted in balanced translocation) 1, gene 1 | mn1.1 |
| Str.2514.1.S1_a_at | 3,22 | NIMA (never in mitosis gene a)-related kinase 2 | nek2 |
| Str.22063.1.S1_at | 3,20 | hypothetical LOC100489698 | LOC100489698 |
| Str.51755.2.A1_a_at | 3,19 | hypothetical protein LOC100491437 | LOC100491437 |
| Str.10330.1.S2_at | 3,16 | sprouty homolog 1, antagonist of FGF signaling | spry1 |
| Str.2247.1.S1_at | 3,15 | RAB34, member RAS oncogene family | rab34 |
| Str.51988.1.S1_at | 3,14 | serine/arginine-rich splicing factor 11 | srsf11 |
| Str.33742.1.A1_a_at | 3,13 | hypothetical protein LOC100494123 | LOC100494123 |
| Str.51680.1.A1_at | 3,12 | zinc finger protein 721 | znf721 |
| Str.15643.1.A1_at | 3,10 | hypothetical protein LOC100145695 | LOC100145695 |
| Str.1973.2.A1_a_at | 3,05 | huntingtin-associated protein 1 | hap1 |
| Str.10123.1.S1_at | 3,02 | glucose-fructose oxidoreductase domain containing 1 | gfod1 |
| Str.16252.1.S1_at | 3,02 | SRY (sex determining region Y)-box 1 | sox1 |
| Str.10371.1.S1_at | 3,00 | chromosome 8 open reading frame 4 | c8orf4 |
| Str.51787.1.S1_at | 3,00 | histone cluster 1, H2ah | hist1h2ah |
| Str.40984.1.S1_at | 2,98 | hypothetical protein LOC100494082 | LOC100494082 |
| Str.27184.1.S1_a_at | 2,98 | matrix metallopeptidase 1 (interstitial collagenase) | mmp1 |
| Str.51982.1.A1_s_at | 2,96 | iroquois homeobox 3 | irx3 |
| Str.11815.1.S1_at | 2,95 | protein phosphatase 1, regulatory (inhibitor) subunit 15B | ppp1r15b |
| Str.40960.1.S1_at | 2,95 | zinc finger, SWIM-type containing 5 | zswim5 |
| Str.2514.4.A1_a_at | 2,94 | NIMA (never in mitosis gene a)-related kinase 2 | nek2 |
| Str.30747.1.A1_at | 2,94 | regulator of G-protein signaling 16 | rgs16 |
| Str.4877.1.S1_at | 2,94 | caudal type homeobox 4 | cdx4 |
| StrJgi.6297.1.S1_at | 2,92 | homeobox protein siamois-like | LOC100489935 |
| Str.15740.1.A1_at | 2,92 | MAP kinase interacting serine/threonine kinase 2 | mknk2 |
| Str.50722.6.S1_at | 2,90 | transient receptor potential cation channel subfamily M member 4-like | LOC100491327 |
| Str.49514.1.S1_at | 2,89 | T-box 3 | tbx3 |
| Str.49437.1.S1_at | 2,82 | Hypothetical protein LOC100144964 | LOC100144964 |
| Str.30700.3.A1_s_at | 2,82 | solute carrier family 41, member 2 | slc41a2 |
| Str.169.2.S1_a_at | 2,81 | sal-like 1 | sall1 |
| Str.23452.1.A1_at | 2,79 | empty spiracles homeobox 1 | emx1 |
| Str.40558.2.A1_at | 2,79 | Hypothetical protein LOC100158524 | LOC100158524 |
| Str.246.1.S1_at | 2,79 | mab-21-like 2 | mab21l2 |
| Str.8107.1.S1_at | 2,77 | forkhead box A2 | foxa2 |
| Str.27386.1.S1_at | 2,74 | RasGEF domain family, member 1B | rasgef1b |
| Str.10824.1.S1_at | 2,74 | KIT ligand | kitlg |
| Str.6084.1.S2_at | 2,73 | lymphocyte antigen 6 complex, similar to G6C | ly6g6c |
| Str.27109.1.S1_at | 2,72 | oligodendrocyte transcription factor 3 | olig3 |
| Str.2696.1.S1_at | 2,71 | forkhead box C2 (MFH-1, mesenchyme forkhead 1) | foxc2 |
| Str.5969.1.S1_at | 2,70 | CDGSH iron sulfur domain 1 | cisd1 |
| Str.28780.1.S1_at | 2,65 | RAS-like, family 11, member B | rasl11b |
| Str.15404.1.S1_at | 2,64 | carbohydrate (N-acetylglucosamine-6-O) sulfotransferase 2 | chst2 |
| StrJgi.8558.1.S1_x_at | 2,63 | hypothetical protein LOC100494089 | LOC100494089 |
| Str.10666.1.S1_at | 2,63 | follistatin | fst |
| Str.19278.2.S1_at | 2,61 | hypothetical protein LOC100491368 | LOC100491368 |
| Str.2504.2.S1_at | 2,59 | RNA binding motif protein 6 | rbm6 |
| Str.5989.1.S1_at | 2,59 | retinoblastoma binding protein 6 | rbbp6 |
| Str.10221.1.S2_at | 2,58 | hypothetical LOC100495650 | LOC100495650 |
| Str.11224.1.S1_at | 2,57 | zinc finger protein 238.2-like | LOC100489646 |
| StrEns.4639.1.S1_s_at | 2,56 | arachidonate 5-lipoxygenase-activating protein | alox5ap |
| Str.20309.1.A1_at | 2,54 | cyclin-dependent kinase 6 | cdk6 |
| Str.24871.1.S2_s_at | 2,54 | zinc finger protein 613-like | LOC100493688 |
| Str.11302.1.S1_at | 2,54 | forkhead box D3 | foxd3 |
| Str.8068.3.S1_at | 2,53 | Solute carrier family 5 (iodide transporter), member 8 | slc5a8 |
| Str.303.1.S1_at | 2,50 | G protein-coupled receptor, family C, group 5, member C | gprc5c |
