# Supplementary material for: Brg1 chromatin remodeling ATPase balances germ layer patterning by amplifying the transcriptional burst at midblastula transition
Source: PLoS Genet. 2017 May 12;13(5):e1006757. doi: 10.1371/journal.pgen.1006757 (PMC5428918; doi:10.1371/journal.pgen.1006757)
Supplement: S5 Table — (DOCX) [file pgen.1006757.s015.docx]

#### Table S5a: Oligonucleotides for cloning

| **plasmid** |  | **vector** | **5’-sequence-3’** |
| --- | --- | --- | --- |
| XhoI_Luciferase | *for* | pCS2+GW | -CA CTCGAG_GAAGACGCCAAAAACATAAA- |
| Luciferase_XbaI | *rev* | pCS2+GW | -CG TCTAGA_CACGGCGATCTTTCC- |
| attB1_BISH | *for* | pCS2+GW | -GGGACAAGTTTGTACAAAAAAGCAGGCTTAACT  GATAGTTGGTGGAAAACGTCAGAAGGA- |
| BISH_attB2 | *rev* | pCS2+GW | -GGGGACCACTTTGTACAAGAAAGCTGGGTC TCCCATCATGCTATGTGCGG- |

#### Table S5b: Oligonucleotides for quantitative RT-PCR

| **gene** |  | **5’-sequence-3’** |
| --- | --- | --- |
| *bmp4* | *for* | -agcccagtaaggatgtggtg- |
| *bmp4* | *rev* | -ggtctctccgggtactccat- |
| *cerberus* | *for* | -tccatgttccaaatcagcaa- |
| *cerberus* | *rev* | -aattcagtgccaggtggttc- |
| *chordin* | *for* | -TCTGCTATACAGCGGCCTTT- |
| *chordin* | *rev* | -TACCCAGACCAGTCACCACA- |
| *crbp* | *for* | -CTGGCAAGTCCTCCAATGATA- |
| *crbp* | *rev* | -CTCTGTCTCCACCACCACCT- |
| *crx* | *for* | -CCCACGGAAACAAAGAAGAG- |
| *crx* | *rev* | -TTTAGCAAAGAGGGCTTCCA- |
| *egr1* | *for* | -CTCTCACACCCCTGTCTACCA- |
| *egr1* | *rev* | -CATTCTGCTTGGCTTGATGA- |
| *follistatin* | *for* | -TACCCAACAGCACCCTCTTC- |
| *follistatin* | *rev* | -GGGCCACAGTCTACGTTCTC- |
| *foxc1* | *for* | -GACCCGGACTCGTACAACAT- |
| *foxc1* | *rev* | -CTTTCAGGAGCCGATCTTTG- |
| *foxd3* | *for* | -CCCAGGCAAGGGAAACTACT- |
| *foxd3* | *rev* | -AATGAGTCCGGCTGTTGTCT- |
| *foxd4l1* | *for* | -ACCATGCCAACTCACCTTTC- |
| *foxd4l1* | *rev* | -TTATCAGCATCCAGGGCTTC- |
| *foxi1* | *for* | -TCCTCAGCGACCTTCAAACT- |
| *foxi1* | *rev* | -CATGAAATGGCTTGTGTTGG- |
| *foxi4.2* | *for* | -CTCCTACTCTGCCCTCATCG- |
| *foxi4.2* | *rev* | -AGCCTGCTTTGCTCTTTTTG- |
| *gapdh* | *for* | -acccagaagactgtggatgg- |
| *gapdh* | *rev* | -Gttgaggcagggatgatgtt- |
| *gata4* | *for* | -ACGAAAGCGCAAACCTAAGA- |
| *gata4* | *rev* | -GAGCTGGTGGAAGGAGTGAG- |
| *gs17* | *for* | -AGGGGGCATAAAGAGTGGTC- |
| *gs17* | *rev* | -CGAGGAGCAAACTGTGGAGT- |
| *hes4* | *for* | -AAGTCACCCGATTCCTCTCC- |
| *hes4* | *rev* | -GGGTTGCTGGTAATTCATGG- |
| *jhdm1* | *for* | -TTAAAGCATGGGGTGAAAGC- |
| *jhdm1* | *rev* | -GAGATGCCCAGGTCTGATGT- |
| *kiaa1279* | *for* | -GGCTGCAAAAGAGAATGAGG- |
| *kiaa1279* | *rev* | -TTTTACCCAACATCGTGCAA- |
| *Irx2* | *for* | -AATCACCAACAGGACGGAAG- |
| *Irx2* | *rev* | -GATCCGAGGTGGCTATTTCA- |
| *meis3* | *for* | -TTATGGGCACCCTCTGTTTC- |
| *meis3* | *rev* | -TGGGAATGACCCTGAATTGT- |
| *noggin* | *for* | -cggaggagagacttggagtg- |
| *noggin* | *rev* | -ctgtgctttttgccctgaa- |
| *nr6a1* | *for* | -CAGAAAGCAATCAGCCTTCC- |
| *nr6a1* | *rev* | -AGTGAGCCGAATCCATTGAG- |
| *oct25* | *for* | -AGGGGACGCTGGAAAGTTAC- |
| *oct25* | *rev* | -CCTTGGCTATTTGCACCATC- |
| *odc* | *for* | -Ccctggttcagaggacgtta- |
| *odc* | *rev* | -Agtatctcccaggctcagca- |
| *otx2* | *for* | -CAGCACCTCAGTTCCAGTCA- |
| *otx2* | *rev* | -TCATGGGGTAAGACCTCTGC- |
| *prdm1* | *for* | -TGTCATAAGCGGTTCAGCAG- |
| *prdm1* | *rev* | -TGCACTGGTAGGGCTTCTCT- |
| *siamois* | *for* | -GAACGGAGGGAATGTAAGAGG- |
| *siamois* | *rev* | -TCTGAAGGAAGTGGGTTTGC- |
| *sizzled* | *for* | -CCTGATGGGACACACTAGCA- |
| *sizzled* | *rev* | -CCGGTCTGTAGGAGGTTCTG- |
| *sox1* | *for* | -AGAACCCCAAGATGCACAAC- |
| *sox1* | *rev* | -GCCTCCGACATAACTTTCCA- |
| *sox2* | *for* | -AGATGGCTCAGGAGAACCC- |
| *sox2* | *rev* | -TCGTCGATGAATGGTCTTTTC- |
| *sox11* | *for* | -CAAGAAATGCCCCAAGCTAA- |
| *sox11* | *rev* | -GGACTTGATGGTGAGCGACT- |
| *sox21* | *for* | -GAGGAAGCCCAAGACCCTAC- |
| *sox21* | *rev* | -CCATGTAACCCAGCAACCTT- |
| *tgif* | *for* | -CTCGCCCTTCTGTGATCTGT- |
| *tgif* | *rev* | -TAAGTTGCTCGCTTCGGTCT- |
| *twin* | *for* | -ACACAGCACTGACCCTACAAGA- |
| *twin* | *rev* | -GATCAGGAATCAGGCCAAAA- |
| *xnr3* | *for* | -ACGTATTTGCCTCCCTTCCT- |
| *xnr3* | *rev* | -TCCTTTGTGTCCTTGGTATGG- |
| *xnr5* | *for* | -TCAGGCTCCTCATGGAAAGT- |
| *xnr5* | *rev* | -TGCTTGTGAAGTTGCCTTTG- |
| *xnr6* | *for* | -AAACCGTCTGCCAATCTGAC- |
| *xnr6* | *rev* | -TGGTTCCTTCCTCGATCTTG- |
| *zic1* | *for* | -gacactACGGGCCACTTGAT- |
| *zic1* | *rev* | -AGCCTCATCTGTCCGTTCAC- |
| *zic2* | *for* | -tacttttccccggcatacac- |
| *zic2* | *rev* | -tccgtcctaccgaacatctc- |
| *zic3* | *for* | -TGAACCGTCCTCAGAAAACC- |
| *zic3* | *rev* | -GGACATTCCTCCCAGTAGCA- |
|  |  |  |
